# Orthogonal functionalisation of α-helix mimetics†
†Electronic supplementary information (ESI) available: Additional binding data and fluorescence characterisation. Experimental procedures and characterisation of all new compounds. See DOI: 10.1039/c4ob00915k



**DOI:** 10.1039/c4ob00915k

**Published:** 2014-07-28

**Authors:** Anna Barnard, Kérya Long, David J. Yeo, Jennifer A. Miles, Valeria Azzarito, George M. Burslem, Panchami Prabhakaran, Thomas A. Edwards, Andrew J. Wilson

**Affiliations:** a School of Chemistry , University of Leeds , Woodhouse Lane , Leeds , LS2 9JT , UK; b Astbury Centre for Structural and Molecular Biology , University of Leeds , Woodhouse Lane , Leeds , LS2 9JT , UK . Email: A.J.Wilson@leeds.ac.uk; c School of Molecular and Cellular Biology , University of Leeds , Woodhouse Lane , Leeds , LS2 9JT , UK

## Abstract

α-Helix mediated protein–protein interactions are of major therapeutic importance. As such, the design of inhibitors of this class of interaction is of significant interest. We present methodology to modify *N*-alkylated aromatic oligoamide α-helix mimetics using ‘click’ chemistry. The effect is shown to modulate the binding properties of a series of selective p53/*h*DM2 inhibitors.

## Abstract 2

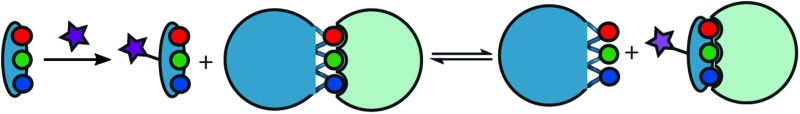
We present methodology to modify *N*-alkylated aromatic oligoamide α-helix mimetics using ‘click’ chemistry.

Inhibition of protein–protein interactions (PPIs) represents a considerable challenge in modern chemical biology.^1,2^ PPIs mediate multiple critical biological processes and are prevalent in disease pathways.^3^ When compared to traditional enzyme-substrate drug targets, PPIs are more complex due to the wide range of topographies and larger surface areas (>1000 Å^2^) present at the binding interfaces.^4^ However, it is often possible to identify ‘hot-spot’ residues which account for the majority of the binding energy.^5^ α-Helices are the most abundant secondary structural motif and frequently mediate PPIs.^6^ Several classes of inhibitors have thus been designed^7^ against this type of PPI, including β-peptides,^8^ mixed α/β-peptides^9,10^ and constrained peptides.^11–17^ Proteomimetics mimic the spatial arrangement of key binding residues on an α-helix (Fig. 1a).^18–30^ Within this family, aromatic oligoamides have emerged as a promising class of helix-mimetic.^31–33^ They have been shown to be synthetically accessible^34^ including by solid-phase synthesis^35,36^ and their side chains are projected in a manner which effectively recapitulates the spatial orientation of ‘hot-spot’ residues on a helical scaffold. To utilise helix mimetics and tailor their properties for cellular studies (*e.g.* cellular imaging, optimising cell uptake *etc.*) and/or applications in hit identification (*e.g.* immobilisation of libraries on surfaces), it is necessary to functionalise the ligands in an orthogonal manner and with minimal perturbation of the helix mimicking epitope (Fig. 1e). Herein we describe a strategy to achieve such a goal, through functionalisation of the non-helix mimicking face of *N*-alkylated aromatic oligoamides (Fig. 1c), by ‘click’ chemistry. Our results illustrate that, the selectivity profile of the mimetic is retained as a consequence of the modifications. The p53/*h*DM2 interaction represents a PPI under intense current investigation^37^ (Fig. 1b); it exploits residues Phe19, Trp23 and Leu26 on the p53 transactivation domain bound in a helical conformation to *h*DM2 (PDB ID: ; 1YCR).^38^ It thus represents an ideal model target with which to develop helix mimetics. In recent years, our group has targeted the p53/*h*DM2 interaction using both *N*- and *O*-alkylated aromatic oligoamide helix-mimetics (Fig. 1c and d).^31–33^ To improve the solubility of the *O*-alkylated series an ethylene glycol chain was introduced onto the non-binding face,^39^ which improved aqueous solubility without abrogating binding affinity. The synthesis of this modified *O*-alkylated helix mimetic proved challenging and informed our strategy in the current work; we decided to combine features of both scaffolds; the *N*-alkylated scaffold to display the binding groups and the *O*-alkyl function to install a ‘click’ chemistry functional handle. The copper(i) catalysed cyclisation between an alkyne and an azide has received considerable attention in recent years.^40^ The modified Huisgen 1,3-cyclisation^41^ developed by the groups of Sharpless and Meldal^42,43^ is high yielding and tolerant of diverse substrates and conditions.^44^

**Fig. 1:**
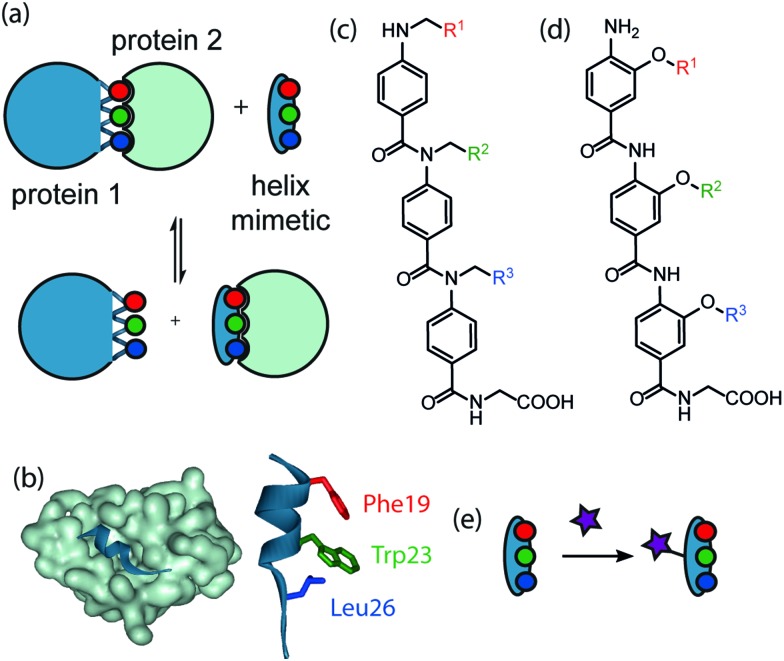
Helix mimetics as inhibitors of PPIs: (a) schematic illustrating proteomimetic concept, (b) p53/*h*DM2 interaction (PDB ID: ; 1YCR), (c) *N*- and (d) *O*-alkylated aromatic oligoamides, (e) schematic illustrating concept for orthogonal functionalisation.

Introducing a residue amenable to ‘click’ chemistry necessitated the design and synthesis of a novel monomer unit (Scheme 1). Methyl 3-hydroxy-4-nitrobenzoate, **6**, was alkylated with propargyl bromide installing the alkyne functionality. Reduction with tin(ii) chloride resulted in synthesis of the desired amine without any effect on the alkyne and, after saponification of the ester this could be *N*-alkylated. Subsequent Fmoc protection afforded monomer, **10**.

**Scheme 1:**
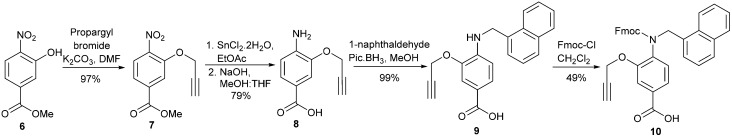
Synthesis of alkyne-functionalised *N*- and *O*-alkylated Fmoc monomer, **10**.

The ‘click-able’ monomer could be readily incorporated into a trimer structure with side chains designed to mimic the binding residues of the p53 helix. A solid-phase synthetic strategy was employed (Scheme 2).^35^ Trimer **1** (a regioisomer of our previously described most potent hit from this series),^32^ was synthesised using unfunctionalised *N*-alkylated monomers whereas trimers **2–5** utilised the alkyne monomer **10** as the central residue. The ‘click’ reaction could then be performed on resin. A variety of commercially available azides were chosen; a protected acid, methyl 2-azidoacetate, and an ethylene glycol, *O*-(2-azidoethyl)-*O*′-methyl-triethylene glycol in order to synthesise a trimer related to the ‘wet-edge’ foldamer described previously.^39^ Two novel azides were also synthesised (Scheme S1†) to append the trimers with coumarin and fluorescein fluorophores. After a copper(i) catalysed ‘click’ reaction^42,43^ the trimers were cleaved from the resin with TFA and the desired proteomimetics were isolated in excellent yield and purity (see ESI† for characterisation).

**Scheme 2:**
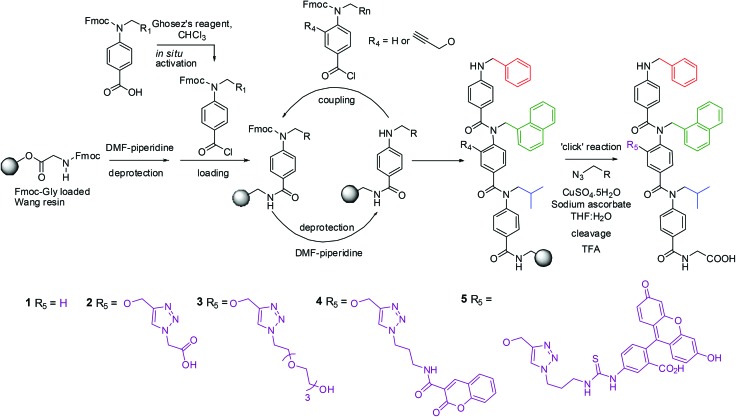
Solid-phase synthesis of triazole-functionalised trimers **1–5**.

In order to ascertain if the modifications were likely to have any effect upon the ability of the oligomers to mimic the p53 helix, molecular modeling was performed (see ESI† for details). The unfunctionalised analogue was compared with the scaffolds functionalised with a carboxylate and an ethylene glycol (Fig. 2a–c). The entire set of structures within 1.5 kJ mol^–1^ of the minimum energy conformation were superimposed onto the p53 helix with the α-CH_2_ on the trimer representing the α-carbon on the peptide. A root mean square deviation (RMSD) was calculated based on this degree of overlap. The combinations with the best overlap (lowest RMSD) are shown in Fig. 2. The modeling data show that by introducing functionality onto the non-binding face the side chain overlap is not significantly affected and these structures are still capable of effective mimicry of the p53 helix.

**Fig. 2:**
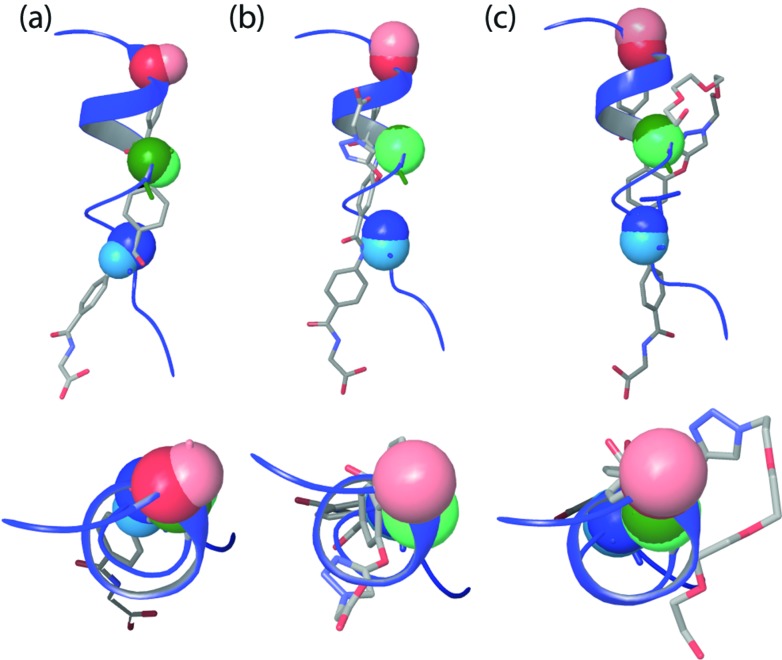
Overlay of (a) unfunctionalised trimer **1** (RMSD = 0.983 Å), (b) acid functionalised trimers **2** (RMSD = 0.772 Å) and (c) ethylene glycol-functionalised trimer **3** (RMSD = 0.753 Å) with p53. Overlaid residues are shown in CPK format. p53 residues are in dark colours and helix mimetic residues are in light colours (side and top views are given).

The ability of the trimers to inhibit the p53/*h*DM2 interaction was then investigated using a fluorescence anisotropy competition assay (Fig. 3a). Briefly, a fluorescein-labelled p53 peptide is initially bound to *h*DM2 and displaced by increasing concentrations of inhibitor leading to a measurable decrease in anisotropy from which IC_50_ values can be determined, Table 1.

**Fig. 3:**
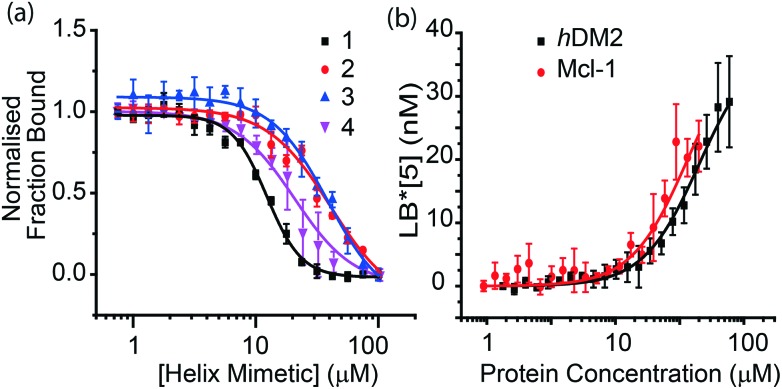
Fluorescence anisotropy binding assays in phosphate buffer pH 7.5, 200 mM NaCl, 0.02 mg ml^–1^ BSA. (a) p53/*h*DM2 competition assay *h*DM2 is fixed as 154 nM and FITC-p53 is at a concentration of 54.5 nM. (b) Direct binding assay of trimer **5** binding to *h*DM2 or Mcl-1. Compound **5** is fixed at 50 nM.

**Table 1:** IC_50_ values for trimers **1–4** obtained from fluorescence anisotropy assays against the p53/*h*DM2, Mcl-1/NOXA-B and Bcl-x_L_/BAK protein–protein interactions

Trimer	IC_50_ p53/*h*DM2	IC_50_ Mcl-1/NOXA-B	IC_50_ Bcl-x_L_/BAK
**1**	12.3 ± 0.4 μM	>70 μM	No inhibition
**2**	40 ± 5 μM	>70 μM	No inhibition
**3**	42 ± 6 μM	>70 μM	No inhibition
**4**	20.3 ± 0.3 μM	>70 μM	No inhibition
**5**	20 ± 6 μM^*a*^	13 ± 4 μM^*a*^	No binding

^*a*^ *K*_d_ obtained through direct titration.

The functionalised helix mimetics **2** and **3** inhibit the p53/*h*DM2 interaction but the introduction of an additional functional group onto the non-binding face of the helix mimetic leads to a decrease in inhibitory potency. Several hypotheses for this change are immediately obvious (a) although the functionalised helix mimetic retains the ability to mimic the helical pharmacophore (Fig. 2), the addition of the triazole ring may introduce a steric clash between the mimetic and helix binding cleft on *h*DM2 (Fig. 4a) or (b) the introduction of the triazole ring in some way changes the conformational properties of the helix mimetic. Computational docking experiments were attempted in order to investigate the first hypothesis; these suggested that the additional functional group does not introduce a steric clash with the p53 binding cleft on *h*DM2 (Fig. S1†). In order to test the second hypothesis, we performed dihedral analysis on the oligomers (Fig. 4b and c). The analysis shows that in the case of functionalised trimers **2** and **3** there are additional energetic barriers to rotation about the central *N*-aryl bond. These higher energy conformations result from a steric clash between the *O*-alkyl group and carbonyl group on the adjacent aromatic ring (Fig. 4d) and may diminish the ability of the helix mimetic to adopt a conformation in which the α-helix mimicking side chains are aligned on one face. An analogous control analysis with all-methyl side chains was also performed (Fig. S2†) and the same steric clash was observed. This indicates that the observed effect is not due to the identity of the individual binding groups and is a general feature of this scaffold. The dihedral analysis thus may be considered to contradict the results obtained by molecular modelling however it should be noted that a perfect alignment of side-chains may not be a requirement for effective α-helix mimicry^31^ and, that both modelling analyses are not sufficiently high resolution to make meaningful comparative interpretation of energy differences *e.g.* between high/low energy conformers/rotamers.

**Fig. 4:**
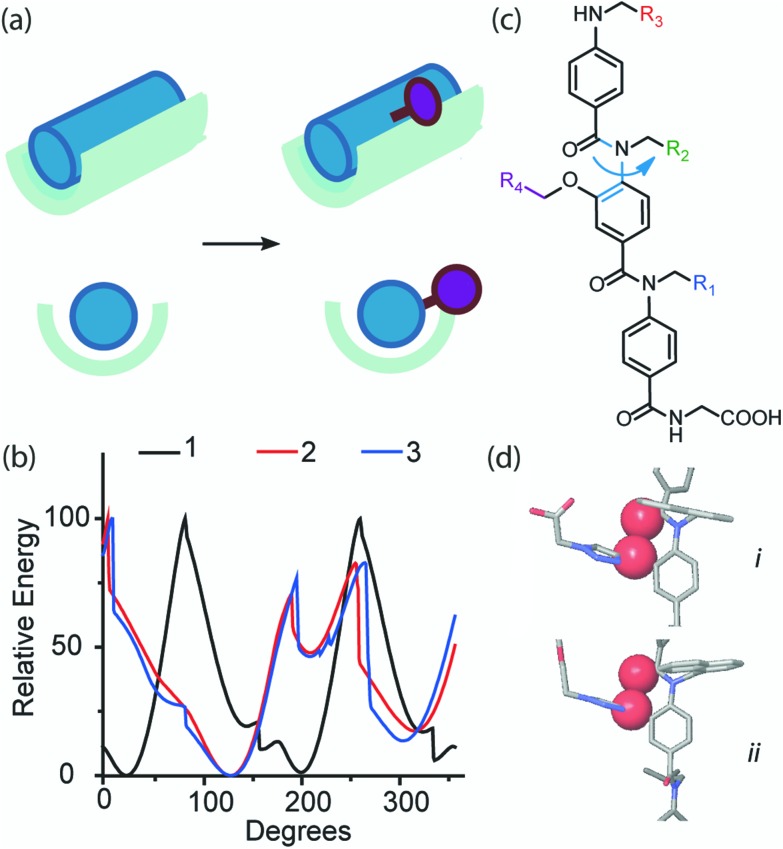
Effects of modification on helix mimicry (a) schematic illustrating potential steric clash of functional handle with helix binding cleft. (b) Dihedral angle analysis showing relative energies at different degrees of rotation about (c) the central *N*-aryl bond (d) steric clash in the functionalised trimers, between carbonyl and side chain oxygen atoms (shown in CPK format).

The selectivity of the trimer family for the p53/*h*DM2 interaction was also investigated by screening the same compounds against two additional α-helix mediated PPIs; Mcl-1/NOXA-B and Bcl-x_L_/BAK, both of which are involved in the apoptosis pathway.^45,46^ The IC_50_ values are presented in Table 1. The data shows some interesting trends in binding selectivity. The unfunctionalised scaffold **1** shows some selectivity for *h*DM2 over Mcl-1 and exhibits no inhibition of the Bcl-x_L_/BAK interaction (Fig. S7 and S8†). We were unable to fit the data to a reliable IC_50_ value for the Mcl-1/NOXA-B competition experiment as the lower limit for the anisotropy is not reached for these compounds. However, it is clear that the inhibition of Mcl-1/NOXA-B is weaker than that observed for the p53/*h*DM2 interaction for all three compounds. The moderate inhibition of Mcl-1/NOXA-B is in some respects unsurprising given that p53 itself has been shown to interact with Mcl-1.^47^ Furthermore, we note a recent publication from the Abbot group where a fragment screen and subsequent crystal structure studies illustrated that naphthyl groups are capable of opening-up a pocket in the Mcl-1 helix binding groove.^48^ The preference for Mcl-1 over Bcl-x_L_ for these trimers is also noteworthy given that several Bcl-2 family inhibitors target Bcl-x_L_ or Bcl-2^49,50^ and that selective Mcl-1 inhibitors are rarer.^51^ Most significantly, when comparing **1** with **2** and **3** the result illustrates that the selectivity profile of the helix mimetics can be effectively reproduced (even with a small decrease in inhibitory potency as a consequence of introducing the triazole handle).

The fluorescently tagged trimers **4** and **5** were designed for use in direct binding experiments. We observed that upon ‘click’ reaction, the fluorescence of the coumarin group in trimer **4** was quenched (Fig. S14†). This trimer was therefore tested in the fluorescence anisotropy competition assays as described above (Fig. S7 and S8†). The FITC-labelled trimer **5**, however, showed good fluorescence intensity (Fig. S16†). Direct binding experiments were performed using both fluorescence anisotropy (Fig. 3b) and microscale thermophoresis^52^ (Fig. S12†). From the direct binding anisotropy experiment *K*_d_ was found to be 20 ± 6 μM whereas using thermophoresis it was found to be 11 ± 2 μM. The same fluorescence anisotropy experiment was then carried out for Mcl-1 and Bcl-x_L_. The *K*_d_ for binding Mcl-1 was found to be 13 ± 4 μM whereas no binding was observed for Bcl-x_L_ (Fig. S11†). The absence of selectivity between *h*DM2 and Mcl-1 in this instance may arise as a consequence of non-specific interactions imparted by the fluorescein group – we note that *K*_d_ is lower for **5** than the IC_50_'s observed for both **2** and **3** which would support this hypothesis.

## Conclusions

In conclusion, we have described the development of versatile methodology for orthogonal functionalisation of *N*-alkylated aromatic oligoamide helix mimetics with a wide range of functional groups by exploiting alkyne–azide ‘click’ chemistry. The modifications to the non-binding face of the helix mimetic facilitate a range of additional experiments to be performed *e.g.* direct binding to target proteins to be assessed (*via* fluorescence). Although functionalisation of the non-binding face resulted in a loss of potency for trimers **2** and **3** in comparison to unfunctionalised trimer **1**, the compounds were still p53/*h*DM2 inhibitors and demonstrated selectivity towards the p53/*h*DM2 interaction over Mcl-1/NOXA-B and Bcl-x_L_/BAK; thus the binding specificity can be reproduced exactly with only a minor loss of inhibitory potency. Exploiting this methodology to add groups that promote cell-uptake, facilitate detection in cells and make favourable interactions with the solvent exposed protein surface to improve potency will be the focus of future studies by our group.
