# Supplementary material for: Orthogonal functionalisation of α-helix mimetics
Source: Org Biomol Chem. 2014 Jul 28;12(35):6794–9. doi: 10.1039/c4ob00915k (PMC4157654; doi:10.1039/c4ob00915k)
Supplement: Supplementary file 1 [file OB-012-C4OB00915K-s001.pdf]

Electronic Supplementary Material (ESI) for Organic & Biomolecular Chemistry.
This journal is © The Royal Society of Chemistry 2014

Orthogonal Functionalisation of α-Helix Mimetics

Anna Barnard,[a,b] Kérya Long,[a,b] David J. Yeo,[a,b] Jennifer Miles,[a,b] Valeria Azzarito,[a,b] George M. Burslem,[a,b] Panchami Prabhakaran,[a,b] Thomas Edwards[b] and Andrew J. Wilson*[a,b]

*A.J.Wilson@leeds.ac.uk
[a]School of Chemistry, University of Leeds, Woodhouse Lane, Leeds, LS2 9JT, UK.
[b]Astbury Centre for Structural and Molecular Biology, University of Leeds, Woodhouse Lane, Leeds, LS2 9JT, UK.

SUPPORTING INFORMATION

Contents

Experimental Procedures 2
NMR Spectra of Compounds **1-5, 7-10**, **14** and **15** 9
LC-MS and HPLC Traces of Trimers **1-5** 25
Synthesis of coumarin and fluorescein azides 28
Molecular Modelling 28
Fluorescence Anisotropy Assays 29
Thermophoresis Direct Binding Assay 37
Fluorescence and Absorbance Characterisation of Trimers **4** and **5** 37

Experimental Procedures

General Materials and Methods

All chemicals and solvents were purchased and used without further purification. ^{1}H, ^{13}C and 2D NMR Spectra were recorded with a Bruker DRX 500 MHz or DPX 300 MHz spectrometer. ^{1}H NMR spectra are referenced to tetramethylsilane (TMS) and chemical shifts are given as parts per million downfield from TMS. Coupling constants are reported to the nearest 0.1 Hz. Melting points were determined using a Griffin D5 variable temperature apparatus and are uncorrected. Microanalyses were obtained on a Carlo Erba Elemental Analyser MOD 1106 instrument. IR spectra were recorded with a Perkin–Elmer FTIR spectrometer and samples were analysed in the solid phase. Mass spectra (HRMS) were obtained with a Bruker maxis impact 3000 spectrometer using electrospray ionisation. LC-MS experiments were run on a Waters Micromass ZQ spectrometer. Analytical HPLC analysis was carried out on an Agilent Technologies 1260 Infinity on a gradient of 95-5% acetonitrile in water. Analytical TLC was performed on 0.2 mm silica gel 60 F254 precoated aluminium sheets (Merck) and visualised by using UV irradiation or, in the case of amine intermediates, by staining with ninhydrin solution. Flash chromatography was carried out on silica gel 60 (35–70 micron particles, FluoroChem). Expression of *h*DM2 and fluorescence anisotropy assays were performed as described previously.[1] Expression of Bcl-x_L and fluorescence anisotropy assays were performed as described previously.[2] The convention used to assign the spectroscopic data and for naming compounds for this series of aromatic oligoamides has been described previously.[3] Additional side chain functionalities were assigned in the NMR as 2-3° denoting the central aromatic ring functionalised at the 3-position on an oxygen atom.

Solid-Phase Trimer Synthesis

Glycine-loaded Wang resin (254 mg, 0.2 mmol) was swelled in anhydrous DMF (5 ml) 15 minutes prior to reaction. The monomers; benzyl (450 mg, 1.0 mmol), alkyne-1-naphthyl (554 mg, 1.0 mmol) and isobutyl (415 mg, 1.0 mmol) were each dissolved in anhydrous $CHCl_3$ (10 ml) and pre-activated for coupling with Ghosez's reagent (630 µl, 20% in $CHCl_3$, 0.96 mmol) for 1 hour at room temperature. The coupling reactions were carried out on a CEM Liberty microwave assisted automated peptide synthesiser. A small sample was removed and cleaved from the resin with TFA: CH_2Cl_2 (1:1, 1 ml) and analysed by LC-MS to confirm formation of the desired trimer; coupling reactions were assumed to have gone to completion.

Additional monomers used in the solid phase synthesis were synthesised as described previously.[4]

Cleavage

The cleavage step was carried out manually, in 1.5 ml 'Extract-Clean' polypropylene reservoirs fitted with 20 µm polyethylene frits (Alltech). The resin was transferred to the reservoir and washed with CH_2Cl_2 (1 ml, 5 mins) and diethyl ether (1 ml, 5 mins). A 1:1 mixture of TFA: CH_2Cl_2 was added and the mixture was stirred for 30 mins at room temperature and the contents collected and the procedure repeated. The resulting solution was concentrated affording the target compound.

Molecular Modelling

Conformational search and Superposition with the native p53 helix

A conformational search was performed on trimers **1**, **2** and **3**. The structures were minimised by employing a full *Monte Carlo* search in the software Macromodel® using the MMFFs (Merk Molecular Force Fields) method. Water was chosen as implicit solvent and rotation around the amide bonds was restricted in order to bias the conformational search towards *trans* structures. The results revealed the lowest energy conformation was the extended structure. For poses where the side chains

lie on the same face of the molecule, these matched the position of Phe19, Trp23 and Leu26, which are located at the *i*, *i* + 4, *i* + 7 positions of the p53 helix respectively.
All the conformations within 1.5 kJ/mol from the lowest energy conformation were superimposed with the p53 α-helix and a mean value of the RMSD (Root Mean Square Deviation) resulting from the superimposition of the nitrogen of the helix mimetic scaffold and the α carbon of the key amino acids of the p53 helix was calculated for conformers with matched side chain orientation.

Dihedral Angle Analysis
The energy of the lowest energy conformer determined in the conformational search as the central *N*-aryl bond was rotated was calculated in the software Macromodel® using the MMFFs (Merk Molecular Force Fields) method. Water was chosen as implicit solvent. The data was plotted as relative energy calculated from the maximum and minimum energy.

Docking
The protein structure for *h*DM2 (PDB ID: 1ycr) was prepared using the protein preparation wizard within Maestro (Schrodinger) and the docking grid prepared by selecting the binding groove of the p53 helix using Glide (Schrodinger).
Conformer libraries of compounds were generated using Omega (Openeye Scientific) and prepared for docking using Ligprep (Schrodinger). Docking was performed using Glide (Schrodinger) allowing flexible ligands but penalising non-planar amide bonds.

Fluorescence Anisotropy Direct Binding
Fluorescence anisotropy assays were performed in 384-well plates (Greiner Bio-one). Each experiment was run in triplicate and the fluorescence anisotropy measured using a Perkin Elmer EnVisionTM 2103 MultiLabel plate reader with excitation at 480 nm and emission at 535 nm (5 nm bandwidth). The experiments were performed in 40 mM phosphate buffer at pH 7.5 containing 200 mM NaCl and 0.02 mg ml^{-1} bovine serum albumin (BSA).

Thermophoresis Direct Binding
The experiments were performed in 40 mM phosphate buffer at pH 7.5 containing 200 mM NaCl and 0.02 $mgml^{-1}$ bovine serum albumin (BSA). hDM2$_{17\text{-}126}$ was diluted into buffer across 16 eppendorfs from 400 µM to 12 nM. The fluorescently tagged trimer was then added giving final protein concentrations from 200 µM to 6 nM and a fixed trimer concentration of 50 nM. The themophoresis of each sample was measured using a Nano Tempter Monolith NT.115 Microscale Thermophoresis. Ligand bound was calculated from Eq. 3 using $\lambda = 1$. K_d was calculated from Eq. 4.

Methyl 4-nitro-3-(prop-2-ynyloxy)benzoate, 7
Methyl 3-hydroxy-4-nitrobenzoate (10 g, 50.7 mmol) was dissolved in anhydrous DMF (60 ml) and K_2CO_3 (35 g, 254 mmol) was added followed by propargyl bromide (10.6 ml, 71.1 mmol) and the reaction was stirred overnight at 50 °C under a nitrogen atmosphere. The solution was then poured into EtOAc (300 ml) and washed with H_2O (2 x 100 ml) and brine (2 x 100 ml). The organic phase was dried over Na_2SO_4, filtered and the filtrate evaporated affording the desired product as a pale yellow solid (11.53 g, 49 mmol, 97%). R_f = 0.74 (CH_2Cl_2). mp = 100-103°C (MeOH). ^{1}H NMR (500 MHz, $CDCl_3$) δ: 7.90 (d, ArC*H*, ^{4}J = 1.5, 1H); 7.84 (d, ArC*H*, ^{3}J = 8.5, 1H); 7.75 (dd, ArC*H*, ^{3}J = 8.5, ^{4}J = 1.5, 1H); 4.90 (app. d, CH_2C≡CH, ^{4}J = 2, 2H); 3.97 (s, CH_3, 3H); 2.61 (app. t, CH_2C≡C*H*, ^{4}J = 2, 1H). ^{13}C NMR (125 MHz, $CDCl_3$) δ: 165.0 (*C*=O); 150.1, 143.0, 134.7 (quaternary Ar*C*); 125.4, 122.5, 116.4 (ArCH); 77.5 (CH_2*C*≡CH); 76.6 (CH_2C≡*C*H); 57.4 (*C*H_2C≡CH); 52.9 (*C*H_3). HRMS: calcd. $[M+Na]^+$ ($C_{11}H_9NNaO_5$) *m/z* = 258.0373. Found $[M+Na]^+$ *m/z* = 258.0366. ν_{max} (cm^{-1}): 3257*s*,

3119*s* (C-H alkyne), 2957*s* (C-H), 2119*s* (C≡C), 1725*s* (C=O), 1517*m*, 1272*w*, 1118*s*. Anal. calcd. C 56.17, H 3.86, N 5.96. Found C 56.45, H 3.75, N 5.80.

Methyl 4-amino-3-(prop-2-ynyloxy)benzoate,

Methyl 4-nitro-3-(prop-2-ynyloxy)benzoate (819 mg, 3.48 mmol) was dissolved in EtOAc (100 ml) and $SnCl_2.2H_2O$ (3.93 g, 17.43 mmol) was added. The reaction mixture was heated overnight at 50 °C under a nitrogen atmosphere and a drying tube. The solution was then poured into ice water (30 ml) and basified with $NaHCO_3$ (10 ml). The product was extracted with EtOAc (2 x 100 ml) and the combined organics were washed with NaOH (1M, 50 ml), H_2O (50 ml) and brine (50 ml). The organic phase was dried over Na_2SO_4, filtered and the filtrate evaporated affording the desired product as an orange crystalline solid (675 mg, 3.29 mmol, 95%): R_f = 0.78 (9:1, CH_2Cl_2: MeOH); mp = 173-175°C (MeOH); ^{1}H NMR (500 MHz, $CDCl_3$) δ: 7.59-7.56 (m, ArC*H*, 2H), 6.70 (d, ArC*H*, ^{3}J = 8, 1H), 4.76 (app. d, C*H*$_2$C≡CH, ^{4}J = 2, 2H), 3.86 (s, C*H*$_3$, 3H), 2.54 (app. t, CH_2C≡C*H*, ^{4}J = 2, 1H); ^{13}C NMR (125 MHz, $CDCl_3$) δ: 167.1 (*C*=O), 144.1, 141.3 (quaternary Ar*C*), 125.0, 119.6, 113.8, 113.2 (Ar*C*H, quaternary Ar*C*), 78.2 (CH_2*C*≡CH), 75.8 (CH_2C≡*C*H), 56.4 (*C*H_2C≡CH), 51.7 (*C*H_3); HRMS: calcd. $[M+H]^+$ ($C_{11}H_{12}NO_3$) *m/z* = 206.0812; found $[M+H]^+$ *m/z* = 206.0815; ν_{max} (cm^{-1}): 3478*s* (N-H), 3364*s*, 3242*s* (C-H alkyne), 2953*s* (C-H), 2115*s* (C≡C), 1694*s* (C=O), 1621*s*, 1519*s*, 1445*s*, 1365*w*, 1258*m*, 1188*m*, 1148*m*, 1112*s*, 1026*s*; anal. calcd. C 64.38, H 5.40, N 6.83; found C 63.90, H 5.35, N 6.45.

4-amino-3-(prop-2-ynyloxy)benzoic acid, 8

Methyl 4-amino-3-(prop-2-ynyloxy)benzoate (7.03 g, 34.3 mmol) was dissolved in MeOH: THF (1:1, 400 ml) and NaOH (100 ml, 2M) was added. The reaction mixture was stirred overnight at room temperature before being acidified with 1M HCl. The volatile solvents were removed *in vacuo* and the product extracted with EtOAc (2 x 100 ml). The combined organics were washed with HCl (1M, 50 ml), H_2O (50 ml) and brine (50 ml). The organic phase was dried over Na_2SO_4, filtered and the filtrate evaporated affording the product as a pale orange solid (5.63 g, 28.1 mmol, 82%): R_f = 0.47 (9:1, CH_2Cl_2: MeOH); ^{1}H NMR (300 MHz, MeOD) δ: 7.57 (d, ArC*H*, ^{4}J = 1.5, 1H), 7.51 (dd, ArC*H*, ^{3}J = 8, ^{4}J = 1.5, 1H), 6.71 (d, ArC*H*, ^{3}J = 8, 1H), 4.79 (app. d, ^{4}J = 2.4, C*H*$_2$C≡CH, 2H), 2.98 (app. t, ^{4}J = 2.4, CH_2C≡C*H*, 1H); ^{13}C NMR (75 MHz, MeOD) δ: 170.5 (*C*=O), 145.2, 144.6 (quaternary Ar*C*), 126.4, 119.3, 114.7, 114.3 (Ar*C*H, quaternary Ar*C*), 79.7 (CH_2*C*≡CH), 77.1 (CH_2C≡*C*H), 57.1 (*C*H_2C≡CH); HRMS: calcd. $[M+H]^+$ ($C_{10}H_{10}NO_3$) *m/z* = 192.0655; found $[M+H]^+$ *m/z* = 192.0664; ν_{max} (cm^{-1}): 3485*s* (N-H), 3382*s*, 3237*s* (C-H alkyne), 2515*s* (C-H), 2116*s* (C≡C), 1659*s* (C=O), 1607*s*, 1520*s*, 1454*s*, 1258*s*, 1213*s*, 1147*m*, 1112*s*, 1037*s*.

4-(naphthalene-1-ylmethylamino)-3-(prop-2-ynyloxy)benzoic acid, 9

4-amino-3-(prop-2-ynyloxy)benzoic acid (1 g, 5.20 mmol) was dissolved in MeOH (50 ml) and 1-naphthaldehyde (710 µl, 5.20 mmol) was added followed by 2-picoline borane complex (730 mg, 6.80 mmol). The reaction mixture was stirred overnight at room temperature. A white precipitate formed which was isolated by filtration and found to be the desired product (937 mg, 1.69 mmol, 33%): R_f = 0.51 (9:1, CH_2Cl_2: MeOH); mp = 198-201°C (MeOH); ^{1}H NMR (500 MHz, DMSO-d$_6$) δ: 12.16 (s, O*H*, 1H), 8.20 (d, ArC*H*, ^{3}J = 8, 1H), 7.97 (d, ArC*H*, ^{3}J = 8, 1H), 7.84 (d, ArC*H*, ^{3}J = 8, 1H), 7.62-7.55 (m, C*H* naph, 2H), 7.50-7.41 (m, C*H* naph, 4H), 6.50 (d, ArC*H*, ^{3}J = 8, 1H), 6.35 (t, N*H*, ^{3}J = 5.5, 1H), 4.92-4.89 (m, C*H*$_2$naph, C*H*$_2$C≡CH, 4H), 3.65 (s, CH_2C≡C*H*, 1H); ^{13}C NMR (75 MHz, DMSO-d$_6$) δ: 167.4 (*C*=O), 143.2, 142.5 (quaternary Ar*C*), 134.1, 133.4, (quaternary *C* naph), 130.8, 128.6, 127.2, 126.1, 125.8, 125.4, 124.8, 124.0, 123.3, 116.8, 112.1, 108.5 (Ar*C*H, quaternary Ar*C*, *C*H naph), 79.3 (CH_2*C*≡CH), 78.6 (CH_2C≡*C*H), 55.9 (*C*H_2C≡CH), 43.7 (*C*H_2 naph); HRMS: calcd. $[M+H]^+$ ($C_{21}H_{18}NO_3$) *m/z* = 332.128120; found $[M+H]^+$ *m/z* = 332.128118; ν_{max} (cm^{-1}): 3427*s*, 3299*s*

(O-H,N-H); 2130*w* (C≡C), 1662*s* (C=O), 1600*s*, 1578*s*, 1544*s*, 1510*w*, 1473*w*, 1440*m*,1417*w*, 1373*m*, 1314*w*, 1286*s*, 1243*w*, 1210*m*, 1157*w*, 1137*s*, 1121*w*, 1094*w*, 1036*s*.

4-((((9*H*-fluoren-9-yl)methoxy)carbonyl)(naphthalene-1-ylmethyl)amino)-3-(prop-2-ynyloxy)benzoic acid, 10

To a refluxing solution of 4-(naphthalene-2-ylmethylamino)-3-(prop-2-ynyloxy)benzoic acid (897 mg, 2.71 mmol) in chloroform (50 ml) was added dropwise a solution of Fmoc-chloride (1.19 g, 4.61 mmol) in chloroform (50 ml) and the reaction mixture was stirred overnight at reflux. The solution was cooled and filtered to remove any unreacted starting material. The solvent was then removed and the residue taken up in chloroform and precipitated with hexane affording the desired product as an off-white foam (623 mg, 1.13 mmol, 42%): R_f = 0.40 (95:5 $CHCl_3$: MeOH); mp = 164-168°C (MeOH); ^{1}H NMR (500 MHz, $CDCl_3$) δ: 8.20 (s, ArC*H*, 1H), 7.84 (d, ArC*H*, ^{3}J = 8.5, 1H), 7.73 (t, ArC*H*, ^{3}J = 8, 2H), 7.65 (d, ArC*H*, ^{3}J = 7, 2H), 7.55-7.47 (m, ArC*H*, 3H), 7.33 (s, ArC*H*, 2H), 7.25 (m, ArC*H*, 1H), 7.14 (br s, ArC*H*, 5H), 6.91 (s, ArC*H*, 1H), 5.73-4.96 (m, CH_2naph, 2H), 4.52-4.48 (m, CH_2C≡CH, CH_2 Fmoc, 4H), 4.07 (s, C*H* Fmoc, 1H), 2.43 (s, CH_2C≡C*H*, 1H); ^{13}C NMR (125 MHz, $CDCl_3$) δ: 171.1 (*C*=O), 155.4 (*C*=O), 153.3, 143.7, 141.2, 134.7, 133.6, 132.3, (quaternary Ar*C*), 130.4, 129.2, 128.6, 127.5, 126.8, 128.1, 128.0, 126.4, 125.7, 124.9, 124.0, 123.3, 119.8, 114.3, 114.2 (ArCH, quaternary Ar*C*), 77.6 (CH_2*C*≡CH), 76.3 (CH_2C≡*C*H), 67.8 (*C*H_2Fmoc), 56.0, 50.5 (*C*H_2C≡CH, *C*H_2 naph), 47.0 (*C*H Fmoc); HRMS: calcd. $[M+H]^+$ ($C_{36}H_{28}NO_5$) *m/z* = 554.196199; found $[M+H]^+$ *m/z* = 554.196735; ν_{max} (cm^{-1}): 3292*s* (N-H), 3066*s* (O-H), 2651*m* (C-H), 2123*m* (C≡C), 1694*s* (C=O), 1605*s*, 1581*m*, 1509*s*, 1450*s*, 1407*s*, 1339*w*, 1282*s*, 1197*w*, 1121*m*, 1026*s*; anal. calcd. C 78.10, H 4.92, N 2.53; found C 77.80, H 4.90, N 2.40.

***N*-(*N*-(*N*-(Benzyl-4-aminobenzoyl)-*N*-naphth-1-yl-4-aminobenzoyl)-*N*-isobutyl-4-aminobenzoyl)-glycine, 1**

Synthesised using solid phase methodology described above. The desired trimer was isolated as a sticky orange solid (71 mg, 0.099 mmol): ^{1}H NMR (300 MHz, MeOD) δ: 8.17 (d, ArC*H*, ^{3}J = 7.7, 1H), 7.85 (dd, ArC*H*, ^{3}J = 7.2, ^{4}J = 1.9, 1H), 7.75 (d, ArC*H*, ^{3}J = 8.2, 1H), 7.67 (d, 1-*H2*, ^{3}J = 8.5, 2H), 7.45-7.55 (m, ArC*H*, 2H), 7.33-7.35 (m, ArC*H*, 4H), 7.25-7.31 (m, ArC*H*, 2H), 7.16 (d, ArC*H*, ^{3}J = 7.0, 1H), 7.11 (d, ArC*H*, ^{3}J = 8.6, 2H), 7.05 (d, ArC*H*, ^{3}J = 8.6, 2H), 6.97 (d, ArC*H*, ^{3}J = 8.5, 2H), 6.67 (d, 3-*H2*, ^{3}J = 8.5, 2H), 6.66 (d, 2-*H3*, ^{3}J = 8.8, 2H), 5.55 (s, 4-*Hα*, 2H), 4.40 (s, 2-*Hα*, 2H), 4.10 (m, 1-*Hα*, 2H), 3.75 (d, 3-*Hα*, ^{3}J = 7.6, 2H), 1.77 (m, 3-*Hβ*, 1H), 0.89 (d, 3-*Hγ*, ^{3}J = 6.6, 6H); ^{13}C NMR (125 MHz, DMSO-d_6) δ: 171.2, 168.9, 168.8, 165.7, 150.1, 144.6, 139.4, 133.4, 133.1, 132.7, 131.1, 130.7, 130.5, 128.6, 128.4 128.2, 127.9, 127.5, 127.0, 126.9, 126.6, 126.2, 126.1, 125.8, 125.6, 125.2, 123.2, 121.5, 110.5, 55.7, 51.6, 50.1, 45.9, 41.1, 35.9, 26.5, 19.9; HRMS: calcd. $[M-H]^-$ ($C_{45}H_{41}N_4O_5$) *m/z* = 717.3082; found $[M-H]^-$ *m/z* = 717.3080.

Propionic acid- *N*-(*N*-(*N*-(Benzyl-4-aminobenzoyl)-*N*-naphth-1-yl-4-aminobenzoyl)-*N*-isobutyl-4-aminobenzoyl)-glycine, 2

The trimer on glycine-loaded Wang resin (74.3 mg, 0.035 mmol) was suspended in THF: H_2O (1:1, 1 ml) and methyl 2-azidoacetate (3.41 µl, 0.036 mmol) was added followed by $CuSO_4.5H_2O$ (0.88 mg, 3.5 x 10^{-3} mmol) and sodium ascorbate (1.39 mg, 7 x 10^{-3} mmol). The suspension was the mixed overnight on a spinner at room temperature. The solution was then drained and the resin washed sequentially with H_2O, CH_2Cl_2 and Et_2O. The product was then cleaved from the resin TFA: CH_2Cl_2 (1:1, 1 ml). The solvent was then removed and the residue taken up in MeOH:THF (1:1, 2ml) and NaOH was added (2M, 0.5 ml) and the reaction mixture was stirred at room temperature for 1 hour. The volatile solvents were then removed and the aqueous solvent acidified. The product was extracted with EtOAc (50 ml) and washed with HCl (1M, 50 ml) and brine (50 ml). The organic phase was then

dried over Na_2SO_4, filtered and the filtrate evaporated affording the desired trimer as a sticky orange solid (25.4 mg, 0.029 mmol, 83%): R_f = 0.14 (3:2 CH_2Cl_2: MeOH); ^{1}H NMR (500 MHz, DMSO-d$_6$) δ: 8.86-8.84 (m, 4-N*H*, 1H), 8.14-8.12 (m, 2-3°-*T*, 1H), 7.99 (s, ArC*H*, 1H), 7.87-7.72 (m, ArC*H*, 4H), 7.49-7.45 (m, ArC*H*, 2H), 7.30-7.06 (m, ArC*H*, 10H), 6.89 (d, 1-*H2*, ^{3}J = 8, 2H), 6.38-6.25 (m, 2-*H5*, 2-*H6*, 1-*H3*, 4H), 5.33 (s, 2-3°-*Hβ*, 2H), 4.88 (s, 1-N*H,* 1H), 4.22-4.19 (m, 2-*Hα*, 2-3°-*Hα*, 1-*Hα*, 6H), 3.95 (d, 4-*Hα*, ^{3}J = 6, 2H), 3.66 (s, 3-*Hα*, 2H), 1.70-1.68 (m, 3-*Hβ*, 1H), 0.84 (d, 3-*Hγ*, ^{3}J = 6, 6H); ^{13}C NMR (125 MHz, DMSO-d$_6$) δ: 171.3, 169.6, 168.7, 168.4, 165.8, 152.4, 149.8, 142.2, 139.5, 135.9, 134.5, 133.0, 132.6, 132.5, 131.1, 129.6, 129.4, 129.1, 128.4, 128.2, 128.0, 127.7, 127.3, 127.2, 127.0, 126.7, 126.6, 126.1, 125.5, 125.5, 125.1, 123.5, 122.4, 110.5, 50.5, 48.4, 46.0, 41.2, 40.0, 39.7, 26.5, 19.9; HRMS: calcd. $[M+H]^+$ ($C_{50}H_{48}N_7O_8$) *m/z* = 874.355888; found $[M+H]^+$ *m/z* = 874.356589; ν_{max} (cm^{-1}): 3345*s* (O-H), 2926*s* (C-H), 1729*s* (C=O), 1701*s* (C=O), 1653*s*, 1635*s*, 1603*s*, 1554*w*, 1505*s*, 1427*m*, 1161*s*, 1020*m*.

Tetraethylene glycol-*N*-(*N*-(*N*-(Benzyl-4-aminobenzoyl)-*N*-naphth-1-yl-4-aminobenzoyl)-*N*-isobutyl-4-aminobenzoyl)-glycine, 3

The trimer on glycine-loaded Wang resin (63.5 mg, 0.03 mmol) was suspended in THF: H_2O (1:1, 1 ml) and azido-tetraethylene glycol monomethyl ether (7 mg, 0.03 mmol) was added followed by $CuSO_4.5H_2O$ (0.75 mg, 3 x 10^{-3} mmol) and sodium ascorbate (0.59 mg, 6 x 10^{-3} mmol). The suspension was the mixed overnight on a spinner at room temperature. The solution was then drained and the resin washed sequentially with H_2O, CH_2Cl_2 and Et_2O. The product was then cleaved from the resin TFA: CH_2Cl_2 (1:1, 1 ml). The solvent was then removed and the residue taken up in MeOH:THF (1:1, 2ml) and NaOH was added (2M, 0.5 ml) and the reaction mixture was stirred at room temperature for 1 hour. The volatile solvents were then removed and the aqueous solvent acidified. The product was extracted with EtOAc (50 ml) and washed with HCl (1M, 50 ml) and brine (50 ml). The organic phase was then dried over Na_2SO_4, filtered and the filtrate evaporated affording the desired trimer as a sticky orange solid (22.3 mg, 0.02 mmol, 75%): R_f = 0.21 (3:2, CH_2Cl_2: MeOH);
^{1}H NMR (500 MHz, DMSO-d$_6$) δ: 8.87-8.84 (m, 4-N*H*, 1H), 8.13 (s, 2-3°-*T*, 1H), 7.92 (s, ArC*H*, 1H), 7.86-7.84 (m, ArC*H*, 1H), 7.75-7.72 (m, ArC*H*, 3H), 7.48-7.47 (m, ArC*H*, 3H), 7.30-7.28 (m, ArC*H*, 5H), 7.25-7.20 (m, ArC*H*, 2H), 7.12-7.03 (m, ArC*H,* 4H), 6.88 (d, 1-*H2*, ^{3}J = 9, 2H), 6.38-6.26 (m, 2-*H6*, 2-*H5*, 1-*H3*, 4H), 4.89 (s, 1-N*H*, 1H), 4.55 (t, 2-3°-*Hβ*, ^{3}J = 4.5, 2H), 4.23 (s, 1-*Hα* 2-*Hα*, 4H), 3.95 (d, 4-*Hα*, ^{3}J = 6, 2H), 3.83 (t, 2-3°-*Hγ*, ^{3}J = 5, 2H), 3.66 (s, 3-*Hα*, 2H), 3.53-3.52 (m, 2-3°-*Hθ*, 2H), 3.50-3.45(m, 2-3°-*Hδ*, 2-3°-*Hε*, 2-3°-*Hζ*, 2-3°-*Hη*, 8H), 3.39-3.37 (m, 2-3°-*Hι*, 2H), 1.71-1.66 (m, 3-*Hβ*, 1H), 0.86-0.82 (m, 3-*Hγ*, 6H); ^{13}C NMR (125 MHz, DMSO-d$_6$) δ: 171.2, 168.7, 165.8, 158.3, 158.0, 152.4, 149.7, 142.1, 139.5, 133.0, 132.7, 131.2, 131.1, 129.6, 129.4, 129.1, 128.4, 128.2, 128.0, 127.7, 127.2, 127.0, 126.6, 126.0, 125.5, 125.1, 124.4, 123.5, 122.4, 110.5, 72.2, 69.7, 69.6, 69.5, 68.7, 60.1, 49.5, 46.0, 41.2, 26.5, 19.9; HRMS: calcd. $[M+H]^+$ ($C_{56}H_{62}N_7O_{10}$) *m/z* = 992.455268; found $[M+H]^+$ *m/z* = 992.456340; ν_{max} (cm^{-1}): 3357*s* (O-H), 2958*s* (C-H), 1736*s* (C=O), 1635*s*, 1604*s*, 1572*w*, 1506*s*, 1421*m*, 1316*w*, 1177*s*.

***tert*-Butyl 3-bromopropylcarbamate, 11[5]**

To a cooled and stirred suspension of 3-bromopropylamine (18 g, 83 mmol) and di-*tert*-butyl dicarbonate (18 g, 83 mmol) in CH_2Cl_2 (40 ml), triethylamine (18 ml, 165 mmol) was added dropwise over 1 h and the reaction mixture was stirred overnight at room temperature. The solution was diluted with CH_2Cl_2 (15 ml) and washed with HCl (2 x 20 ml, 1 M) and H_2O (2 x 20 ml). The organic phase was dried over Na_2SO_4, filtered and the filtrate evaporated *in vacuo* affording the product as a yellow oil (18.9 g, 80 mmol, 96%): R_f = 0.80 (9:1 CH_2Cl_2: MeOH); ^{1}H NMR (500 MHz, $CDCl_3$) δ: 4.79 (s, N*H*, 1H), 3.39 (t, C*H$_2$*Br, ^{3}J = 6.3, 2H), 3.21 (t, C*H$_2$*NH, ^{3}J = 6.3, 2H), 2.00 (quintet, C*H$_2$*, ^{3}J = 6.3,

2H), 1.39 (s, C(C*H*$_3$)$_3$), 9H); ^{13}C NMR (125 MHz, $CDCl_3$) δ: 155.9 (*C*ONH), 79.2 (*C*(CH$_3$)$_3$), 38.9 (*C*H$_2$NH), 32.6 (*C*H$_2$Br), 30.7 (*C*H$_2$); 28.3 (C(*C*H$_3$)$_3$); HRMS: calcd. [M+Na]$^+$ ($C_8H_{13}{}^{79}BrNNaO_2$) *m/z* = 260.0257; found [M+Na]$^+$ *m/z* = 260.0250; ν_{max} (cm^{-1}): 3355*s* (N-H); 2976*s* (C-H); 1694*s* (C=O); 1519*m*, 1251*m*, 1165*m*, 1076*m*, 1043*m*, 985*w*, 958*w*, 871*m*, 780*m*, 645*m*, 566*m*.

Butyl 3-azidopropylcarbamate, 12[5]

tert-Butyl 3-bromopropylcarbamate (16.7 g, 70 mmol) and sodium azide (9.1 g, 140 mmol) were dissolved in H_2O/Dioxane (1:1, 100 ml). The reaction was heated to reflux at 75 °C for 2 h and then stirred overnight at room temperature. The reaction was diluted with CH_2Cl_2 (80 ml) and washed with H_2O (3 x 60ml). The organic layer was then dried over Na_2SO_4, filtered and the filtrate evaporated affording the product as a pale yellow oil (10.62 g, 53.1 mmol, 76%): R_f = 0.77 (9:1 CH_2Cl_2: MeOH); ^{1}H NMR (500 MHz, $CDCl_3$) δ: 4.77 (br s, N*H*, 1H), 3.31 (t, C*H$_2$*N$_3$, ^{3}J = 6.75, 2H), 3.16 (t, C*H$_2$*NH, ^{3}J = 6.8, 2H), 1.73 (app. quintet, C*H$_2$*, ^{3}J = 6.8, 2H), 1.40 (s, C(C*H$_3$*)$_3$, 9H); ^{13}C NMR (125 MHz, $CDCl_3$) δ: 155.9 (*C*=O), 79.2 (*C*(CH$_3$)$_3$), 49.0 (*C*H$_2$N$_3$), 37.9 (*C*H$_2$NH), 29.2 (*C*H$_2$), 28.3 (C(*C*H$_3$)$_3$); HRMS: calcd. [M+Na]$^+$ ($C_8H_{16}N_4NaO_2$) *m/z* = 223.1165; found [M+Na]$^+$ *m/z* = 223.1160; ν_{max} (cm^{-1}): 3354*m* (N-H), 2978*s* (C-H), 2098*s* (N$_3$), 1694*s* (C=O), 1524*s*, 1455*w*, 1392*w*, 1366*m*, 1253*s*, 1172*s*, 1040*w*, 1008*w*, 972*w*, 873*m*, 781*w*, 633*w*, 557*w*.

3-azidopropan-1-amine, 13[5]

tert-Butyl 3-azidopropylcarbamate (1 g, 5 mmol) was dissolved in methanolic HCl (20 ml, 2M). The reaction mixture was stirred for 2 hours at room temperature and then concentrated *in vacuo*. The product was isolated as a white solid (660 mg, 4.85 mmol, 97%): ^{1}H NMR (500 MHz, $CDCl_3$) δ: 8.22 (s, N*H*, 3H), 3.57 (br s, C*H$_2$*N$_3$, 2H), 3.17 (br s, C*H$_2$*NH, 2H), 2.10 (br s, C*H$_2$*, 2H); ^{13}C NMR (125 MHz, $CDCl_3$) δ: 48.6 (*C*H$_2$N$_3$), 37.9 (*C*H$_2$NH), 26.8 (*C*H$_2$); HRMS: calcd. [M-Cl]$^+$ ($C_3H_9N_4$) *m/z* = 101.0822; found [M-Cl]$^+$ *m/z* = 101.0823; ν_{max} (cm^{-1}): 2928*s* (N-H), 2105*s* (N$_3$), 1614*s*, 1505*s*, 1396*m,* 1268*s*, 1159*s*, 1044*s*, 1020*s*, 993*s*, 864*s*, 774*s*, 656*m*, 560*m*, 509*m*.

***N*-(3-azidopropyl)-2-oxo-2*H*-chromene-3-carboxamide, 14**

Coumarin-3-carboxylic acid (70 mg, 0.37 mmol) and 3-azidopropan-1-amine (100 mg, 0.74 mmol) were suspended in CH_2Cl_2 (20 ml) and DIPEA (256 µl, 1.47 mmol). HBTU (279 mg, 0.74 mmol) was then added and the reaction mixture was stirred overnight at room temperature. The solution was washed with HCl (1M, 2 x 20 ml) and H_2O (20 ml) and the organic phase was dried over Na_2SO_4, filtered and the filtrate evaporated affording the crude product which was purified by column chromatography (SiO_2, 95:5 CH_2Cl_2: MeOH) affording the desired product as a yellow crystalline solid (67 mg, 0.25 mmol, 67%): R_f = 0.77 (95:5 CH_2Cl_2: MeOH); ^{1}H NMR (500 MHz, $CDCl_3$) δ: 8.87-8.85 (m, C*H*CC=O, N*H*, 2H), 7.62-7.59 (m, ArC*H*, 2H), 7.36-7.31 (m, ArC*H*, 2H), 3.51 (app. q, C*H$_2$*NH, ^{3}J = 11, 2H), 3.37 (t, C*H$_2$*N$_3$, ^{3}J = 11, 2H), 1.87 (app. quintet, C*H$_2$*, ^{3}J = 11, 2H); ^{13}C NMR (125 MHz, $CDCl_3$) δ: 161.5, 161.3 (*C*=O), 154.2 (*C*C=O), 148.2 (*C*HCC=O), 134.0, 129.7, 125.15 (Ar*C*H), 118.4, 118.1 (quaternary Ar*C*), 116.4 (Ar*C*H), 49.0 (*C*H$_2$N$_3$), 37.1 (*C*H$_2$NH), 28.6 (*C*H$_2$); HRMS: calcd. [M+Na]$^+$ ($C_{13}H_{12}N_4NaO_3$) *m/z* = 295.0802; found [M+Na]$^+$ *m/z* = 295.0807; ν_{max} (cm^{-1}): 3364*s* (N-H), 3046*s* (C-H), 2954*s* (C-H), 2094*s* (N$_3$), 1700*s* (C=O), 1652*s* (C=O), 1611*s*, 1567*s*, 1531*s*, 1448*s*, 1367*m*, 1346*s*, 1253*m*, 1213*m,* 1167*m*, 1077*s*, 998*s*, 925*m*, 901*m*, 798*m*, 766*s*, 740*w*, 719*w*, 632s, 524*m,* 458*m*; anal. calc. C 57.35, H 4.44, N 20.58; found C 57.30, H 4.65, N 20.30.

5-(3-(3-azidopropyl)thioureido)-2-(6-hydroxy-3-oxo-3*H*-xanthen-9-yl)benzoic acid, 15

Fluorescein isothiocyanate isomer I (200 mg, 0.51 mmol) was dissolved in anhydrous DMF (20 ml) and triethylamine was added followed by 3-azidopropylamine (77 mg, 0.57 mmol). The reaction mixture was stirred overnight at room temperature before being poured into EtOAc (300 ml) and

washed with HCl (1M, 2 x 200 ml) and brine (2 x 200 ml). The organic phase was dried over Na_2SO_4, filtered and the filtrate evaporated affording the product as an orange solid (200 mg, 0.42 mmol, 80%) The isolated product was considered of sufficient purity for further reaction: R_f = 0.78 (9:1, CH_2Cl_2: MeOH); ^{1}H NMR (500 MHz, DMSO-d_6) δ: 8.20 (s, C*H*CO_2H, 1H), 7.74-7.73 (m, C*H*CHNH, 1H), 7.19 (d, C*H*NH, ^{3}J = 8.5, 1H), 6.68-6.56 (m, ArC*H*, 6H), 3.59 (m, C$H_2$$N_3$, 2H), 3.44 (t, C$H_2$NH, ^{3}J = 7, 2H), 1.85 (quintet, CH_2, ^{3}J = 7, 2H); insufficient solubility limited the ability to acquire ^{13}C NMR Spectra; HRMS: calcd. $[M+H]^+$ ($C_{24}H_{20}N_5O_5S$) *m/z* = 490.117966; found $[M+H]^+$ *m/z* = 490.118861; ν_{max} (cm^{-1}): 3069*br* (O-H), 2097*s* (N_3), 1728*s* (C=O), 1584*s*, 1532*s*, 1451*s*, 1385*s*, 1172*s*, 1109*s*.

Coumarin-*N*-(*N*-(*N*-(Benzyl-4-aminobenzoyl)-*N*-naphth-1-yl-4-aminobenzoyl)-*N*-isobutyl-4-aminobenzoyl)-glycine, 4

The trimer on glycine-loaded Wang resin (51.4 mg, 0.025 mmol) was suspended in THF: H_2O (1:1, 1 ml) and *N*-(3-azidopropyl)-2-oxo-2*H*-chromene-3-carboxamide (6.8 mg, 0.025 mmol) was added followed by $CuSO_4.5H_2O$ (0.63 mg, 2.5 x 10^{-3} mmol) and sodium ascorbate (1.0 mg, 5 x 10^{-3} mmol). The suspension was then mixed overnight on a spinner at room temperature. The solution was then drained and the resin washed sequentially with H_2O, CH_2Cl_2 and Et_2O. The product was then cleaved from the resin TFA: CH_2Cl_2 (1:1, 1 ml). The solvent was then removed affording the desired trimer as a sticky orange solid (30.7 mg, 0.029 mmol, 100%): R_f = 0.55 (3:2, CH_2Cl_2: MeOH); ^{1}H NMR (500 MHz, DMSO-d_6) δ: 8.86-8.80 (m, 4-N*H*, 2-3°-N*H*, 2H), 8.12 (m, 2-3°-*T*, 1H), 7.94-7.93 (m, ArC*H*, 2H), 7.84-7.83 (m, ArC*H*, 1H), 7.76-7.71 (m, ArC*H*, 4H), 7.51-7.42 (m, ArC*H*, 4H), 7.30-7.18 (m, ArC*H*, 7H), 7.11 (d, ArC*H*, ^{3}J = 7.5, 2H), 7.04-7.02 (m, ArC*H*, 2H), 6.89 (d, 1-*H2*, ^{3}J = 8, 2H), 6.39-6.28 (m, 2-*H6*, 2-*H5*, 1-*H3*, 4H), 4.86 (s, 1-N*H*, 1H), 4.45 (s, 2-3°-*Hβ*, 2-3°-*Hα*, 4H), 4.22 (s, 1-*Hα*, 2-*Hα*, 2H), 3.95 (d, 4-*Hα*, ^{3}J = 5.5, 2H), 3.65 (s, 3-*Hα*, 2H), 3.38 (d, 2-3°-*Hδ*, ^{3}J = 6.5, 2H), 2.13-2.08 (m, 2-3°-*Hγ*, 2H), 1.69-1.63 (m, 3-*Hβ*, 2H), 0.82 (d, 3-*Hγ*, ^{3}J = 6.5, 6H); ^{13}C NMR (125 MHz, DMSO-d_6) δ: 162.1, 154.1, 147.7, 130.5, 130.0, 128.7, 127.5, 127.5, 127.5, 127.1, 125.7, 125.5, 118.6, 116.5, 111.0, 36.9, 36.3, 20.3; HRMS: calcd. $[M+H]^+$ ($C_{61}H_{57}N_8O_9$) *m/z* = 1045.424302; found $[M+H]^+$ *m/z* = 1045.424635; ν_{max} (cm^{-1}): 3318*s* (O-H), 2958*s* (C-H), 1701*s* (C=O), 1653*s*, 1601*s*, 1554*m*, 1506*s*, 1415*m*, 1162*s*.

Fluorescein-*N*-(*N*-(*N*-(Benzyl-4-aminobenzoyl)-*N*-naphth-1-yl-4-aminobenzoyl)-*N*-isobutyl-4-aminobenzoyl)-glycine, 5

The trimer on glycine-loaded Wang resin (74.8 mg, 0.036 mmol) was suspended in THF: H_2O (1:1, 1 ml) and azido-fluorescein (18 mg, 0.036 mmol) was added followed by $CuSO_4.5H_2O$ (0.9 mg, 3.6 x 10^{-3} mmol) and sodium ascorbate (1.4 mg, 7.2 x 10^{-3} mmol). The suspension was then mixed overnight on a spinner at room temperature. The solution was then drained and the resin washed sequentially with H_2O, CH_2Cl_2 and Et_2O. The product was then cleaved from the resin TFA: CH_2Cl_2 (1:1, 1 ml). The solvent was then removed affording the desired trimer as a dark orange solid (53.5 mg, 0.042 mmol, 100%): R_f = 0.71 (8:2, CH_2Cl_2: MeOH); ^{1}H NMR (500 MHz, DMSO-d_6) δ: 10.00 (s, 2-3°-N*H*, 2H), 8.85 (s, 4-N*H*, 1H), 8.15-8.12 (m, 2-3°-*T*, 1H), 7.96-7.83 (m, ArC*H*, 2H), 7.75-7.71 (m, ArC*H*, 4H), 7.46-7.45 (m, ArC*H*, 2H), 7.29-7.02 (m, ArC*H*, 9H), 6.88 (d, 1-*H2*, ^{3}J = 8, 2H), 6.69-6.54 (m, 2-3°-ArC*H*, 6H), 6.39-6.27 (m, 2-*H6*, 2-*H5*, 1-*H3*, 4H), 4.85 (s, 1-N*H*, 1H), 4.46 (s, 2-3°-*Hβ*, 2-3°-*Hα*, 4H), 4.22 (s, 1-*Hα*, 2-*Hα*, 4H), 3.95 (d, 4-*Hα*, ^{3}J = 5.5, 2H), 3.65-3.63 (m, 3-*Hα*, 2-3°-*Hδ*, 4H), 2.19-2.16 (m, 2-3°-*Hγ*, 2H), 1.68-1.63 (m, 3-*Hβ*, 2H), 0.82 (d, 3-*Hγ*, ^{3}J = 6.5, 6H); insufficient solubility limited the ability to acquire ^{13}C NMR Spectra; HRMS: calcd. $[M+H]^+$ ($C_{72}H_{64}N_9O_{11}S$) *m/z* = 1262.4441; found $[M+H]^+$ *m/z* = 1262.4469; ν_{max} (cm^{-1}): 3650*s* (O-H), 1705*s* (C=O), 1650*s*, 1595*s*, 1559*m*, 1508*s*, 1458*m*, 1200*s*.

NMR Spectra of Compounds 1-5, 7-10, 14 and 15.

^{1}H NMR Spectra of Compound **7** in $CDCl_3$

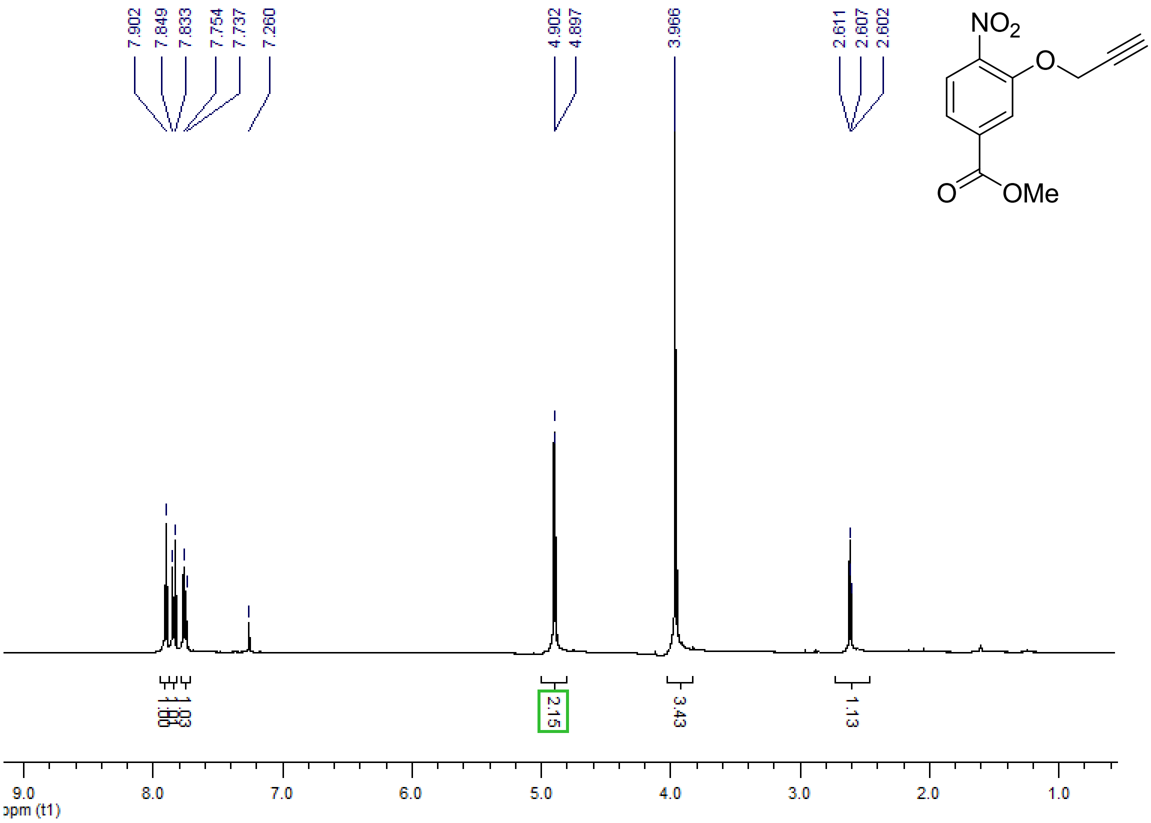

^{13}C NMR Spectra of Compound **7** in $CDCl_3$

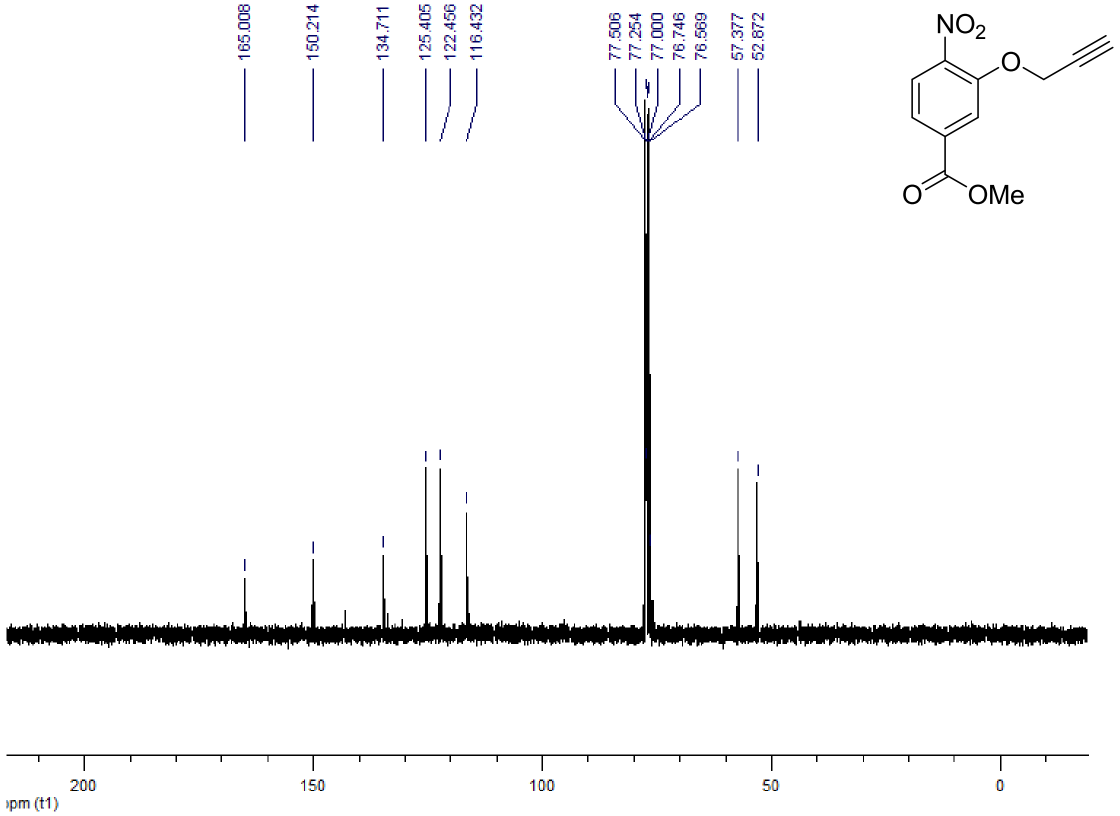

^{1}H NMR Spectra of Methyl 4-amino-3-(prop-2-ynyloxy)benzoate in $CDCl_3$

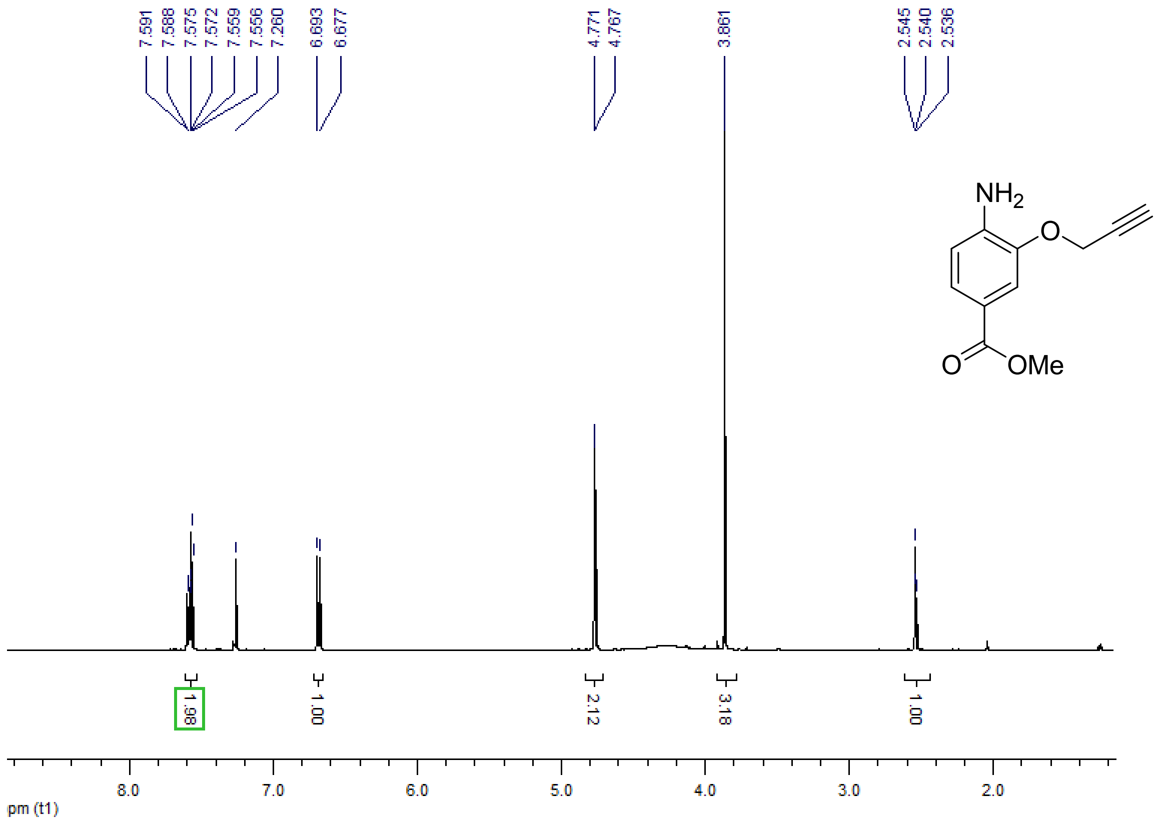

^{13}C NMR Spectra of Methyl 4-amino-3-(prop-2-ynyloxy)benzoate in $CDCl_3$

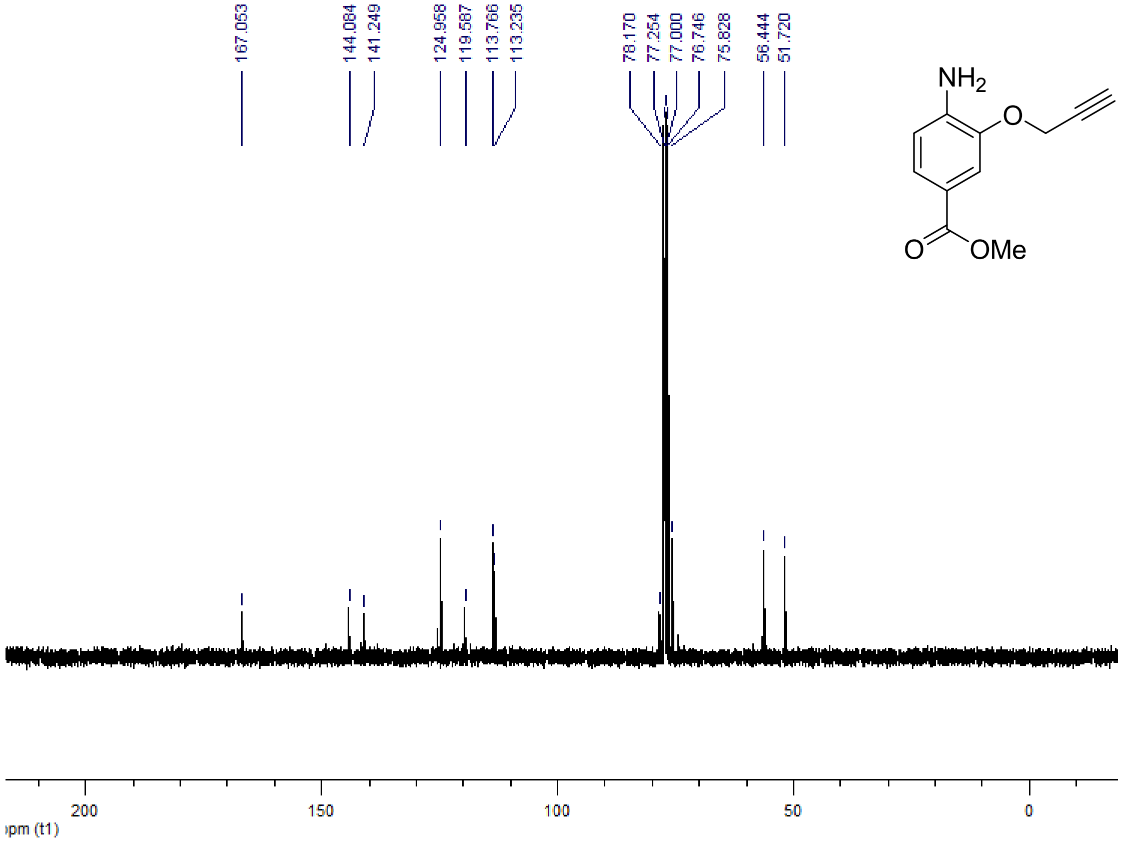

^{1}H NMR Spectra of Compound **8** in MeOD

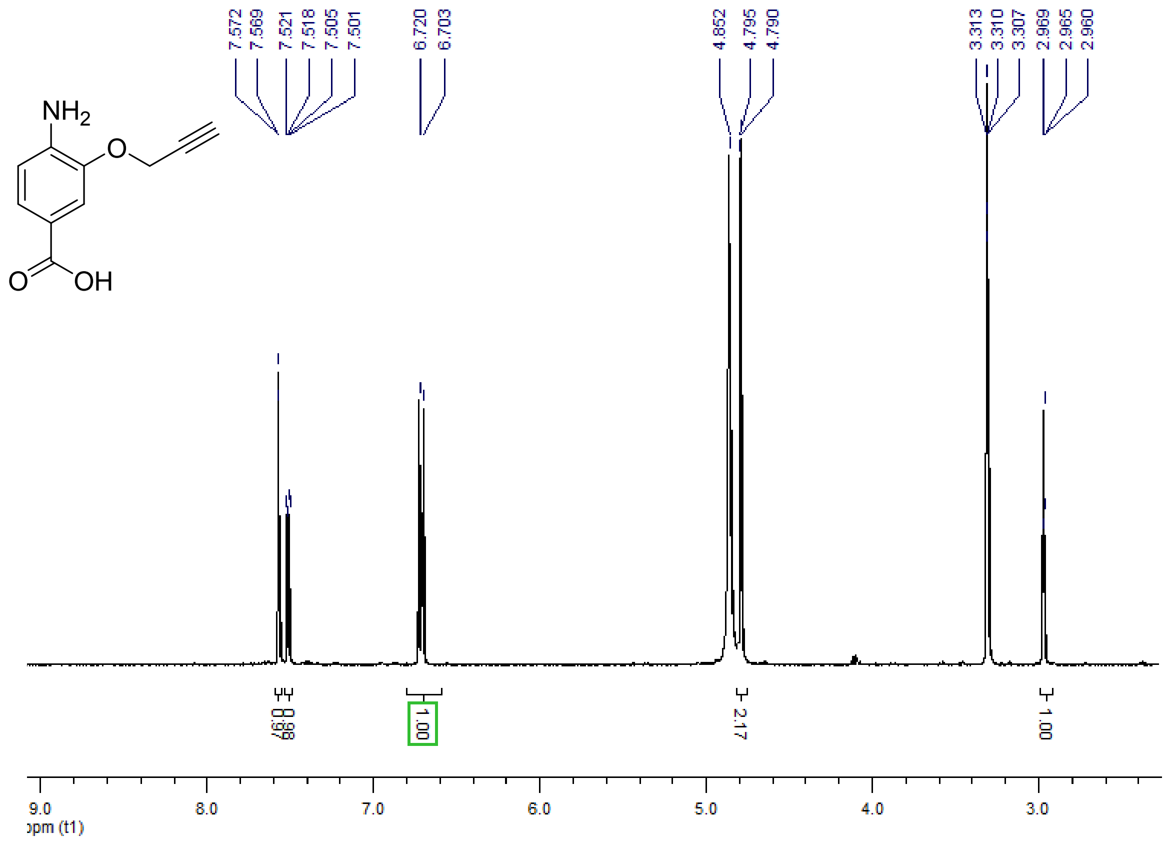

^{13}C NMR Spectra of Compound **8** in MeOD

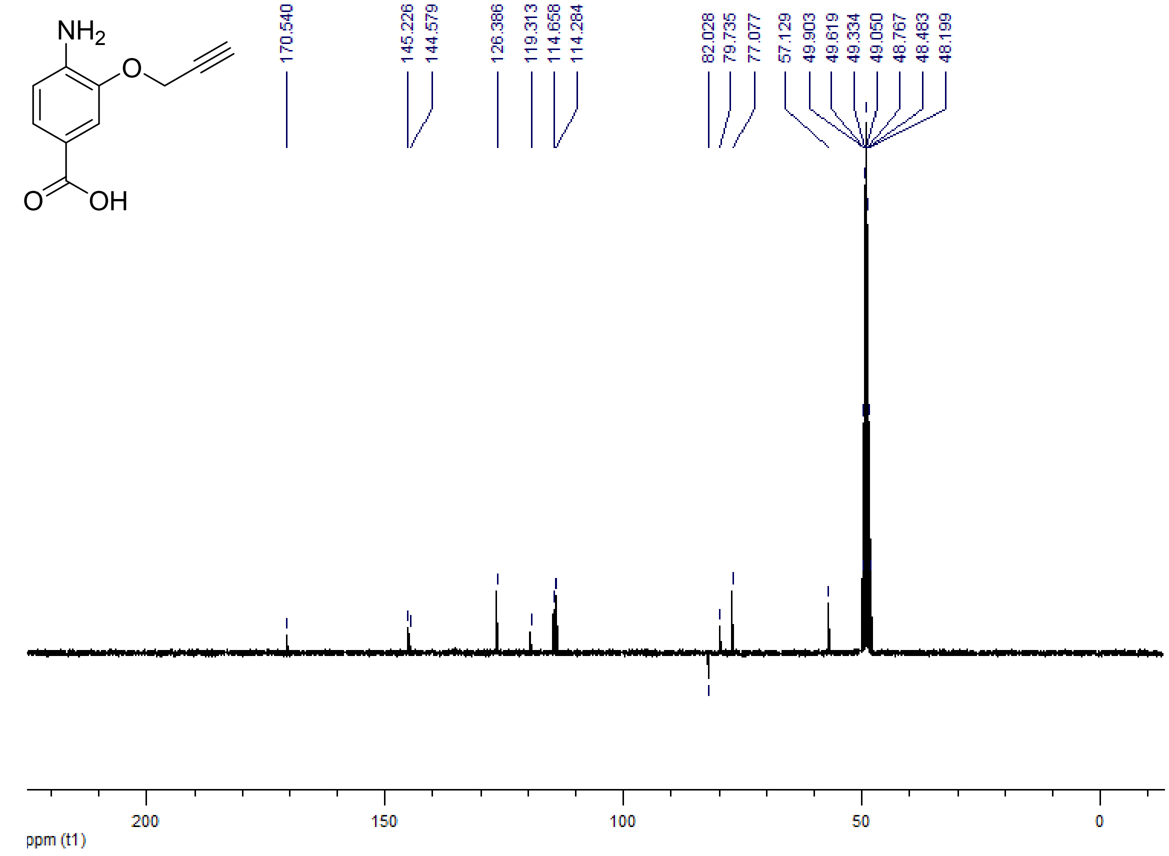

^{1}H NMR Spectra of Compound **9** in DMSO-d_6

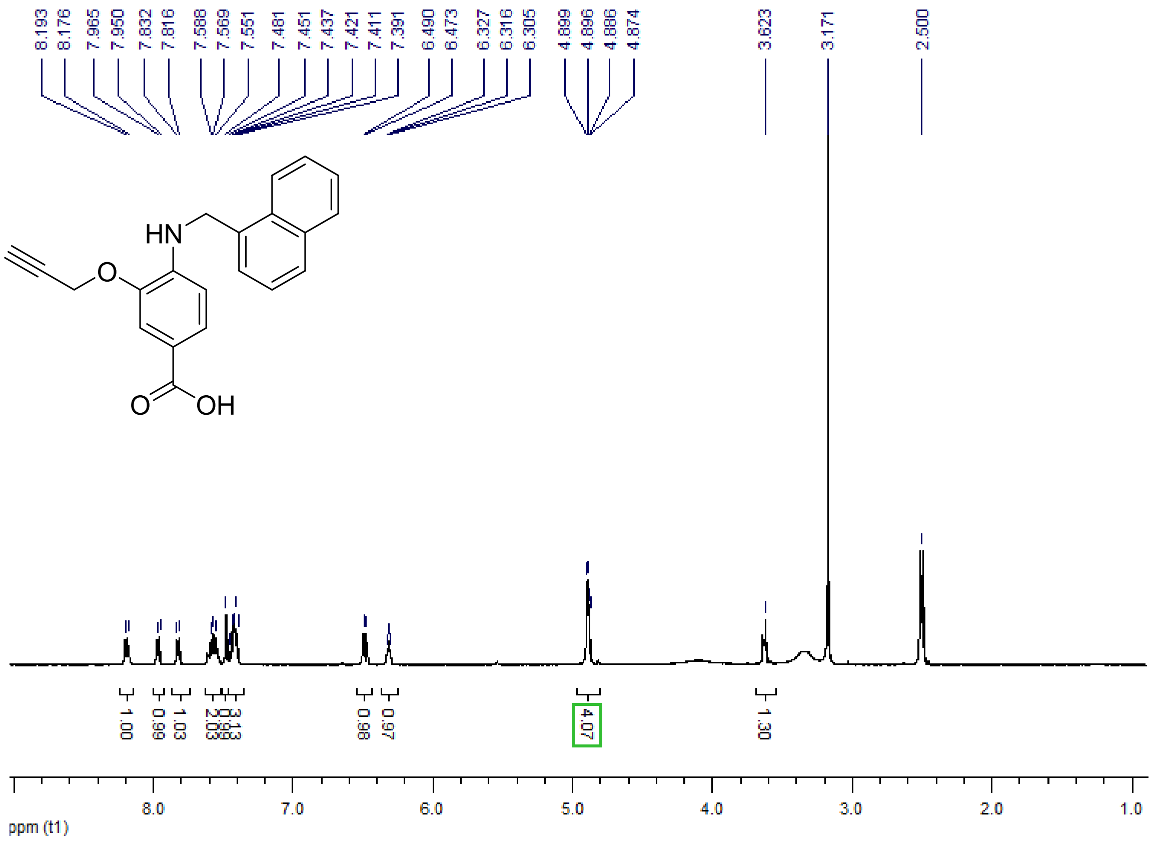

^{13}C NMR Spectra of Compound **9** in DMSO-d_6

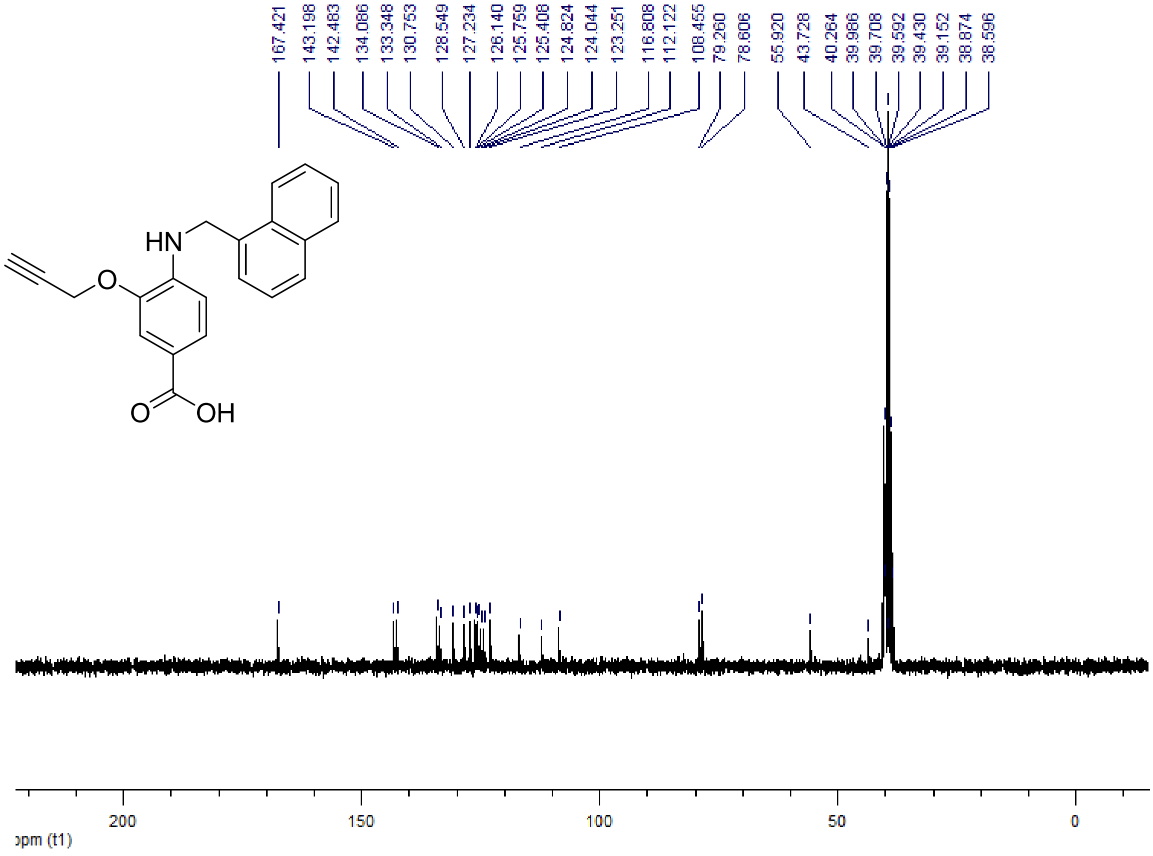

^{1}H NMR Spectra of Compound **10** in DMSO-d_6

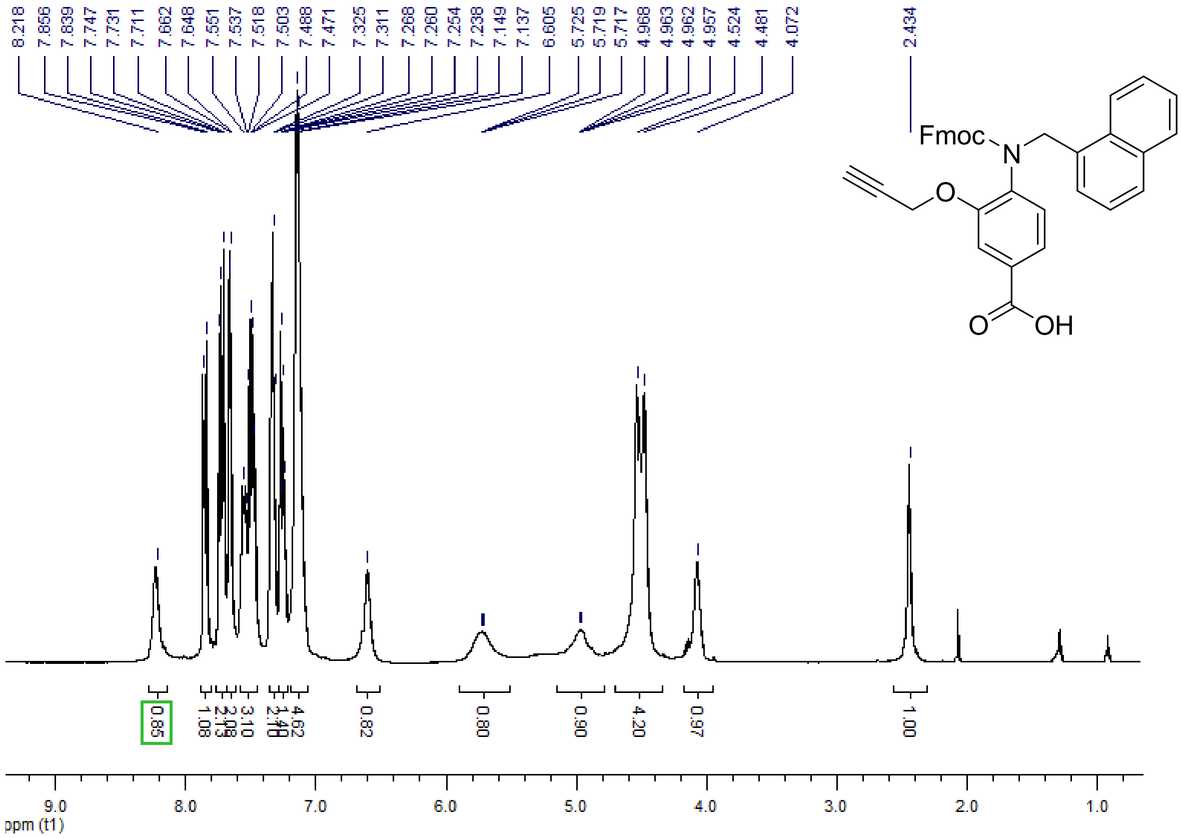

^{13}C NMR Spectra of Compound **10** in DMSO-d_6

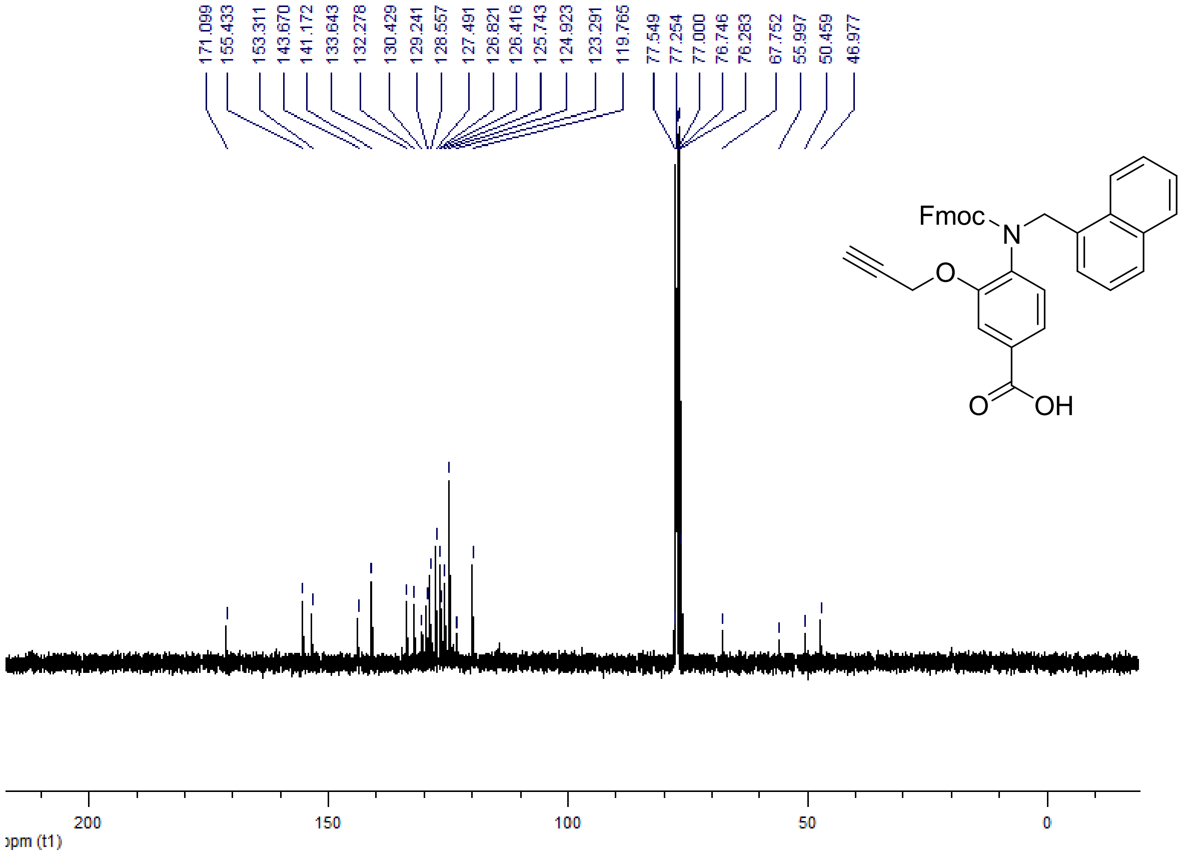

^{1}H NMR Spectra of Compound **1** in MeOD

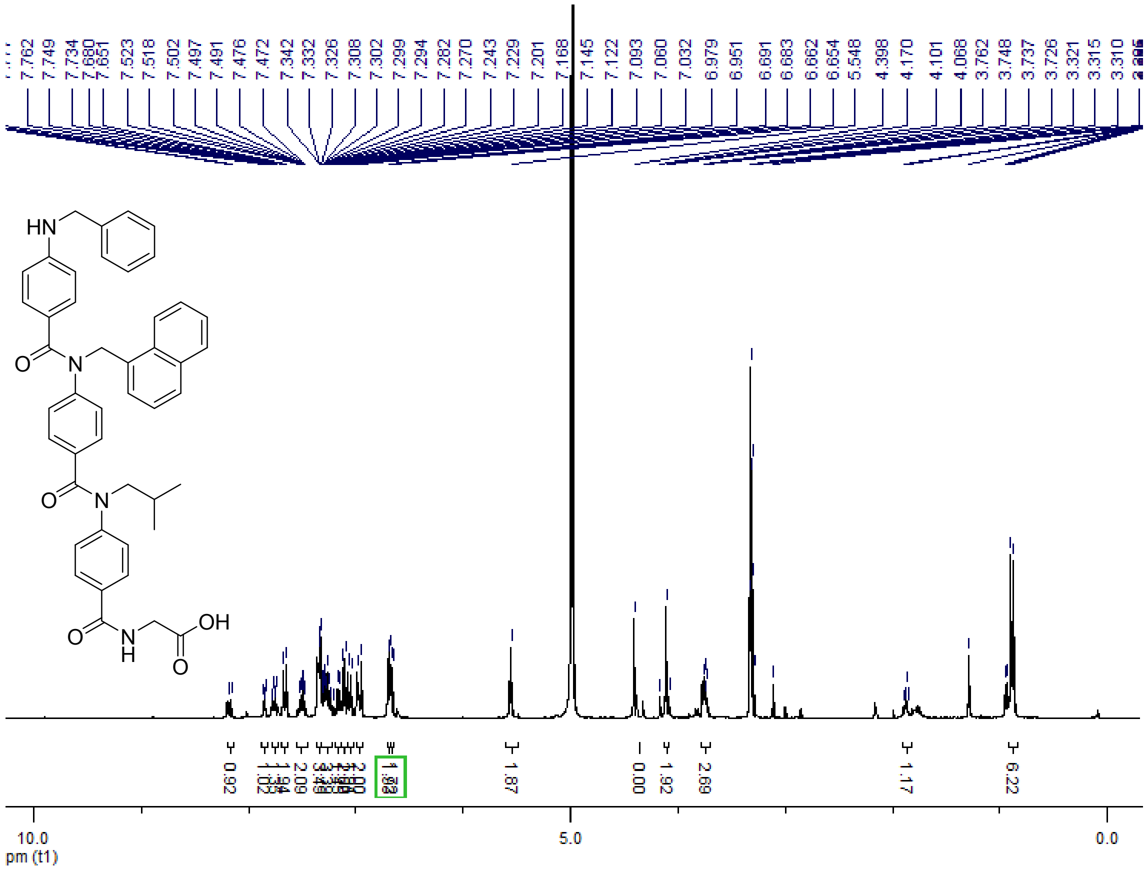

^{1}H-^{1}H COSY NMR Spectra of Compound **1** in MeOD

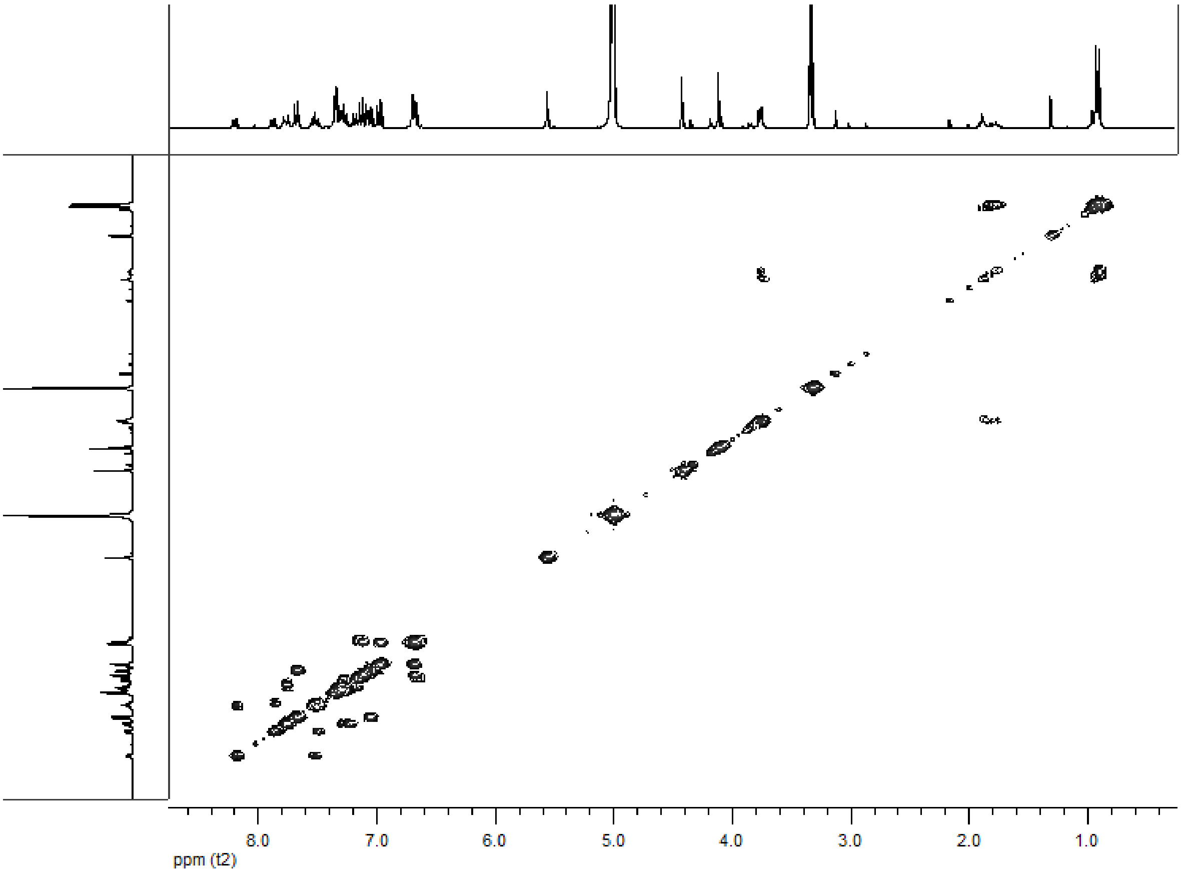

^{13}C NMR Spectra of Compound **1** in DMSO-d_6

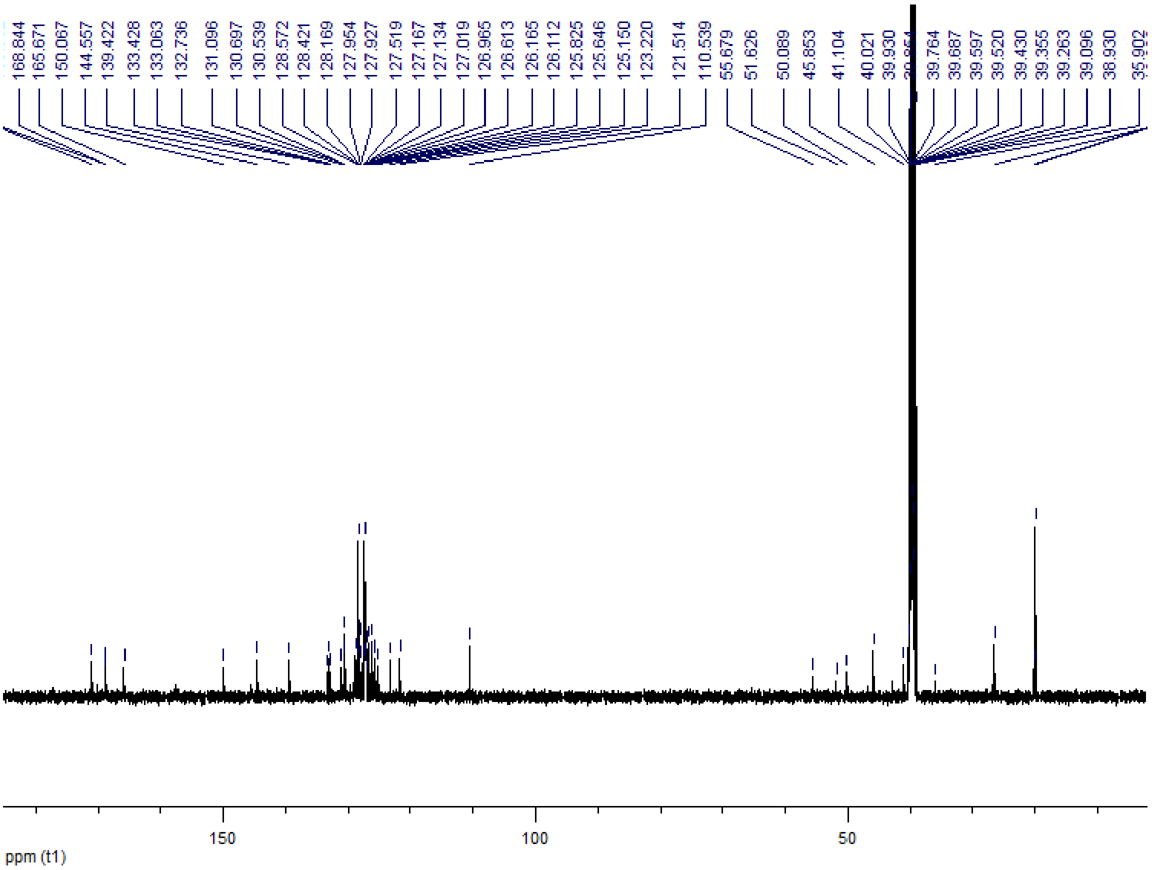

^{1}H NMR Spectra of Compound **2** in DMSO-d_6

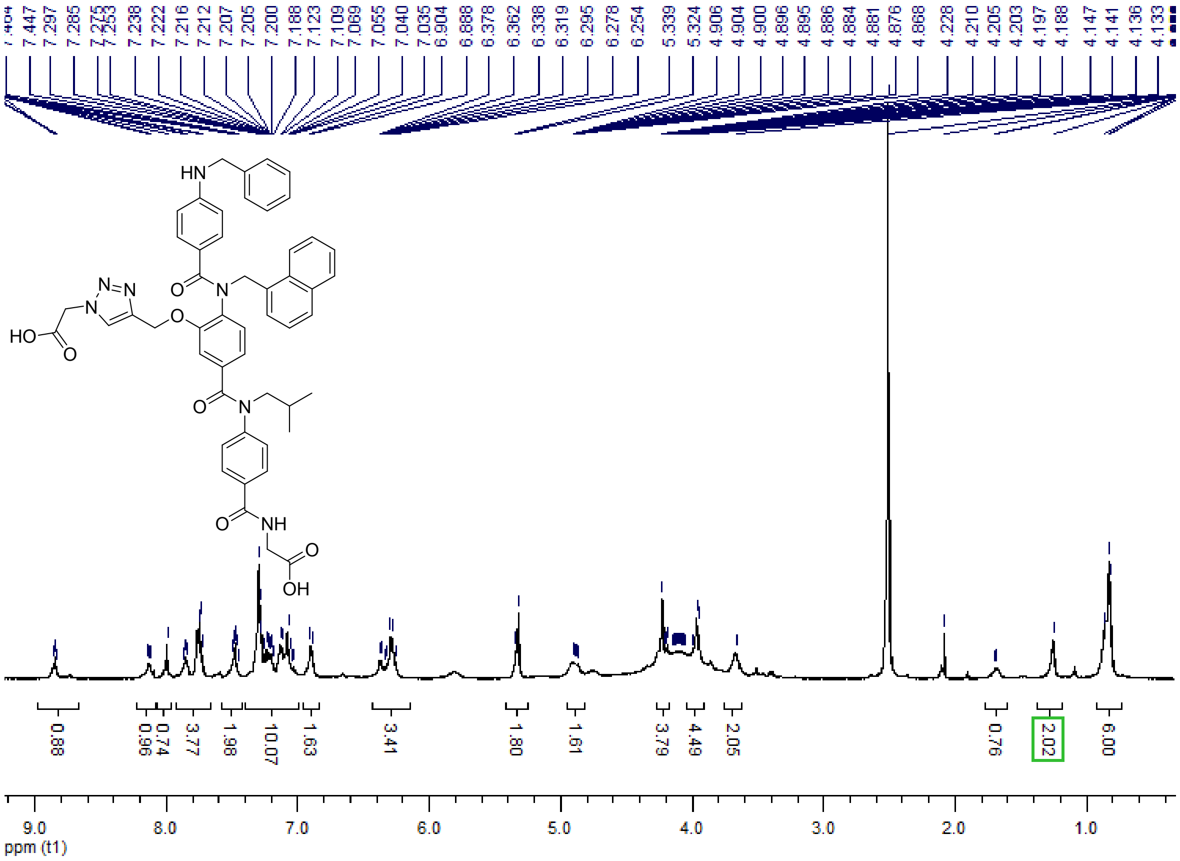

^{1}H-^{1}H COSY NMR Spectra of Compound **2** in DMSO-d_6

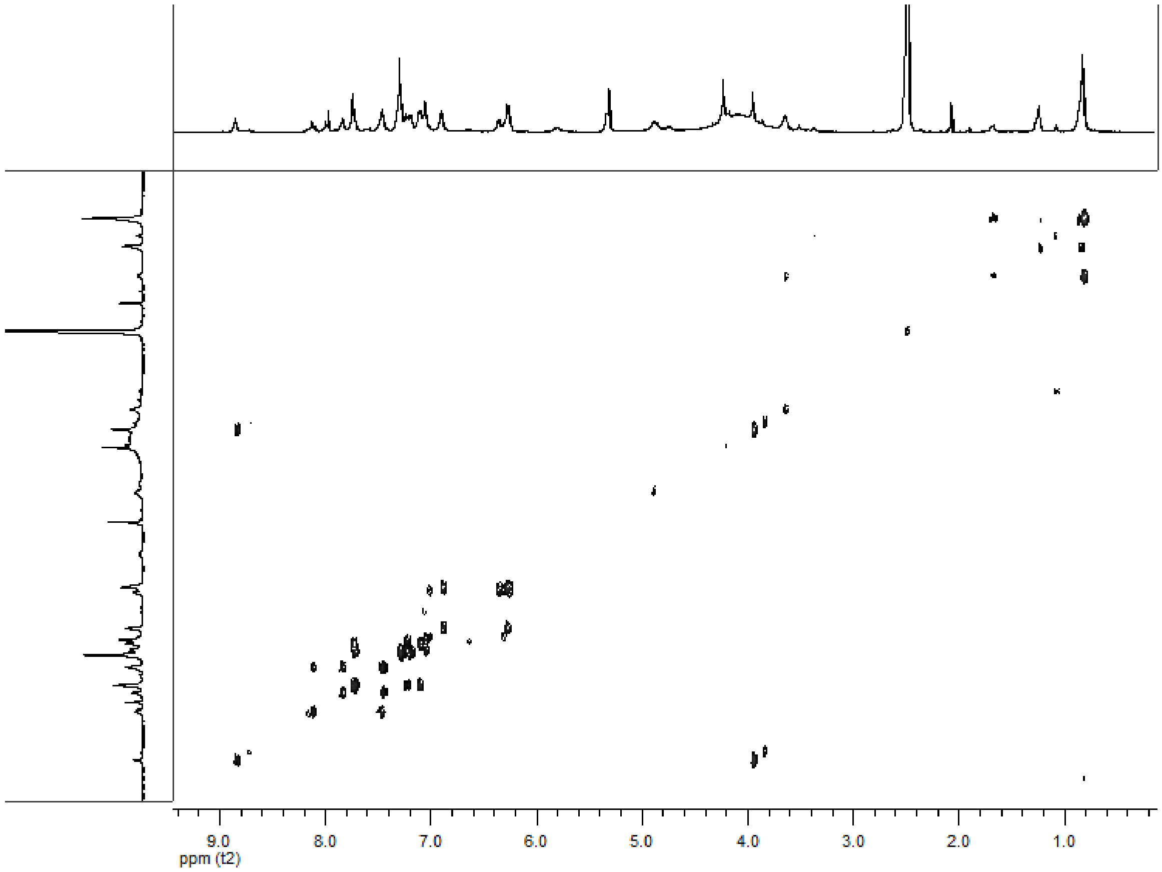

^{1}H-^{1}H NOESY NMR Spectra of Compound **2** in DMSO-d_6

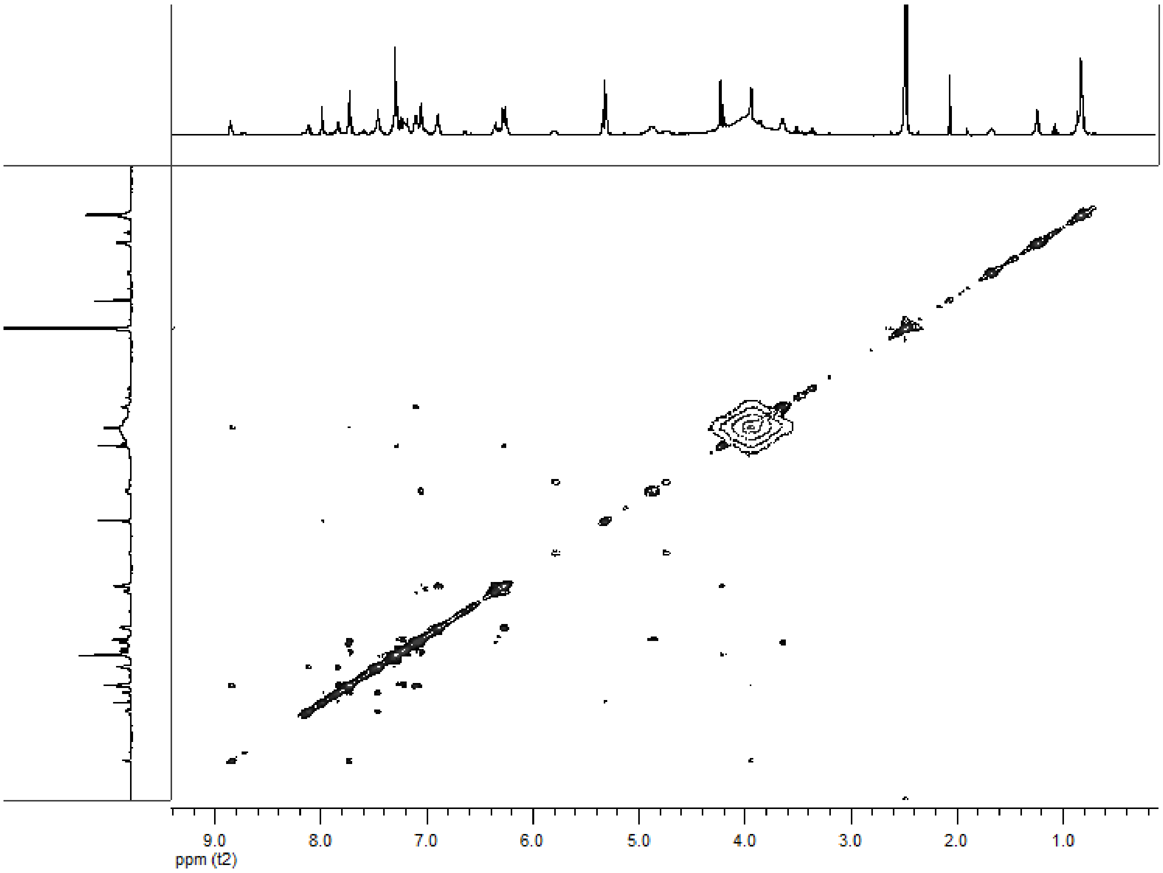

^{13}C NMR Spectra of Compound **2** in DMSO-d_6

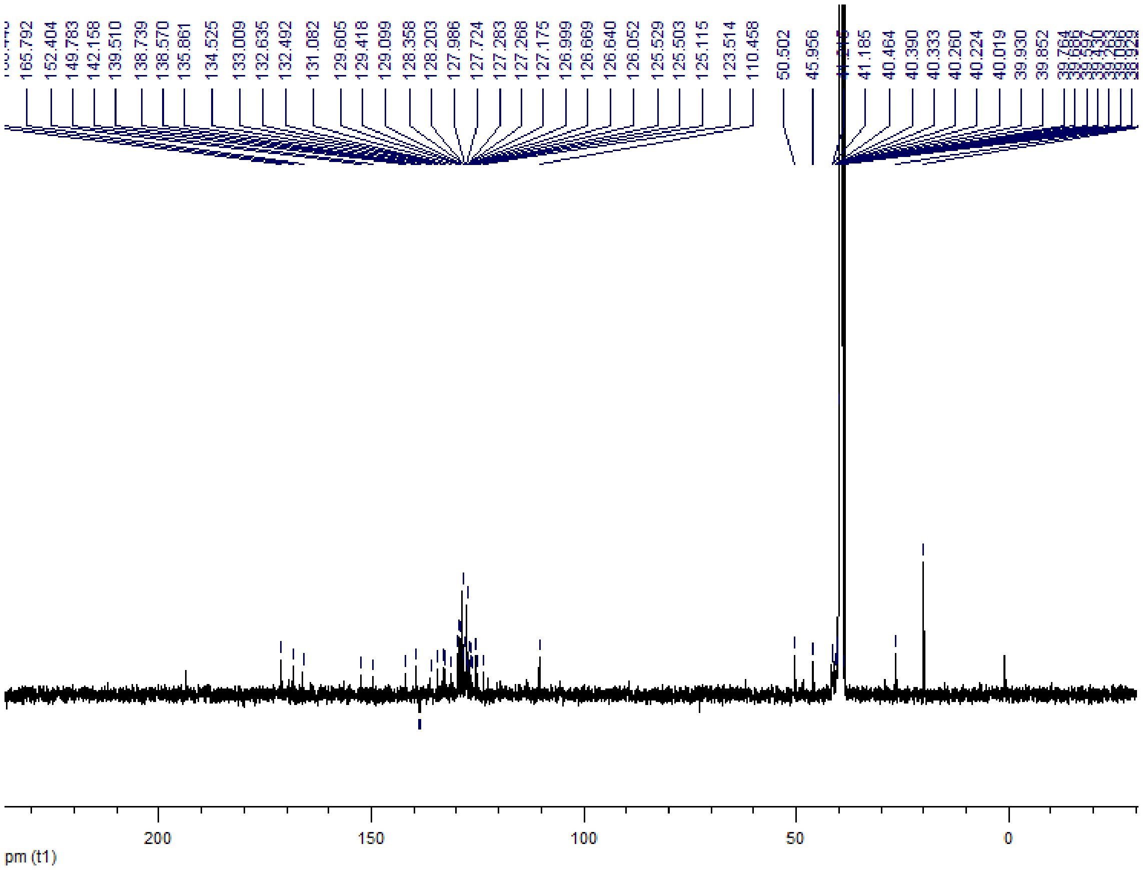

^{1}H NMR Spectra of Compound **3** in DMSO-d_6

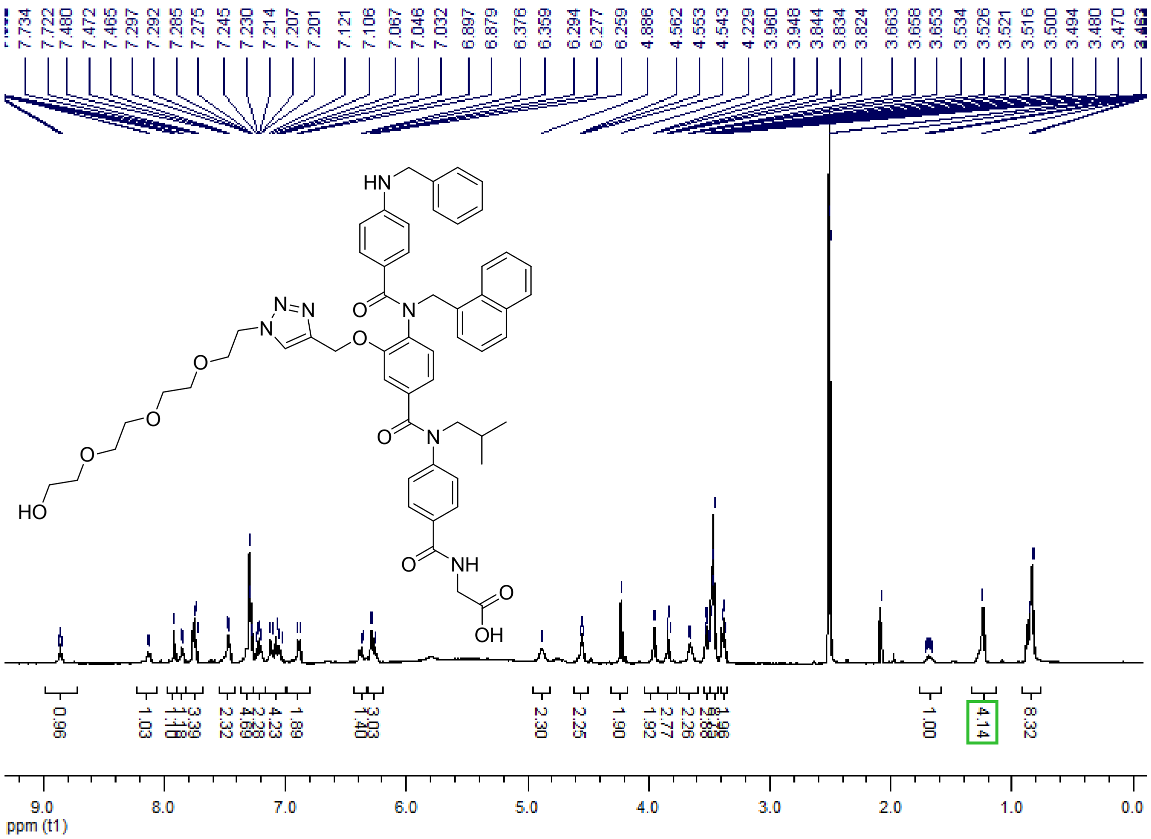

^{1}H-^{1}H COSY NMR Spectra of Compound **3** in DMSO-d_6

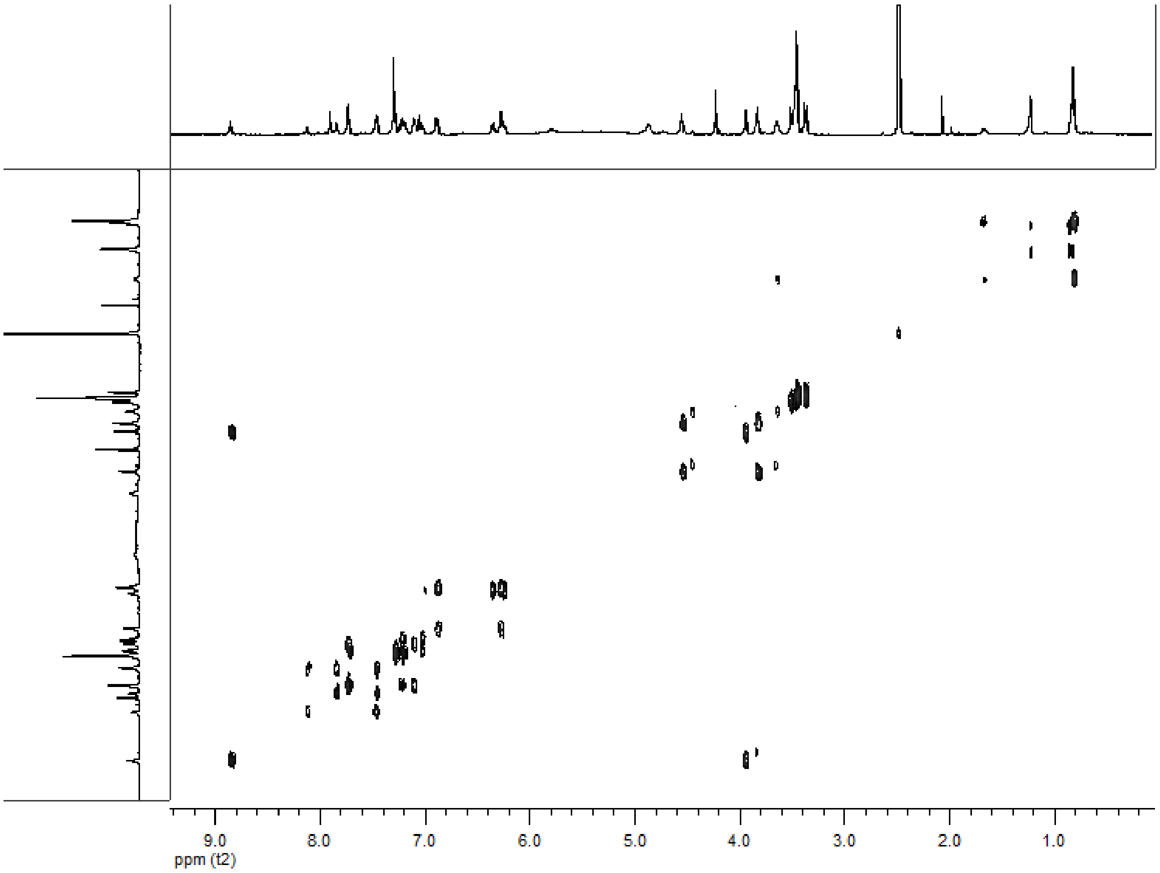

^{1}H-^{1}H NOESY NMR Spectra of Compound **3** in DMSO-d_6

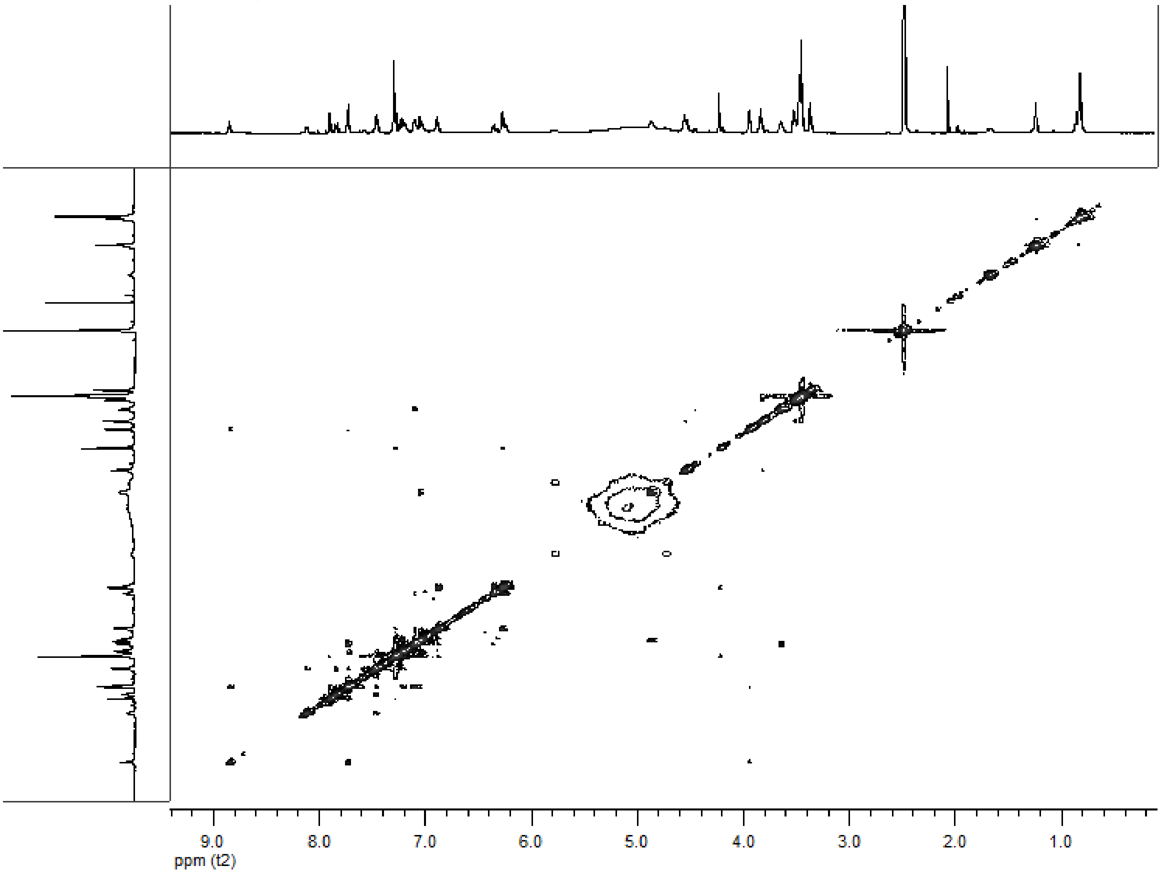

^{13}C NMR Spectra of Compound **3** in DMSO-d_6

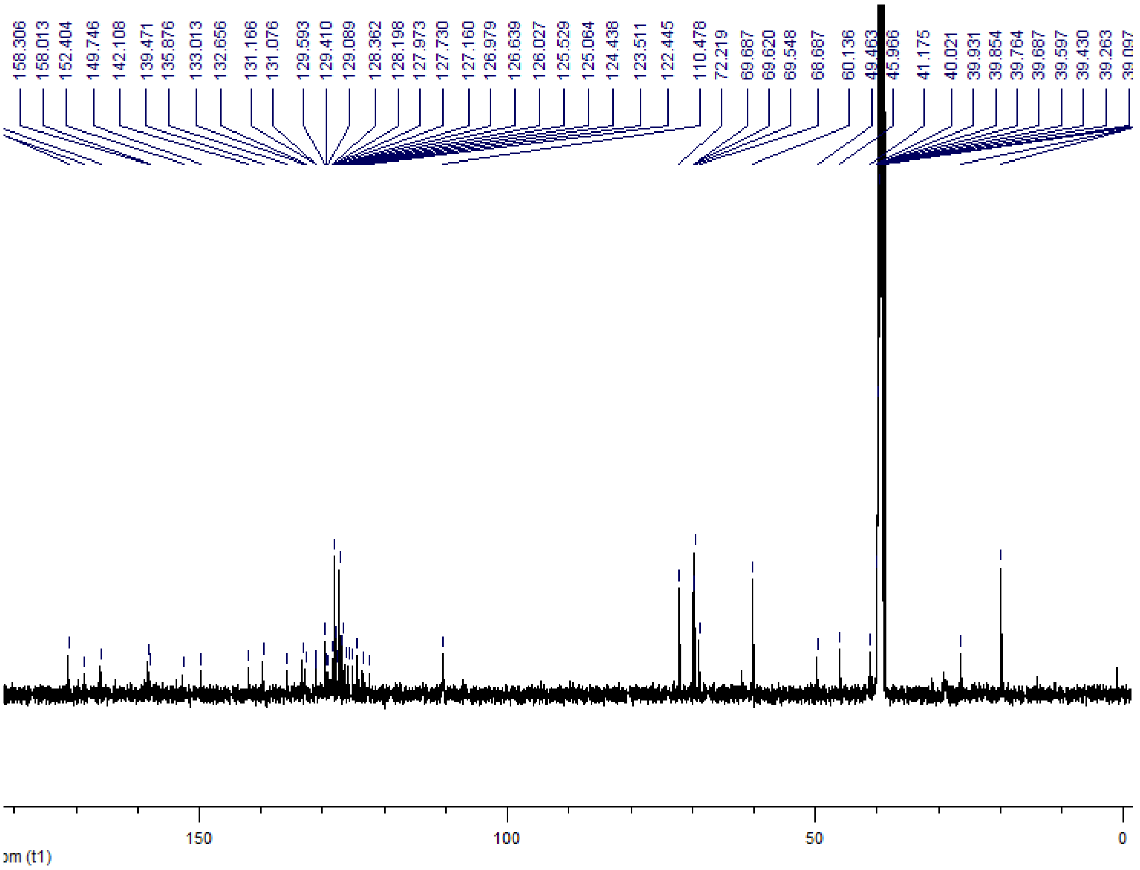

^{1}H NMR Spectra of Compound **14** in $CDCl_3$

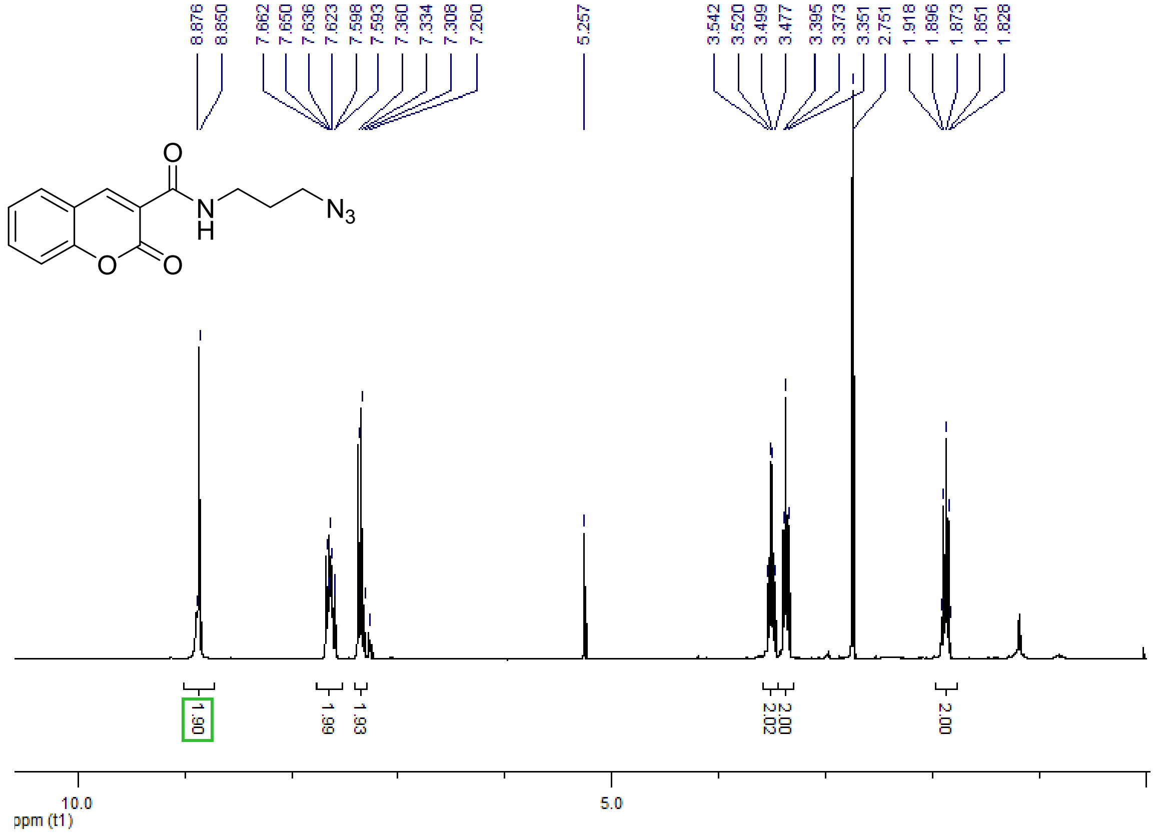

^{13}C NMR Spectra of Compound **14** in $CDCl_3$

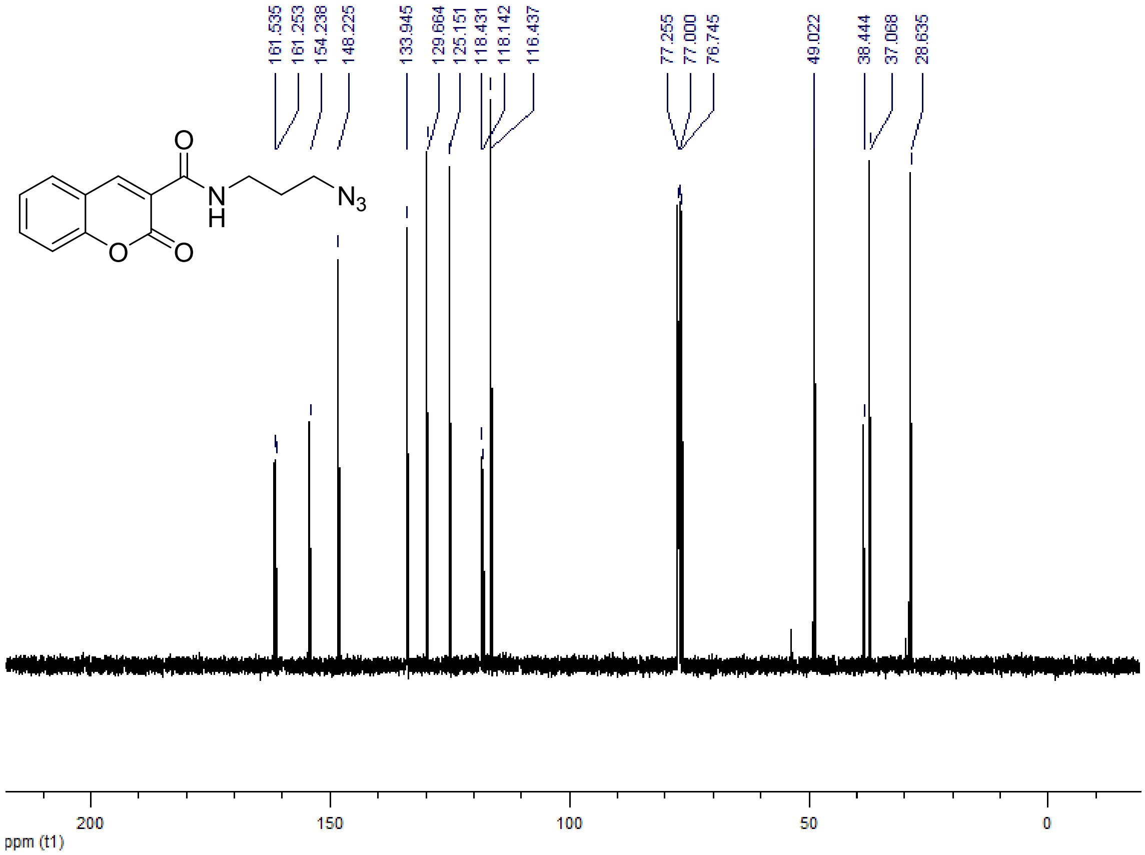

1H NMR Spectra of Compound **15** in DMSO-d_6

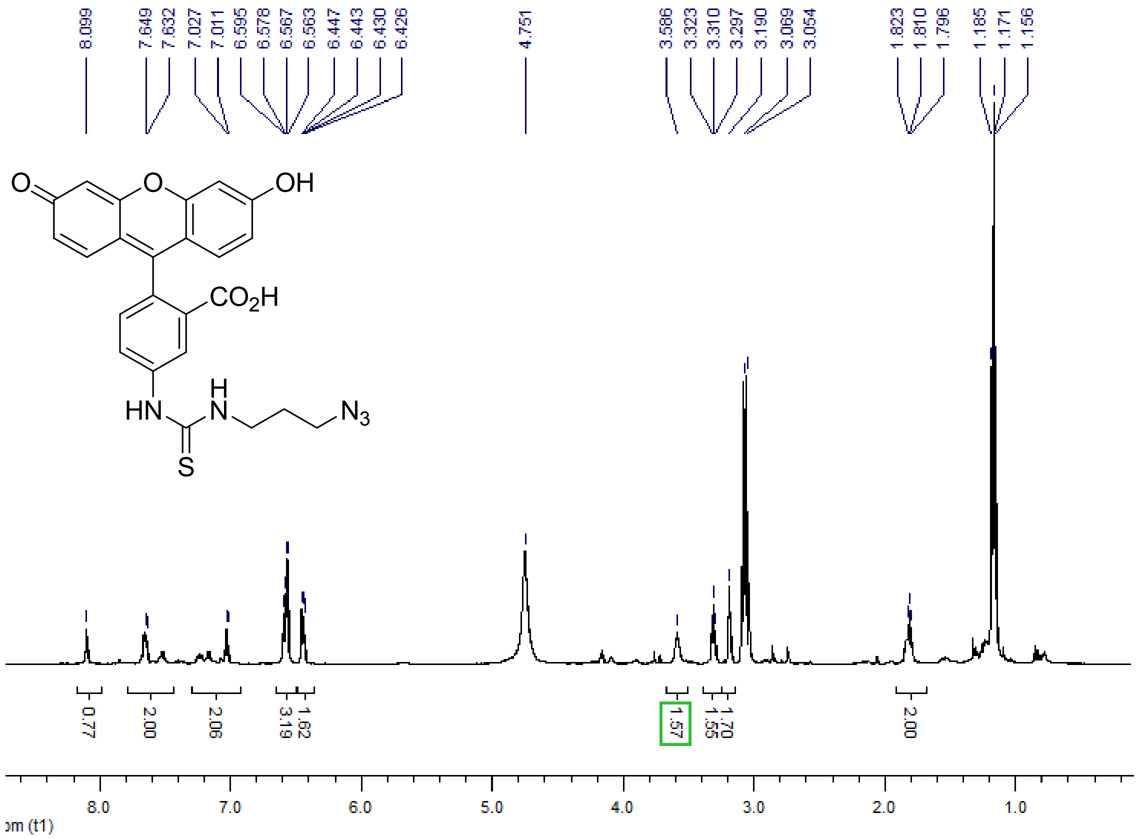

^{1}H NMR Spectra of Compound **4** in DMSO-d_6

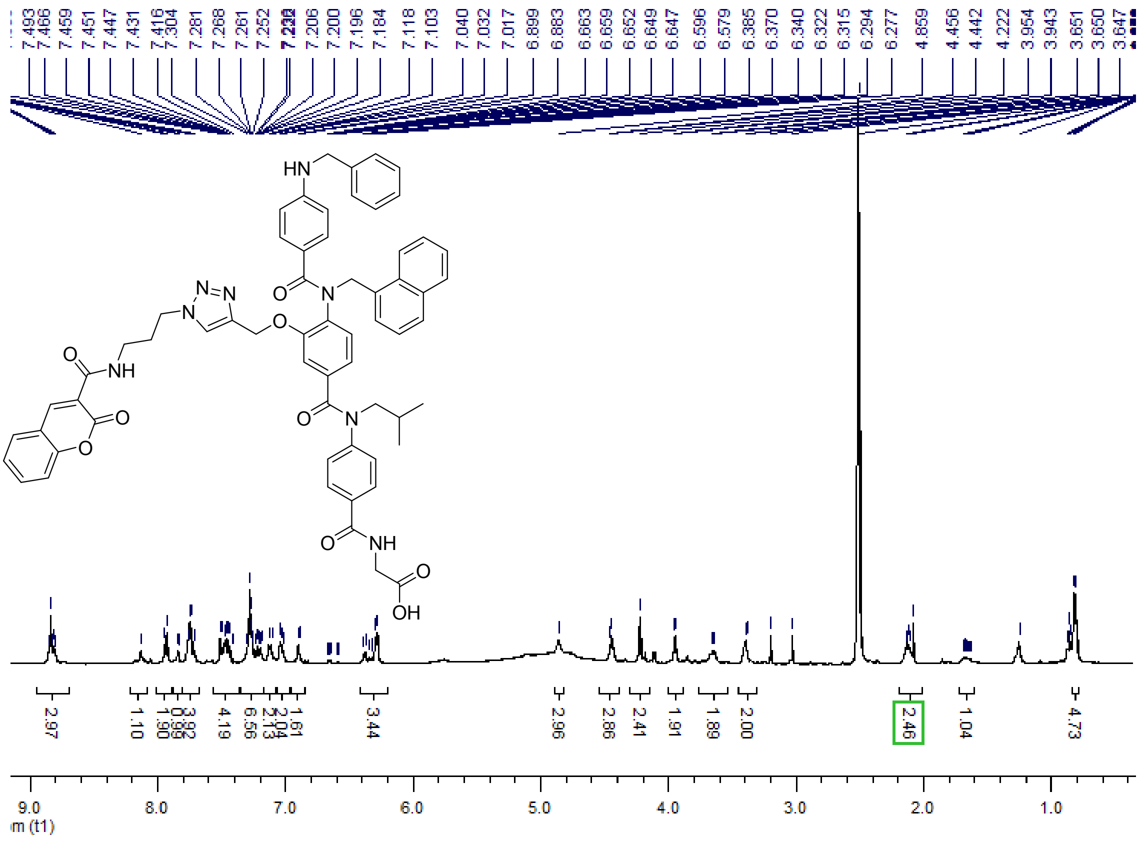

^{1}H-^{1}H COSY Spectra of Compound **4** in DMSO-d_6

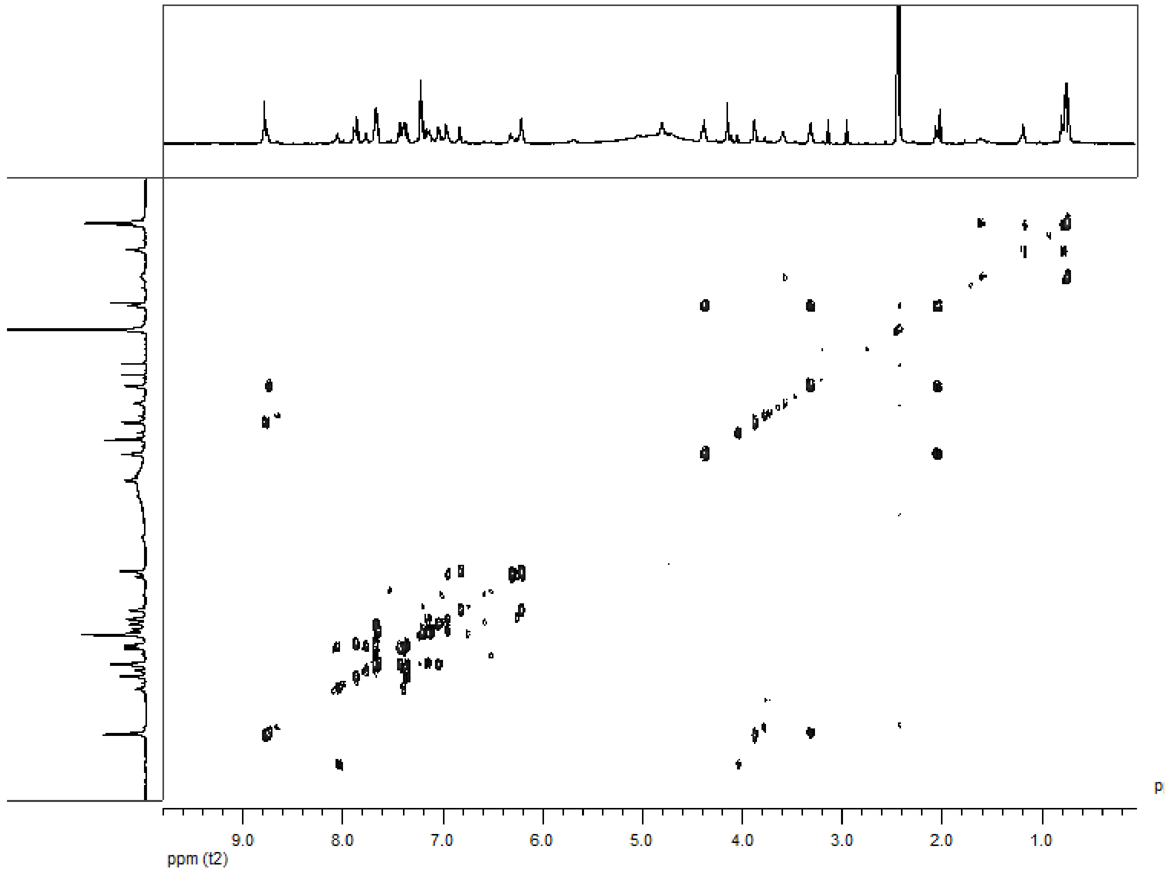

[1]H-^{1}H NOESY Spectra of Compound **4** in DMSO-d_6

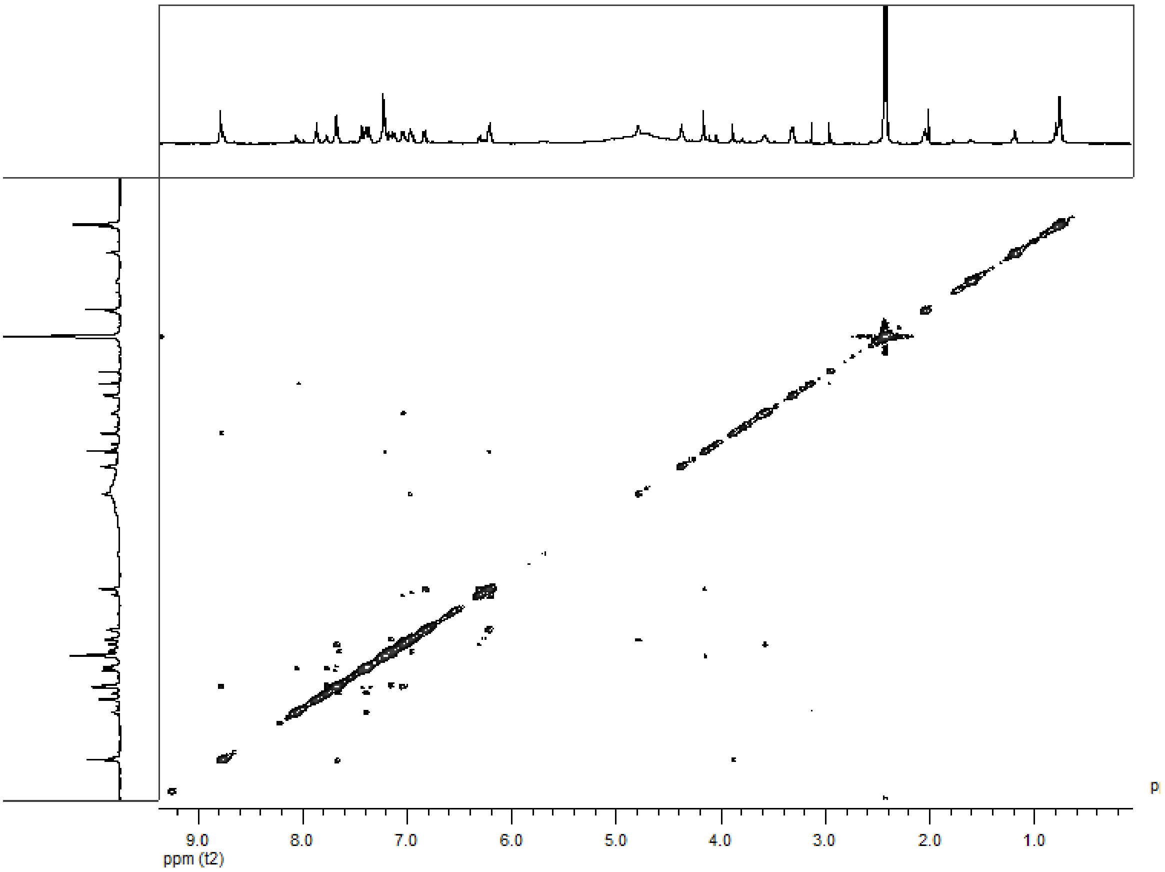

^{13}C NMR Spectra of Compound **4** in DMSO-d_6

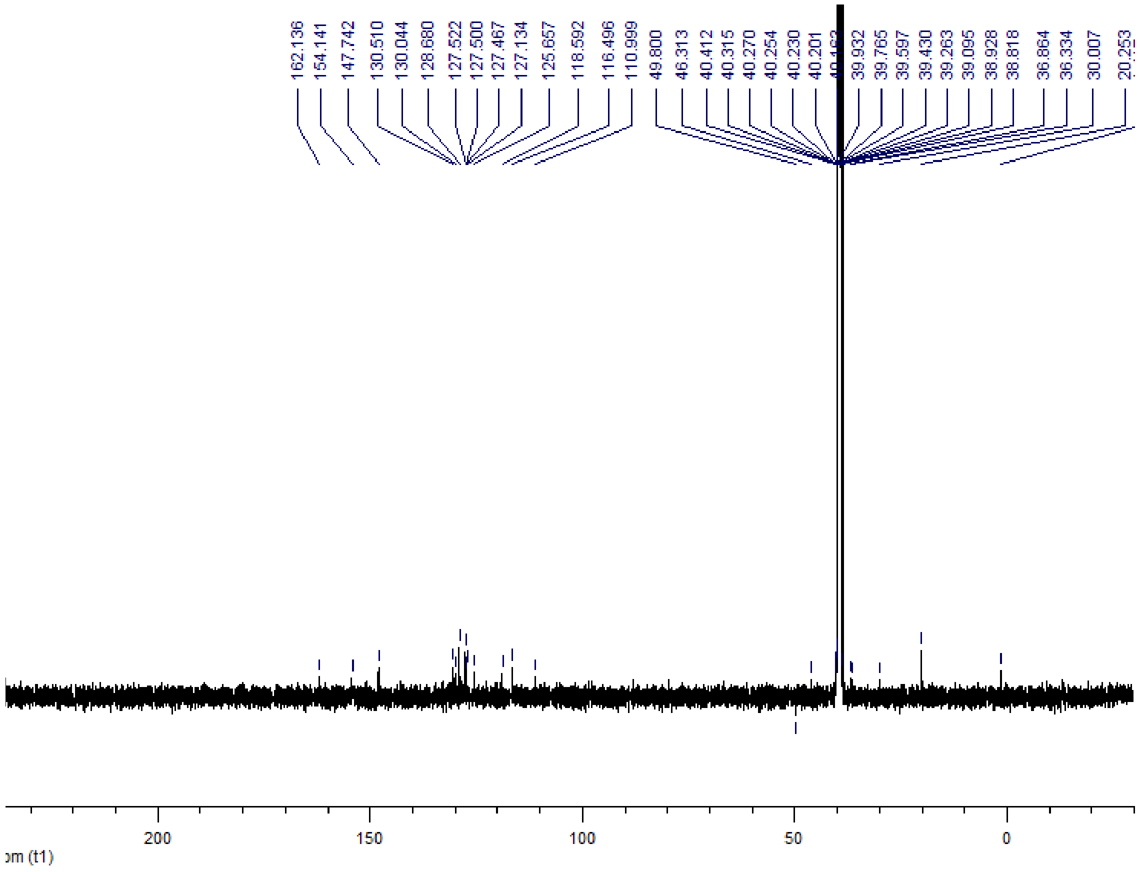

^{1}H NMR Spectra of Compound **5** in DMSO-d$_6$

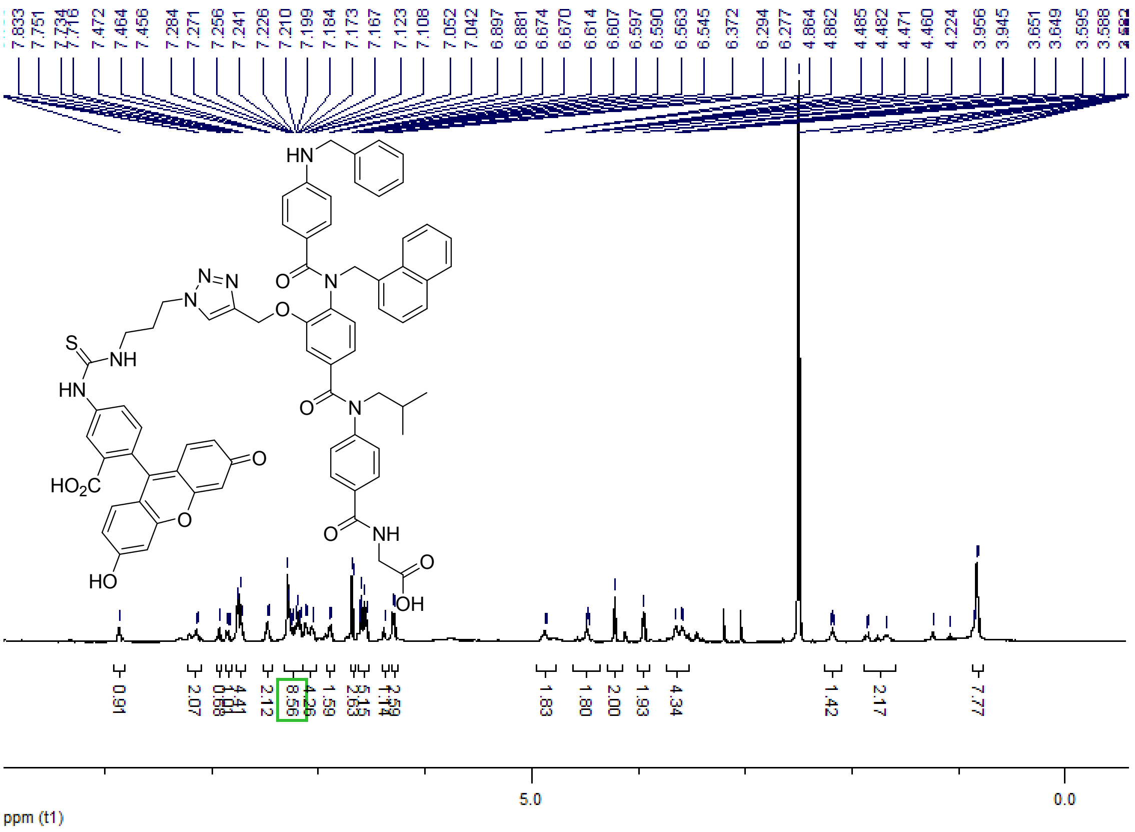

^{1}H-^{1}H COSY Spectra of Compound **5** in DMSO-d$_6$

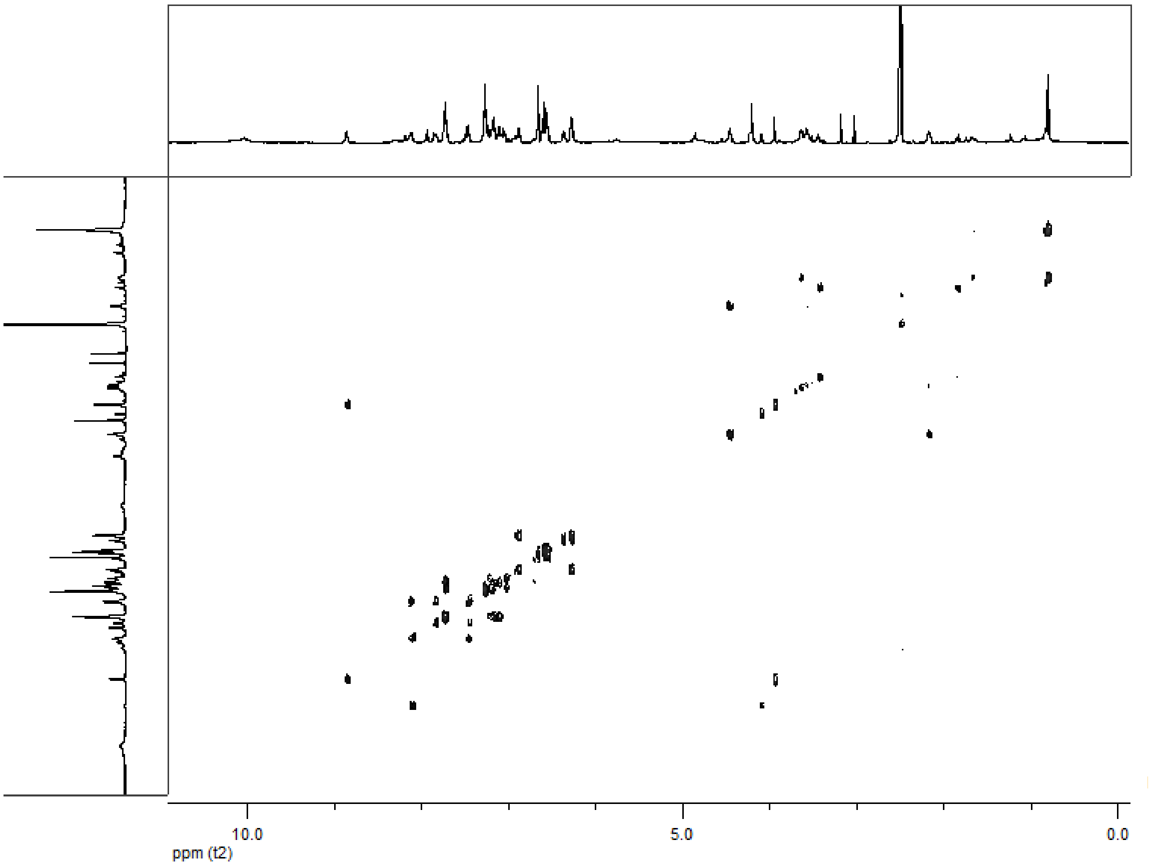

[1]H-^{1}H NOESY Spectra of Compound **5** in DMSO-d_6

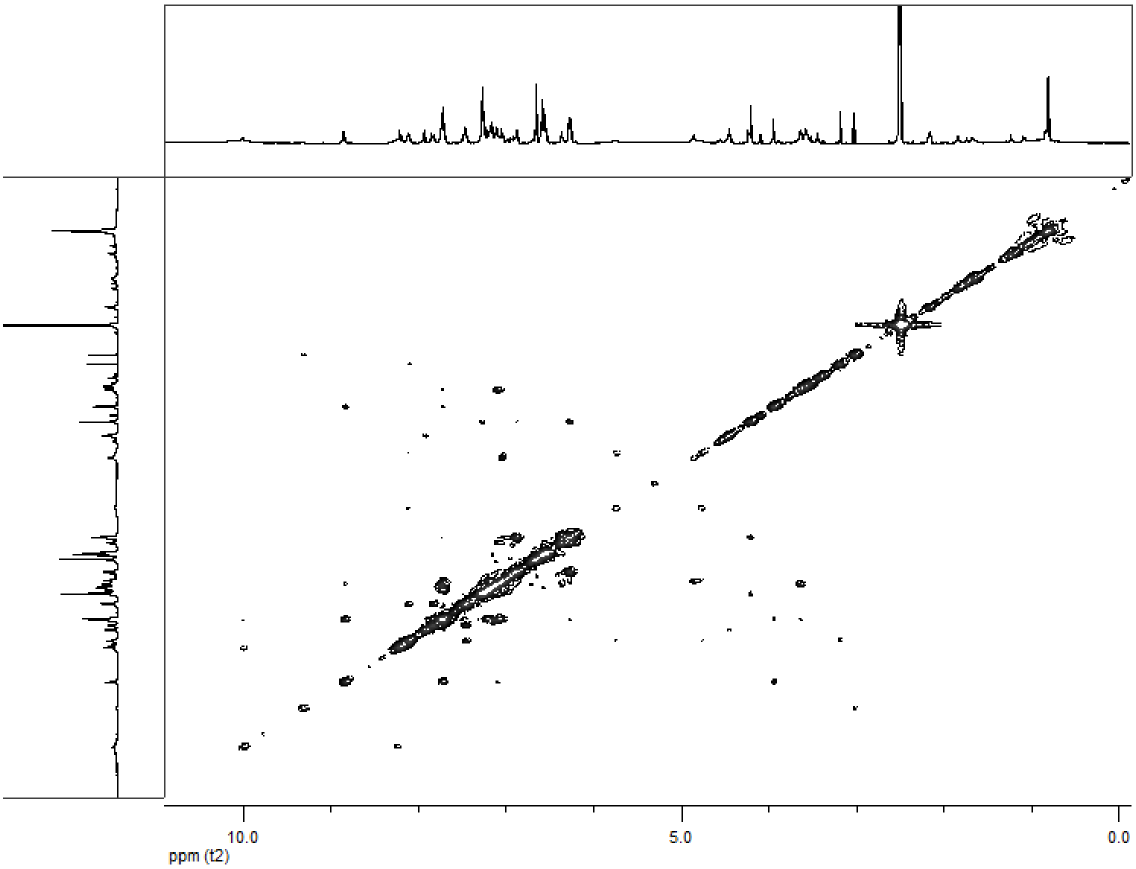

LC-MS and HPLC Traces of Trimers 1- 5

LC-MS Trace of Compound **1**

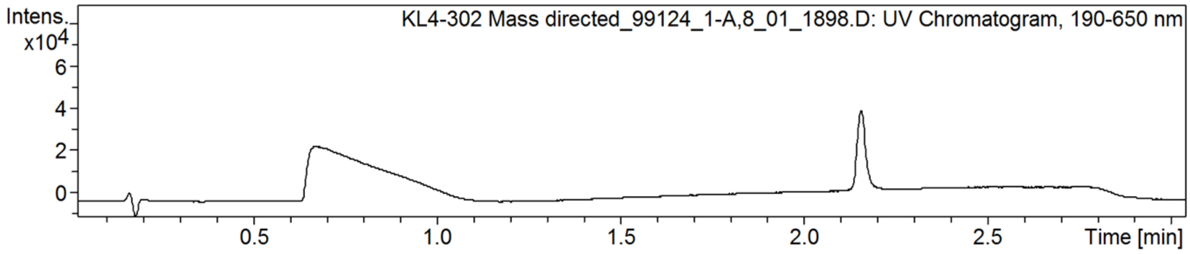

HPLC Trace of Compound **1**

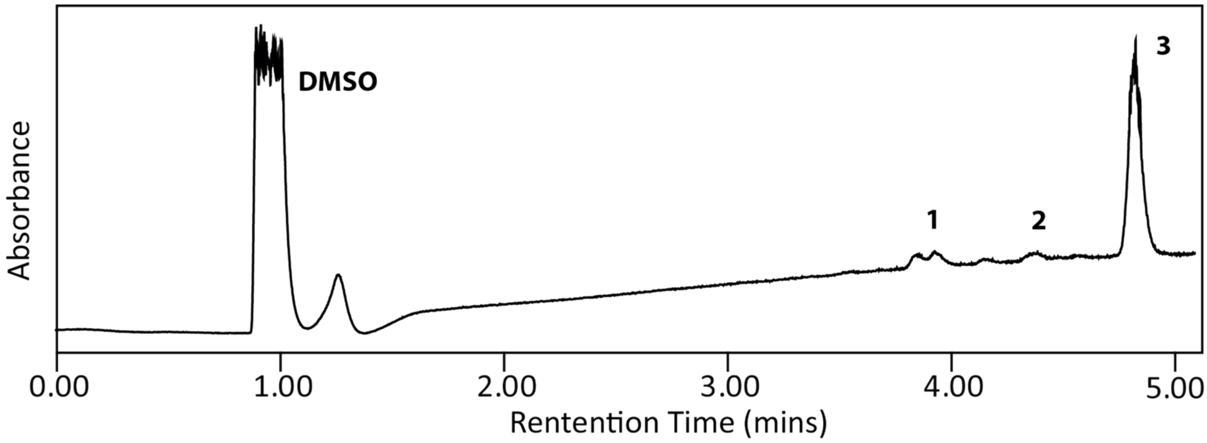

Peak Number	Retention Time	Percentage Area
**1**	3.95	7.53
**2**	4.41	2.23
**3**	4.85	90.24

LC-MS Trace of Compound **2**

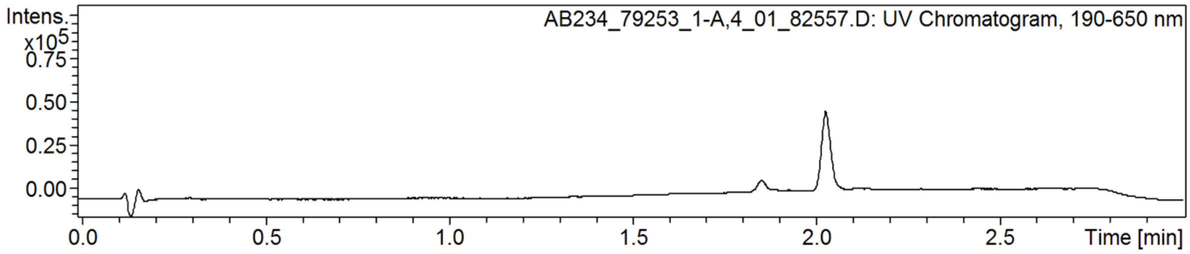

HPLC Trace of Compound **2**

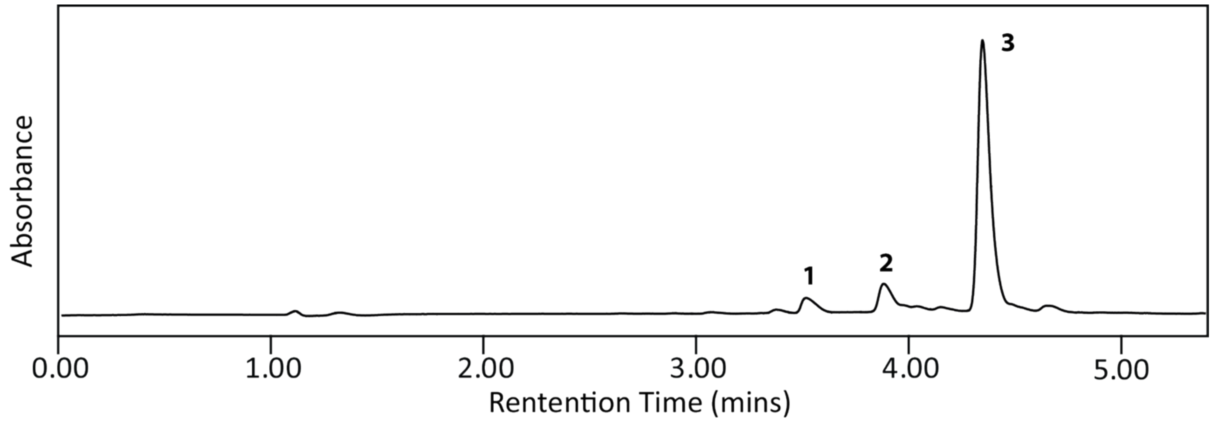

**Peak Number**	**Retention Time**	**Percentage Area**
**1**	3.52	3.58
**2**	3.89	6.82
**3**	4.36	89.60

LC-MS Trace of Compound **3**

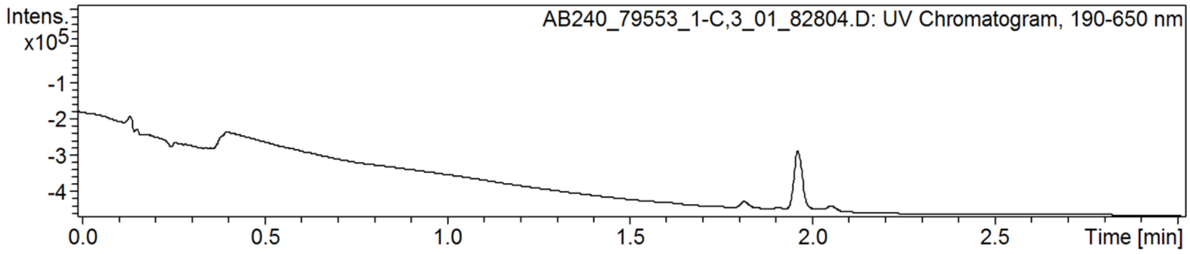

HPLC Trace of Compound **3**

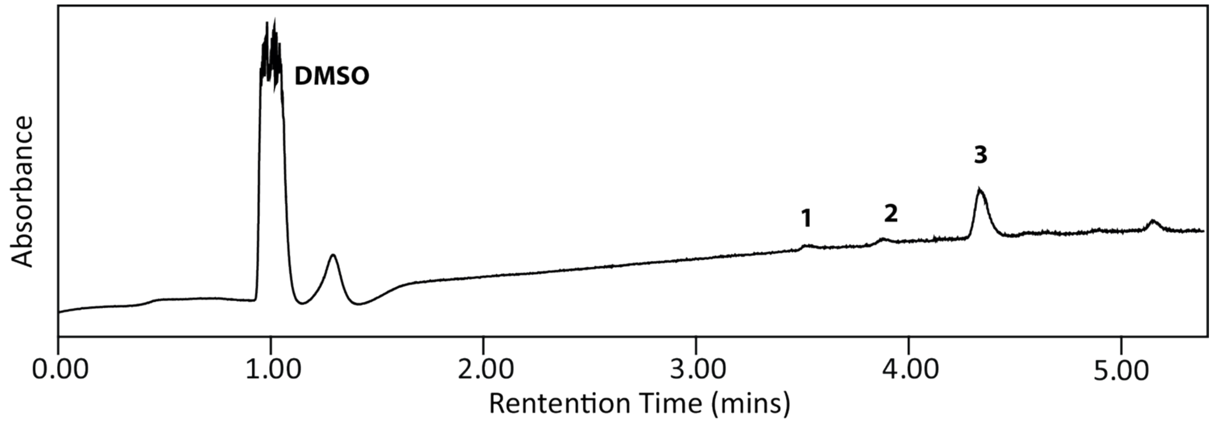

**Peak Number**	**Retention Time**	**Percentage Area**
**1**	3.52	5.42
**2**	3.90	2.52
**3**	4.35	92.06

LC-MS Trace of Compound **4**

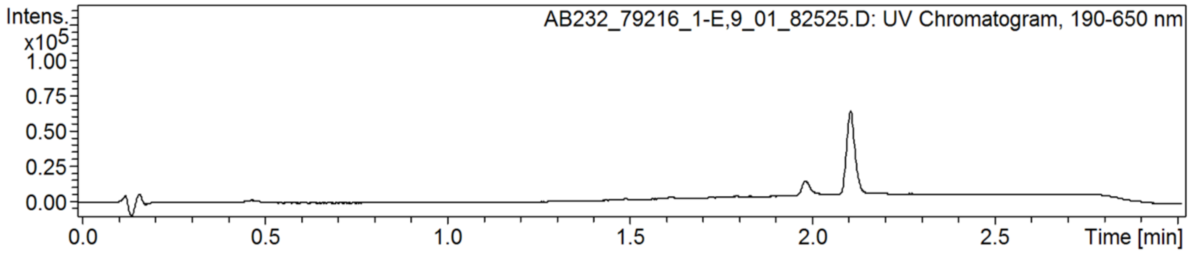

HPLC Trace of Compound **4**

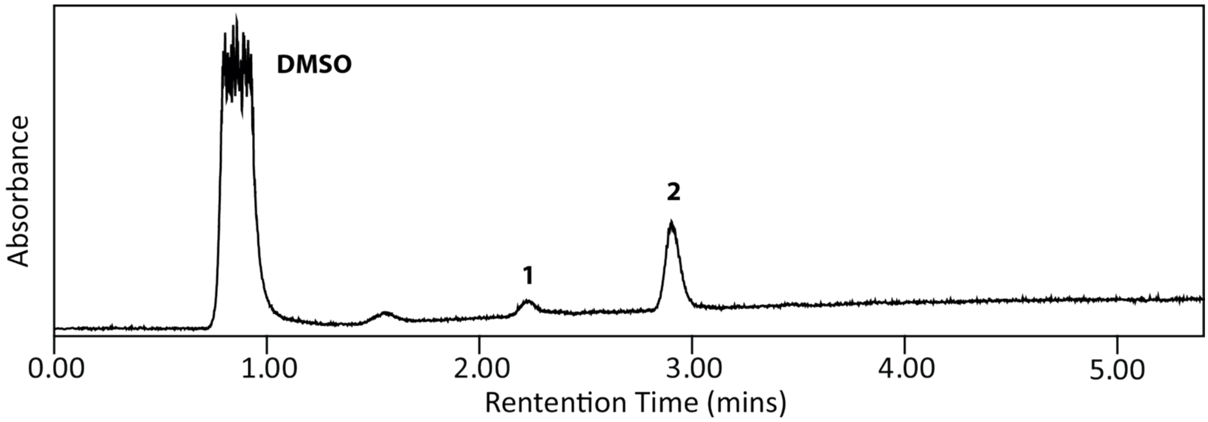

**Peak Number**	**Retention Time**	**Percentage Area**
**1**	2.22	9.18
**2**	2.90	90.82

LC-MS Trace of Compound **5**

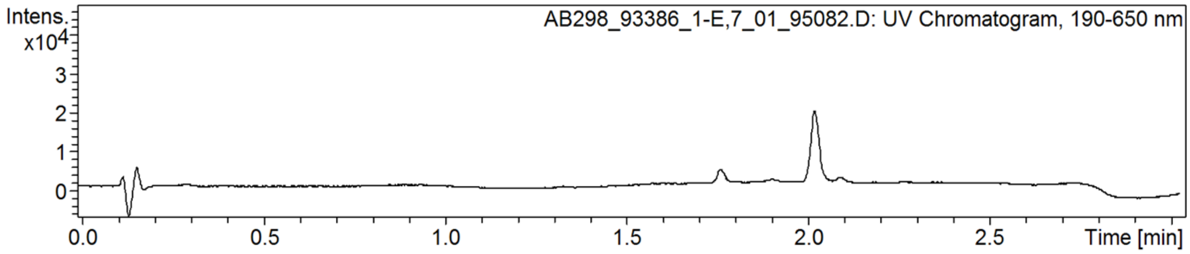

An HPLC trace of Compound **5** was not obtained as the fluorescein label caused extensive broadening of the signal and traces of the compound proved difficult to remove from the column so further attempts were not made.

Synthesis of Coumarin and Fluorescein Azides

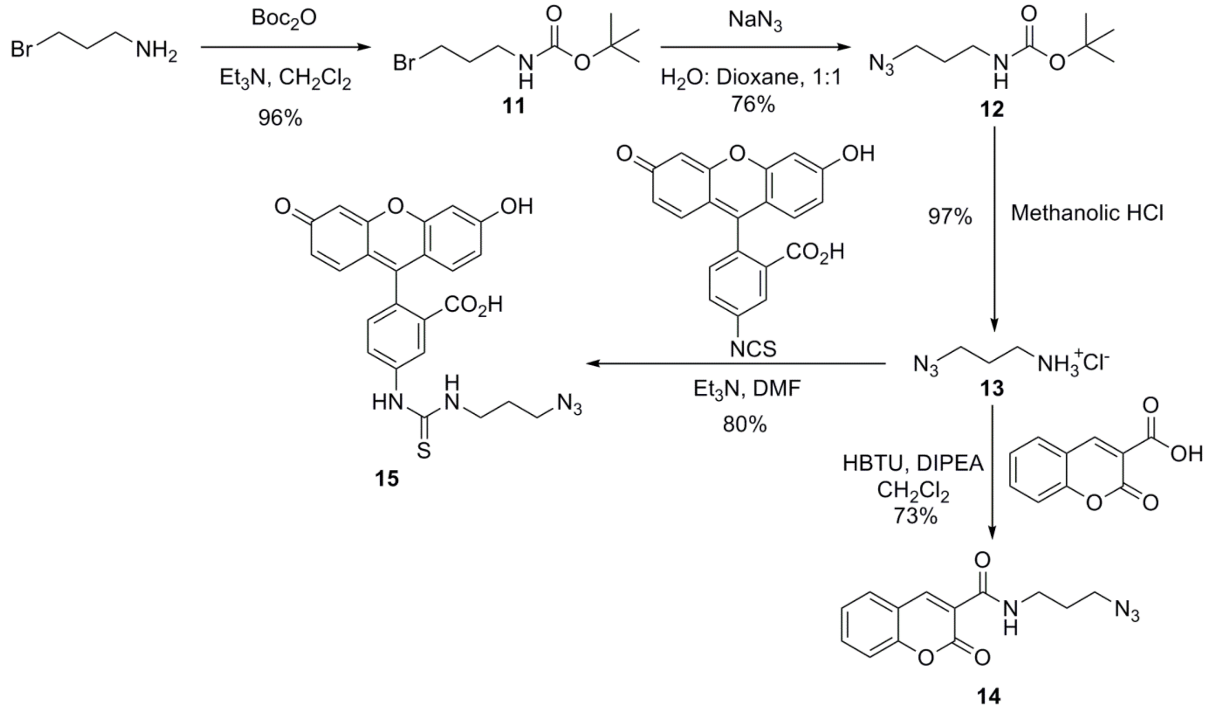

Scheme S1 – Synthesis of azide-functionalised fluorophores

Molecular Modelling

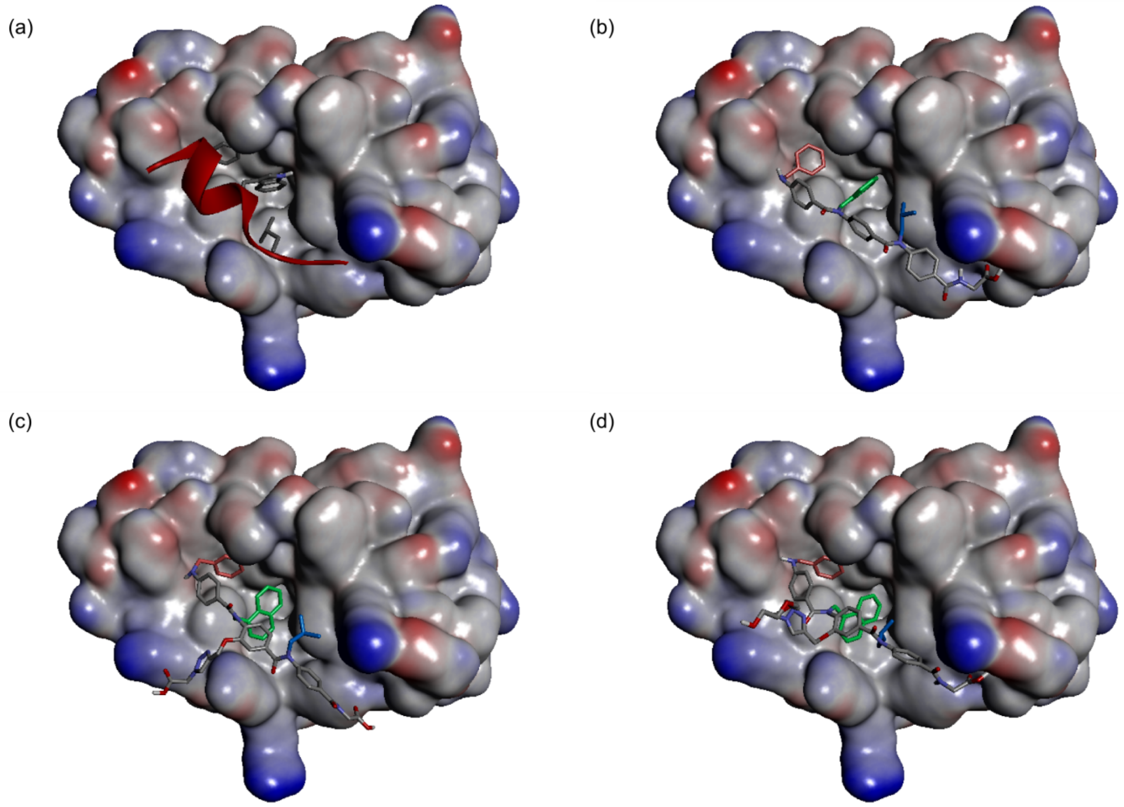

Figure S1 – Helix mimetics were docked to the surface of hDM2 (a) p53/hDM2 structure (PDB ID: 1YCR) (b) unfunctionalised methyl trimer **1** (c) acid funcitonalised trimer **2** (d) PEG functionalised trimer **3**

An all-methyl version of the unfunctionalised and the 'click' trimers were energy minimised and the energy change upon rotation of the central *N*-aryl bond was determined.

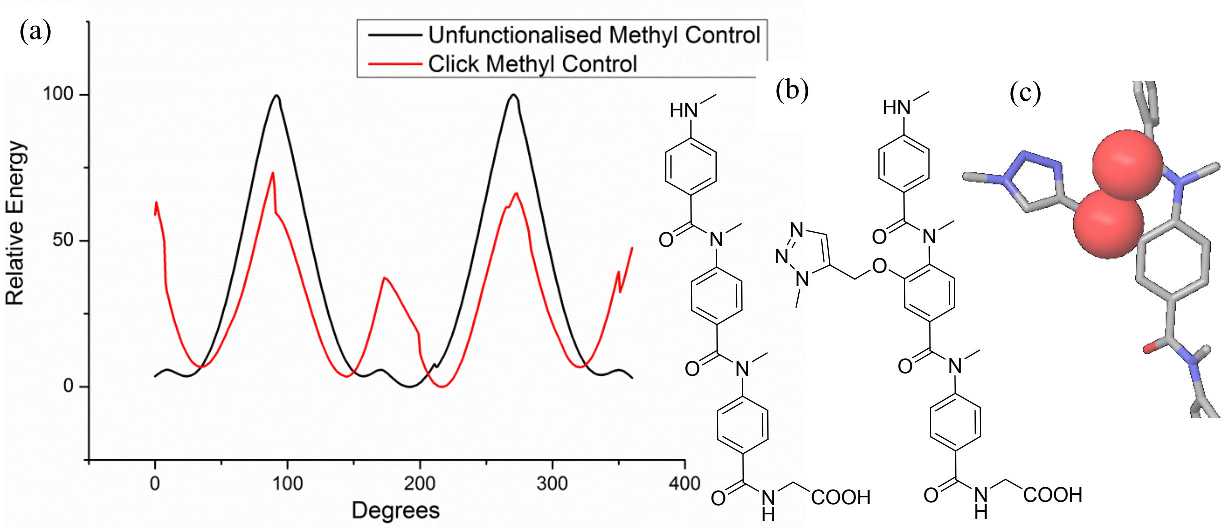

Figure S2 – (a) Relative energies at different degrees of rotation about the central *N*-aryl bond of the (b) unfunctionalised methyl trimer (left) and the 'click' functionalised methyl trimer (right) (c) steric clash in the functionalised trimers, the carbonyl and side chain oxygen atoms are shown in CPK format.

Fluorescence Anisotropy

Mcl-1/NOXA-B Assay Development

Mcl-1 purification

The pet28a His-SUMO Mcl-1 (172-327) construct was over-expressed in the *E.coli* strain Rosetta 2. 10 ml of overnight starter culture was used to inoculate 1 L 2 xYT containing 50 µg/ml Kanamycin and 50ug/ml Chloramphenicol. Cultures were grown at 37 °C plus shaking until OD600 ~ 0.6 – 0.8, the temperature was then switched to 18 °C and protein expression induced by the addition of 0.8mM IPTG. Induced cultures were grown at 18 °C plus shaking overnight before harvesting by centrifugation. Cells were resuspended in 50 mM TRIS pH 8.0, 500 mM NaCl, 15 mM imidazole and lysed by sonication in the presence of 10 µL of 1 U.ml-1 DNase I per litre of over-expression culture and 5 mM $MgCl_2$. The cell lysate was centrifuged (Beckman JA25.50 rotor, 17,000 rpm, 45 min, 4 °C) and the supernatant filtered (0.22 µM syringe filter) before application onto a 5 ml HisTrap that had previously been equilibrated with 50 mM TRIS pH 8.0, 500 mM NaCl, 15 mM imidazole. The cleared cell lysate was then allowed to flow through the HisTrap with the aid of a peristaltic pump. The HisTrap was then washed with 10 CV of 50 mM TRIS pH 8.0, 500 mM NaCl, 15 mM imidazole followed by 10 CV 50 mM TRIS pH 8.0, 500 mM NaCl, 50 mM imidazole. The His-SUMO-Mcl-1 fusion protein was then eluted from the HisTrap with 50 mM TRIS pH 8.0, 500 mM NaCl, 300 mM imidazole. The His-SUMO-Mcl-1 fusion protein was cleaved overnight in dialysis into 50 mM TRIS pH 8.0, 250 mM NaCl in the presence of Smt3 protease, Ulp1, overnight at 4 °C. To remove any uncleaved Mcl-1, His-SUMO and Ulp1, the sample was reapplied to a HisTrap in 50 mM TRIS pH 8.0, 250 mM NaCl and the flow through containing Mcl-1 collected. The flow through containing cleaved Mcl-1 was concentrated (Amicon Ultra centrifugal filter, MWCO 10,000) to approximately 5 ml. The sample was then filtered before being loaded onto a Superdex 75 column (GE healthcare) equilibrated in 50 mM TRIS pH 8.0, 250 mM NaCl, 0.5 mM DTT, 2.5% Glycerol. The purified was concentrated to ~ 6 mg/ml and stored at – 80 °C.

Determination of the binding of FITC-NOXA-B to Mcl-1

The procedure followed was adapted from our previous paper on *O*-alkylated aromatic oligoamides.[1] Briefly: Mcl-1 was serially diluted (446 pM – 5 µM) into a solution of FITC-NOXA-B (25 nM) (40 mM Sodium phosphate buffer pH 7.54, 200 mM NaCl, 0.02 mg/ml of bovine serum albumin) – the total volume of each well was 100 µL, the plates were allowed to incubate at room temperature for 45 minutes. Each experiment was run in triplicate and the fluorescence anisotropy measured using a Perkin Elmer EnVisionTM 2103 MultiLabel plate reader, with excitation at 480 nm and emission at 535 nm (30 nM bandwidth) and the intensity (Eq. 1) was calculated for each point. This was used to calculate anisotropy (Eq. 2) and plotted to a sigmoidal fit in Origin 7 to determine the minimum and maximum anisotropies (r_{min} and r_{max}). Using equation 3, the data for the anisotropy was converted to fraction bound and multiplied by the FITC-NOXA-B concentration then fitted in origin 7 (Eq. 4) to give the dissociation constant K_d = 18.7 ± 0.9 nM. From a plot of intensity (Eq. 1) against concentration of protein λ was calculated to be 1.

$$I = (2PG) + S \quad \textit{Equation 1}$$

$$r = \frac{S - PG}{I} \quad \textit{Equation 2}$$

$$L_b = \frac{(r - r_{min})}{(\lambda(r_{max} - r) + r - r_{min}} \quad \textit{Equation 3}$$

$$y = \frac{\{(k_1 + x + [FL] - \sqrt{\{(k_1 + x + [FL]^2 - 4x[FL]\}}}{2} \quad \textit{Equation 4}$$

r = anisotropy, I = total intensity, P = perpendicular intensity, S = parallel intensity, L_b = fraction ligand bound, $\lambda = I_{bound}/I_{unbound} = 1$, [FL] = concentration of fluorescent ligand, $k_1 = K_d$, $y = L_b$* FITC-NOXA-B and x = [added titrant], G is an instrument factor set to 1.

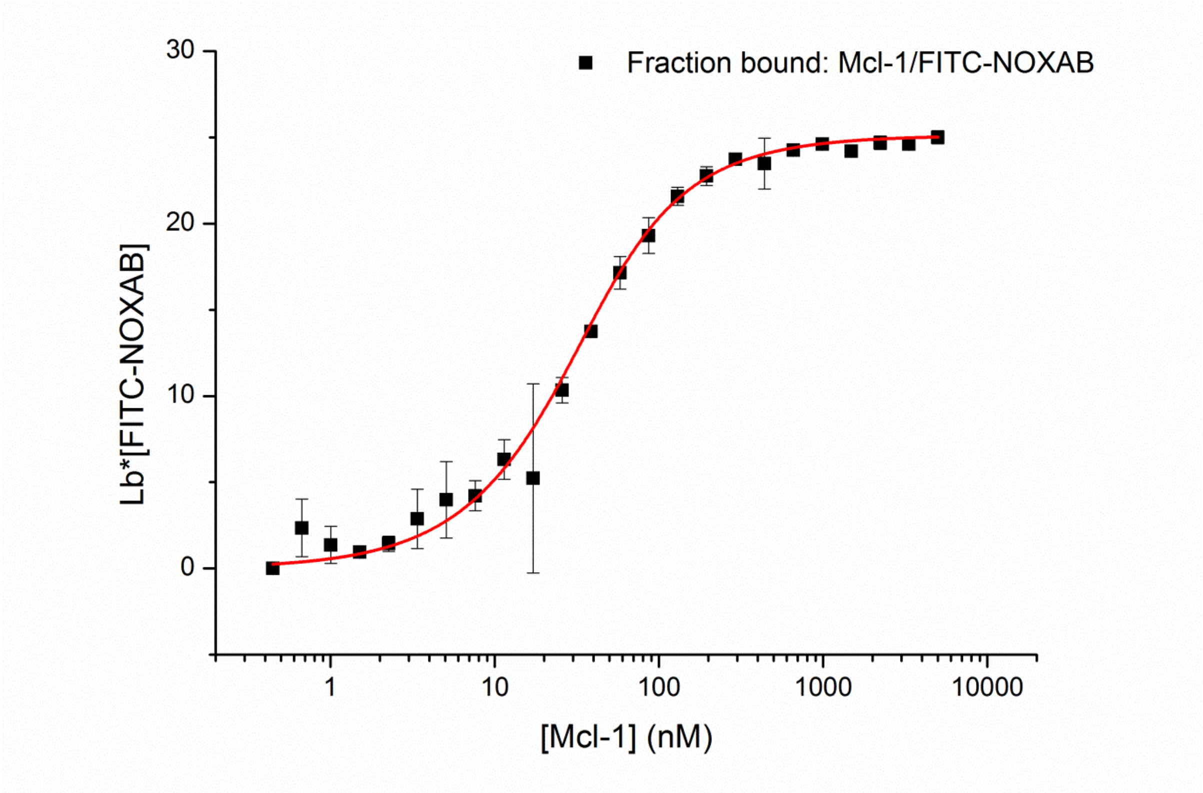

Figure S3 - Fluorescence anisotropy titration assays for the addition of Mcl-1 to a constant concentration of FITC-NOXA-B. Performed in phosphate buffer pH 7.5, 200 mM NaCl, 0.02 mg/ml BSA.

A reverse titration was also performed whereby the protein concentration was held constant and FITC-NOXA-B was serially diluted. The intensity and anisotropy was calculated for each point as before and converted to a fraction bound using the same value of r_{min} and r_{max}. As saturation approaches, the anisotropy begins to drop, whilst the intensity increases, so the asymptote is not reached in this experiment. The fraction bound was then multiplied by the total FITC-NOXA-B concentration at each point and fit (Eq. 4) in origin 7, where y is the fraction bound times the tracer concentration and x is the concentration of added tracer, to give the dissociation constant $K_d = 13 \pm 2$ nM.

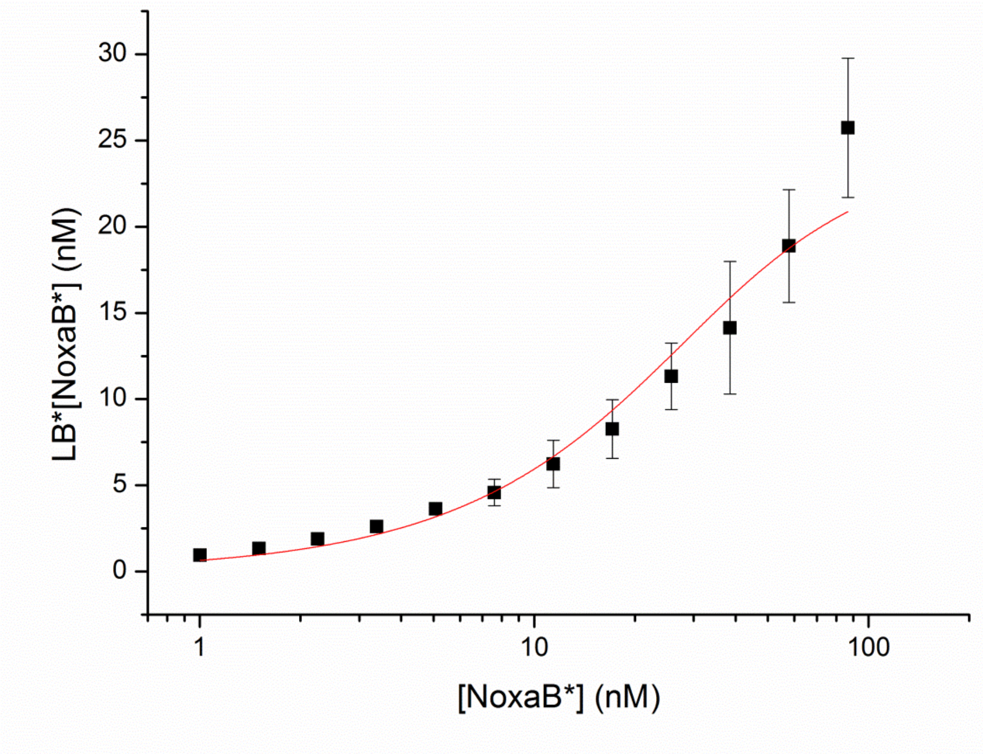

Figure S4 - Fluorescence anisotropy titration assays for the addition of FITC-NOXA-B to a constant concentration of Mcl-1. Performed in phosphate buffer pH 7.5, 200 mM NaCl, 0.02 mg/ml BSA.

In order to set up displacement assay conditions Mcl-1 and FITC-labelled NOXA-B concentrations were fixed and the tracer peptide was displaced with unlabelled NOXA-B.

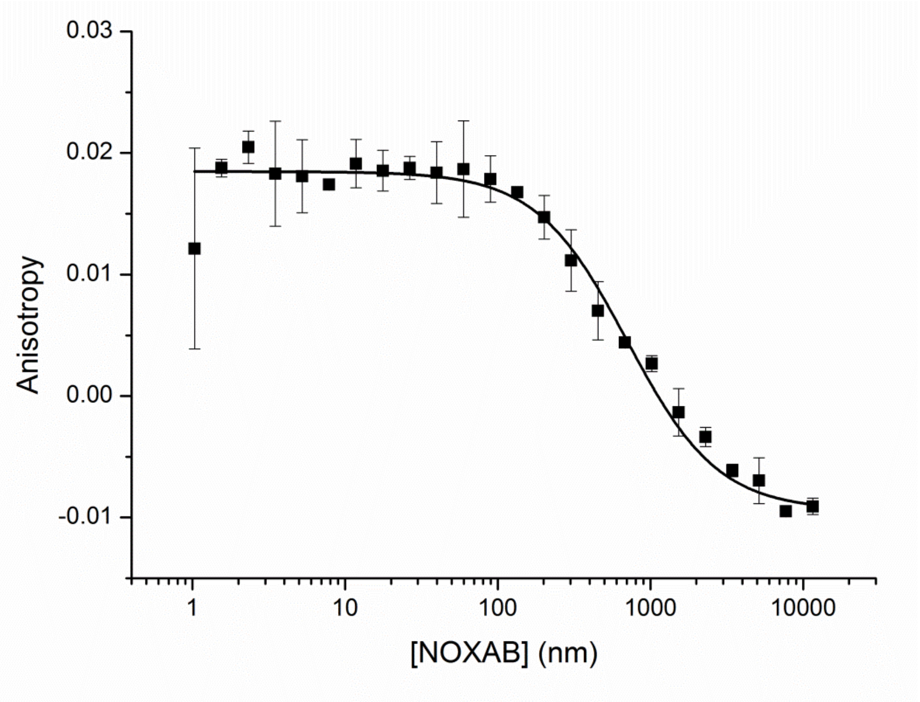

Figure S5 – Fluorescence anisotropy competition assay testing the inhibition of unlabelled NOXA-B against the Mcl-1/NOXA-B interaction. Performed in phosphate buffer pH 7.5, 200 mM NaCl, 0.02 mg/ml BSA. Mcl-1 is fixed as 150 nM and FITC-NOXA-B is at a concentration of 50 nM.
IC_{50} = 704 ± 35 nM.

Competition Assays

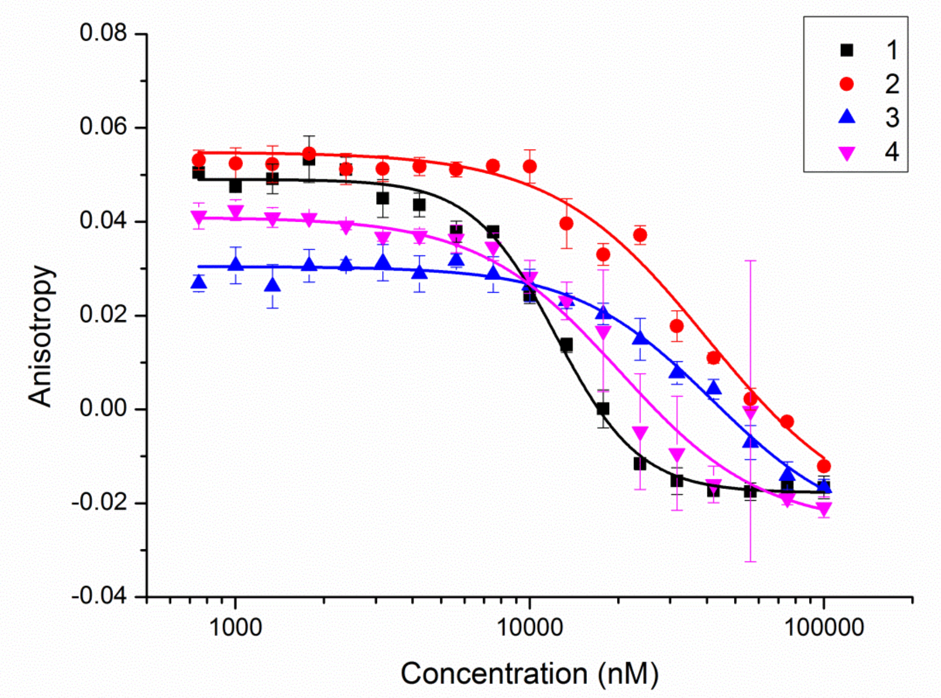

Figure S6 – Fluorescence anisotropy competition assay testing the inhibition of the trimers against the p53/*h*DM2 interaction; unfunctionalised trimer **1** and acid, ethylene glycol and coumarin-functionalised trimers **2**, **3** and **4**. Performed in phosphate buffer pH 7.5, 200 mM NaCl, 0.02 mg/ml BSA. *h*DM2 is fixed as 154 nM and FITC-p53 is at a concentration of 54.5 nM.

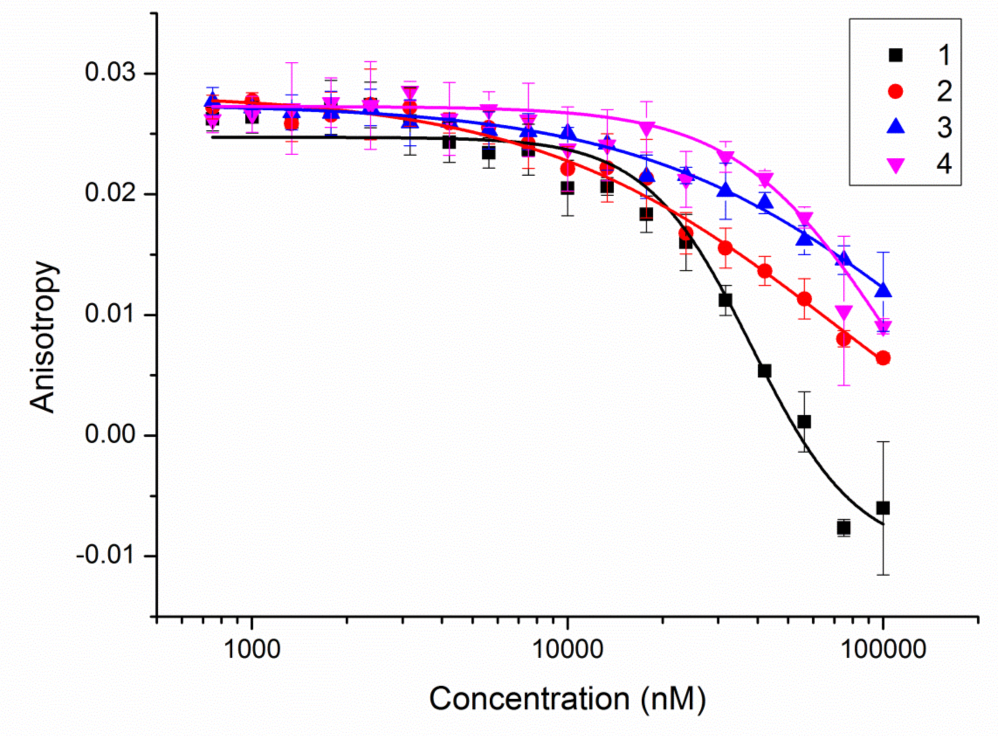

Figure S7 – Fluorescence anisotropy competition assay testing the inhibition of the trimers against the Mcl-1/NOXA-B interaction; unfunctionalised trimer **1** and acid, ethylene glycol and coumarin-functionalised trimers **2**, **3** and **4**. Performed in phosphate buffer pH 7.5, 200 mM NaCl, 0.02 mg/ml BSA. Mcl-1 is fixed as 150 nM and FITC-NOXA-B is at a concentration of 50 nM.

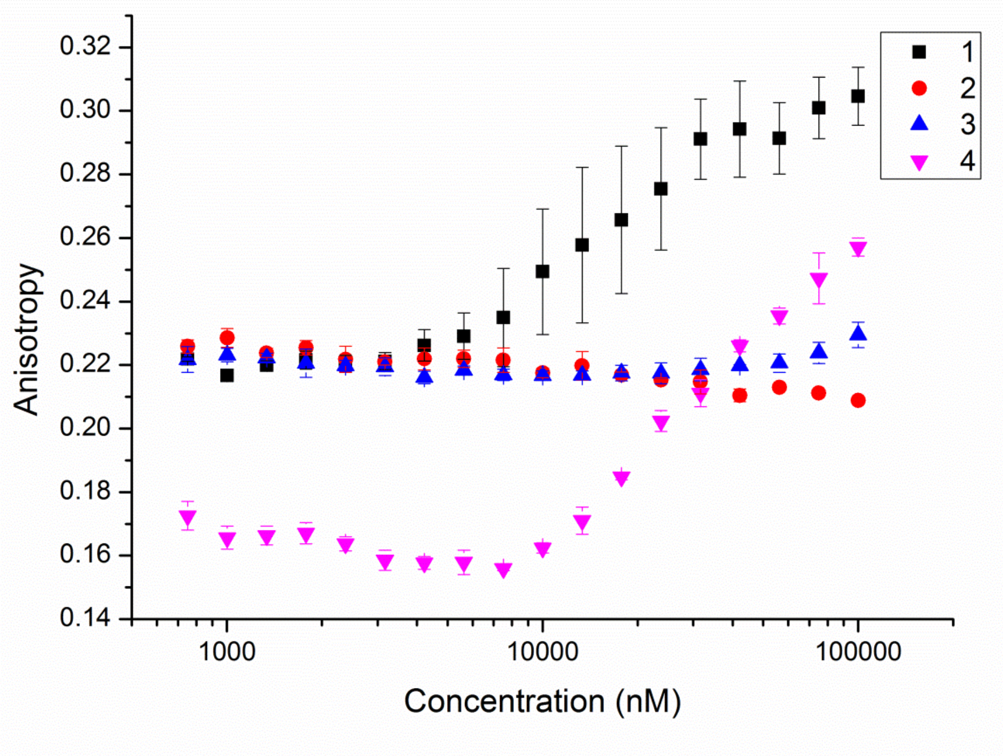

Figure S8 – Fluorescence anisotropy competition assay testing the inhibition of the trimers against the Bcl-x_L/BAK interaction; unfunctionalised trimer **1** and acid, ethylene glycol and coumarin-functionalised trimers **2**, **3** and **4**. Performed in phosphate buffer pH 7.5, 200 mM NaCl, 0.02 mg/ml BSA. Bcl-x_L is fixed as 131 nM and BODIPY-BAK is at a concentration of 43 nM.

The observed increase in anisotropy is likely to be the result of aggregation of the trimer to the BAK tracer and Bcl-x_L complex. The fluorescent peptide is not displaced from the binding site by addition of the helix mimetic.

Direct Binding Assay Development

h$DM2_{17\text{-}126}$ was diluted into buffer across the 24 wells from 400 µM to 36 nM. The fluorescently tagged trimer was then added giving final protein concentrations from 200 µM to 18 nM and a fixed trimer concentration of 50 nM. For control wells the fluorescent trimer was replaced with the equivalent volume of buffer. In all experiments the G factor, or ratio between the efficiency of the S(ame) and P(erpendicular) channels was set to 1. Ligand bound was calculated from Eq. 3 using λ = 2.06. K_d was calculated from Eq. 4.

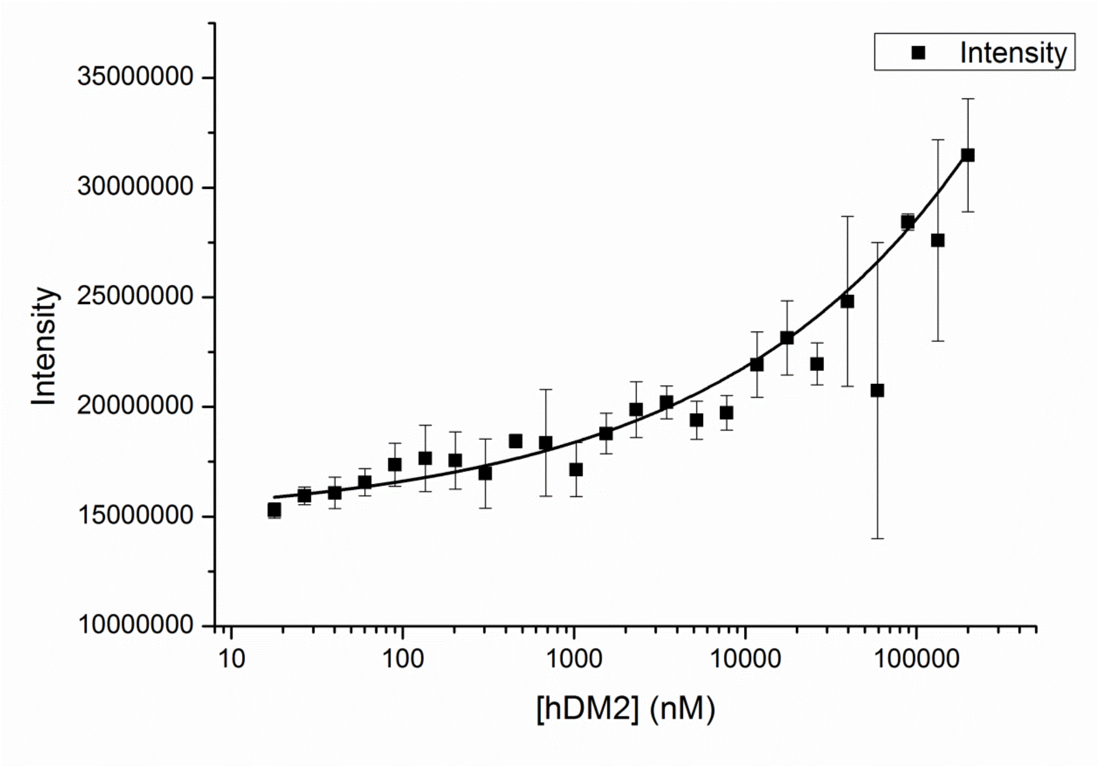

Figure S9 - Change of intensity during addition of *h*DM2 used to calculate λ.

Mcl-$1_{172\text{-}327}$ was diluted into buffer across the 24 wells from 200 μM to 18 nM. The fluorescently tagged trimer was then added giving final protein concentrations from 100 μM to 9 nM and a fixed trimer concentration of 50 nM. For control wells the fluorescent trimer was replaced with the equivalent volume of buffer. In all experiments the G factor, or ratio between the efficiency of the S(ame) and P(erpendicular) channels was set to 1. Ligand bound was calculated from Eq. 3 using λ = 2.31. K_d was calculated from Eq. 4.

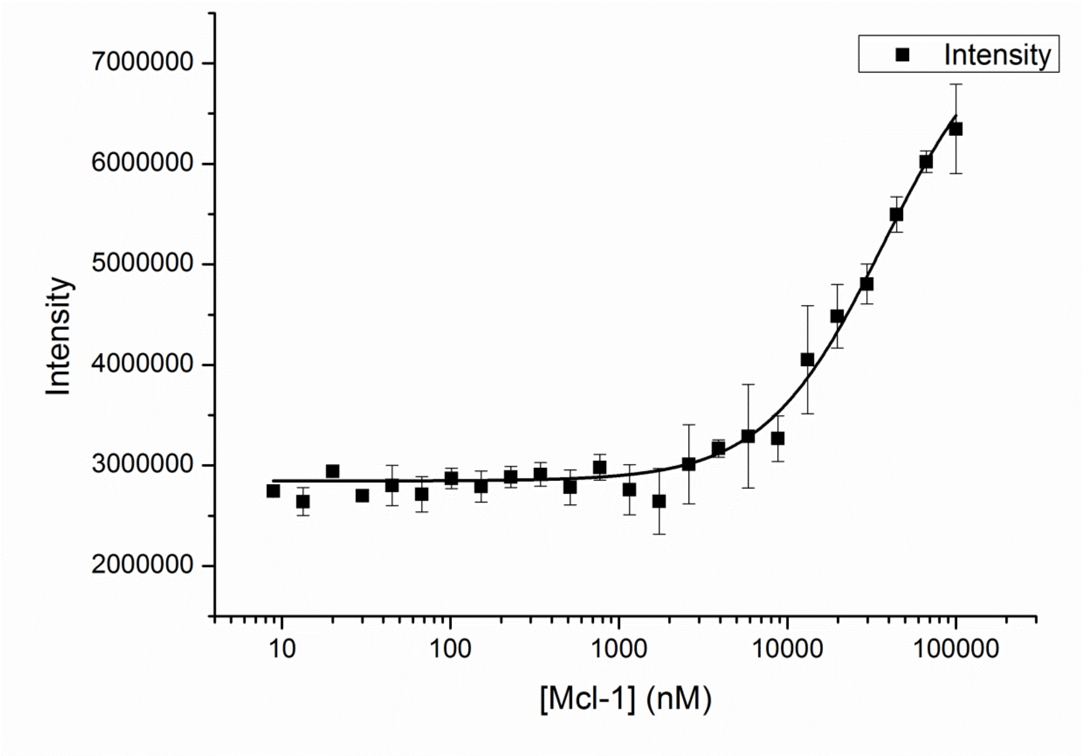

Figure S10 - Change of intensity during addition of Mcl-1 used to calculate λ.

Anisotropy Direct Binding to Bcl-x$_L$

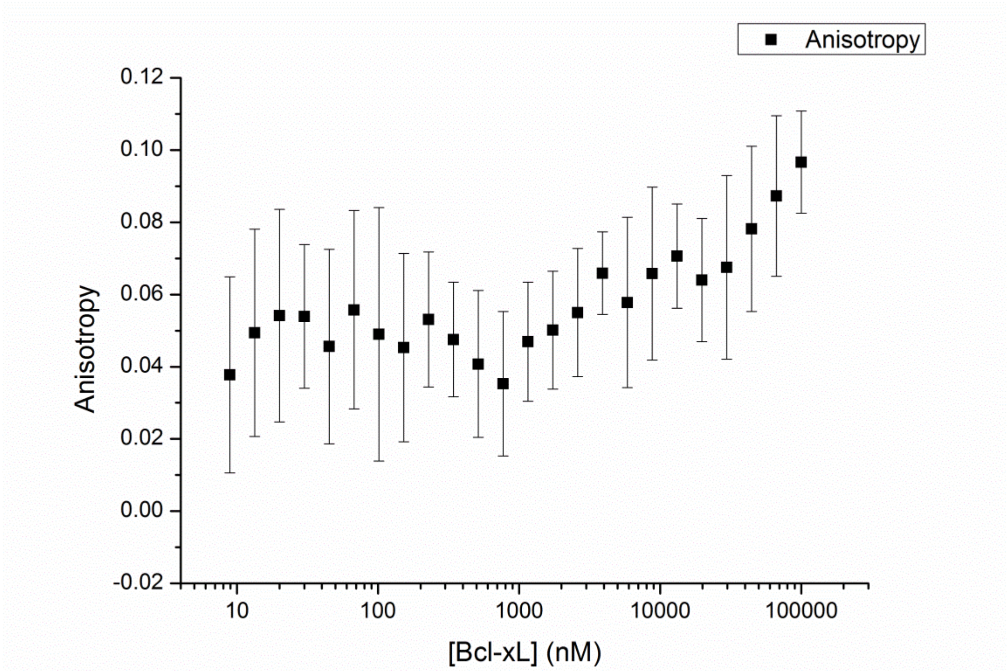

Figure S11 – Anisotropy direct binding of Compound **5** to Bcl-x$_L$ at a fixed concentration of **5** of 50 nM. Performed in phosphate buffer pH 7.5, 200 mM NaCl, 0.02 mg/ml BSA.

Thermophoresis Direct Binding to *h*DM2

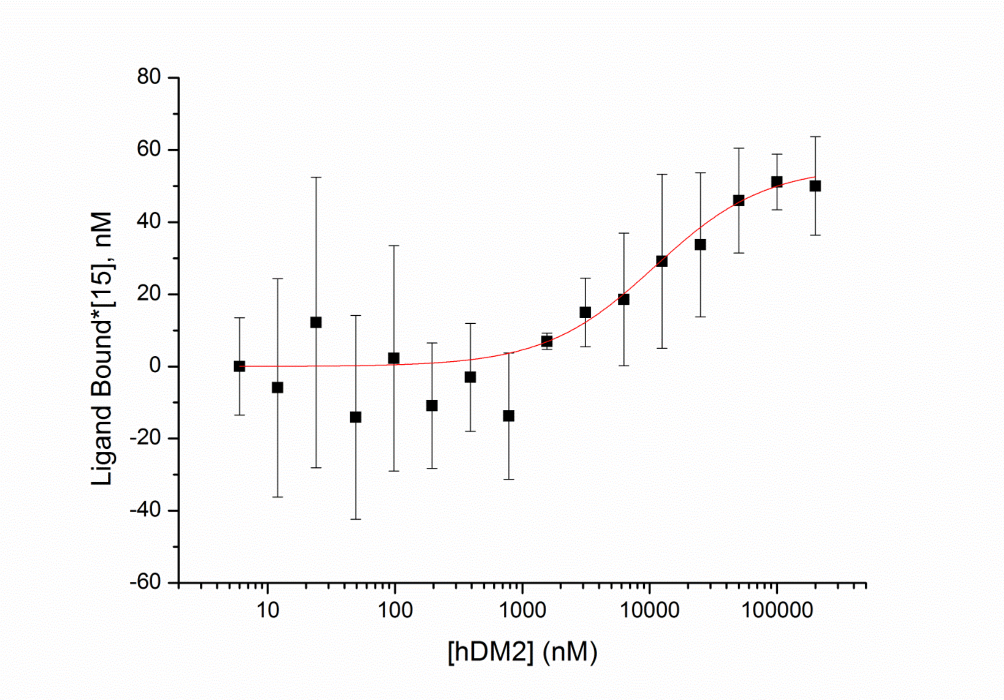

Figure S12 – Direct binding of Compound **5** to *h*DM2 measured using thermophoresis at a fixed concentration of **5** of 50 nM. Kd = 11 ± 2 µM. Performed in phosphate buffer pH 7.5, 200 mM NaCl, 0.02 mg/ml BSA.

The larger errors at lower protein concentration are possibly the result of non-specific binding of the fluorescent trimer to the thermophoresis capillaries.

Fluorescence and Absorbance Characterisation of Trimers 4 and 5

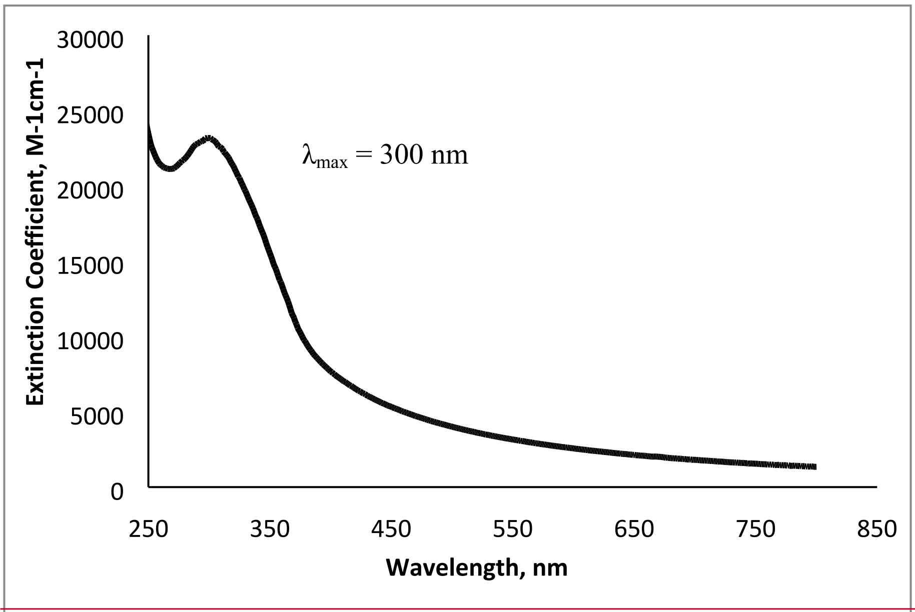

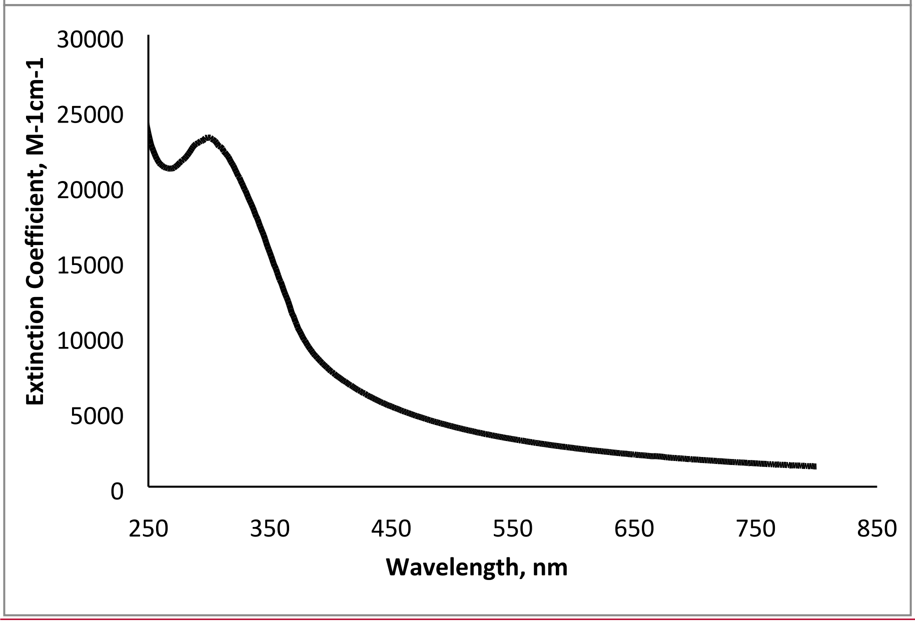

Figure S13 – Extinction coefficient spectra of Compound **4** at 500 µM in PBS buffer pH 7.5

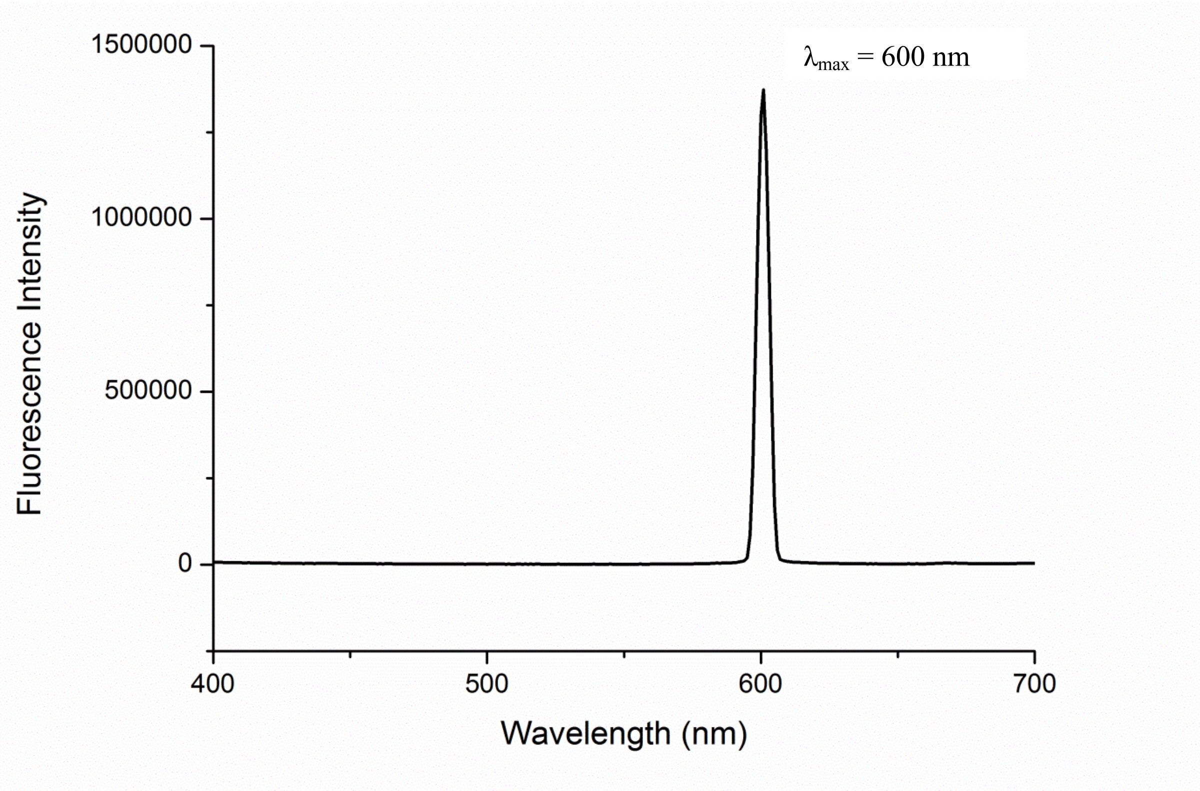

Figure S14 – Fluorescence spectra of Compound **4** at 5 μM in phosphate buffered saline (PBS) buffer pH 7.5

The characteristic maxima for a coumarin fluorophore are an excitation of 365 nm and emission of 445 nm. Neither of these peaks are observed for compound **4**. The sharp peak at 600 nm is possibly the result of electron transfer.

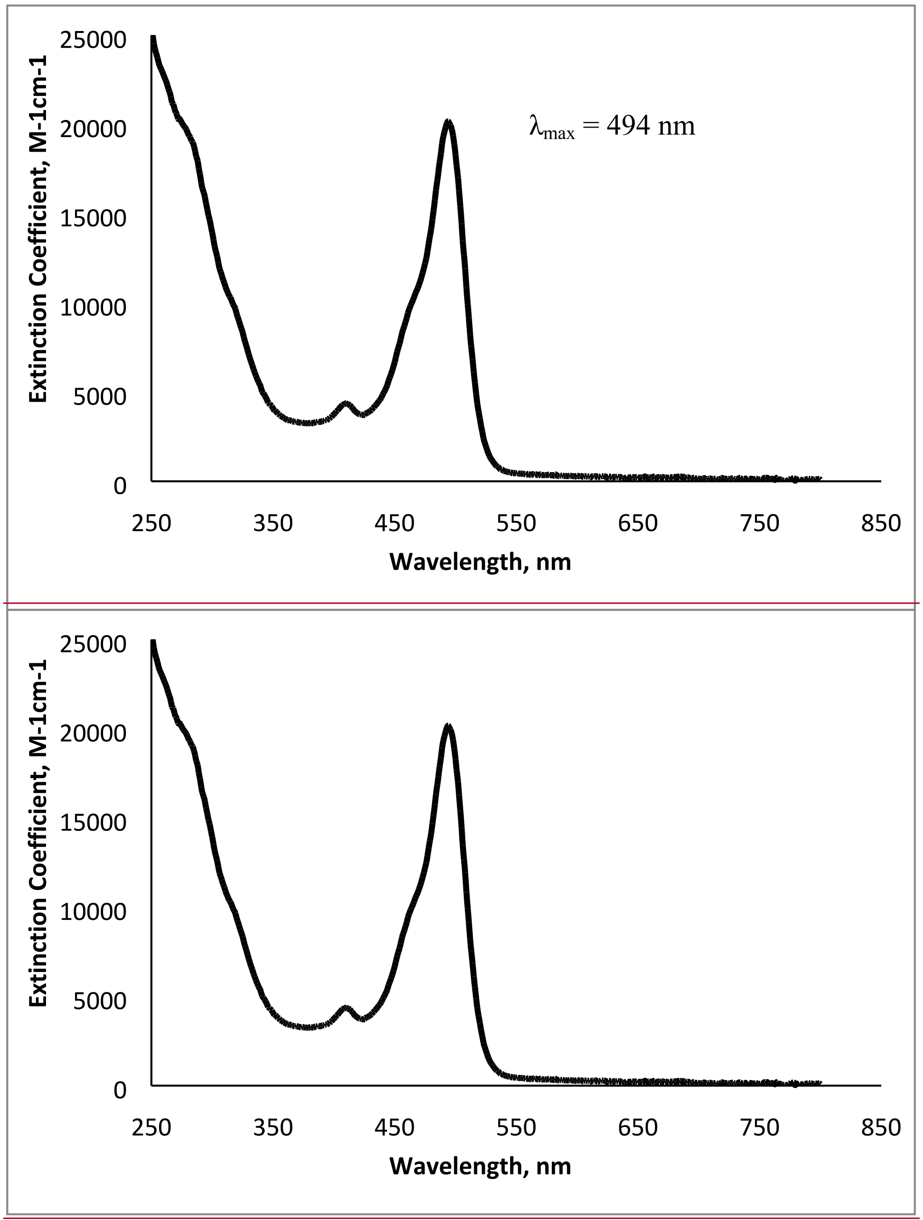

Figure S15 – Extinction coefficient spectra of Compound **5** at 100 µM in PBS buffer pH 7.5

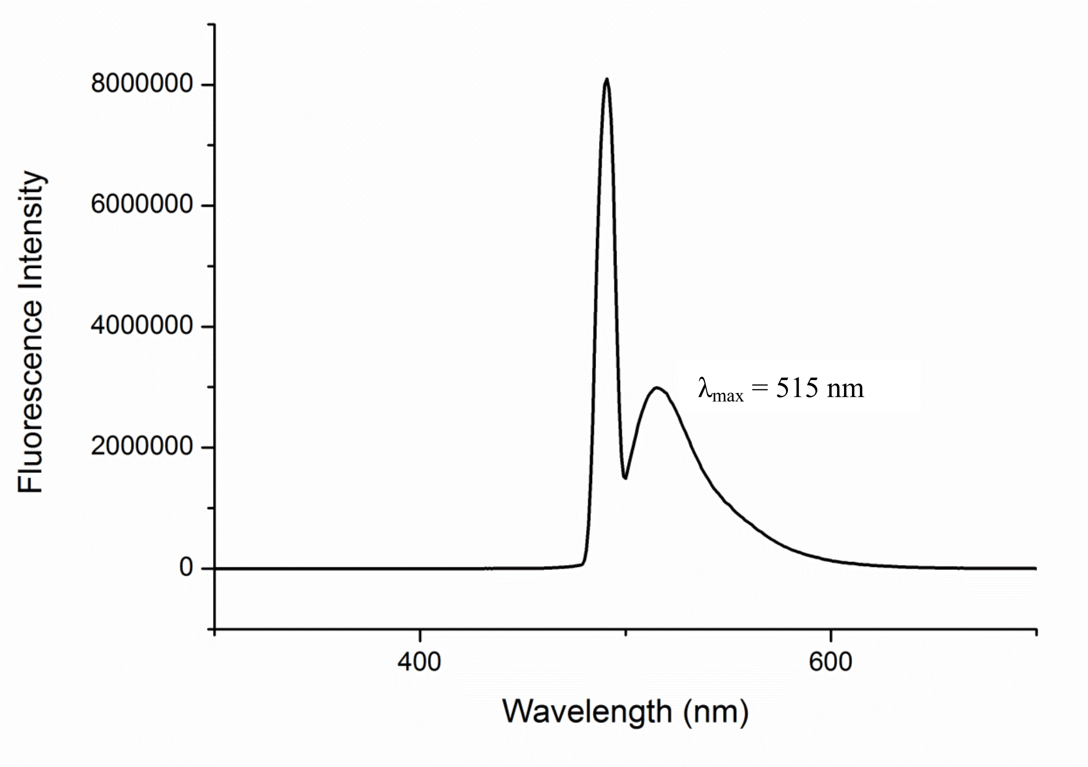

Figure S16 – Fluorescence spectra of Compound **5** at 1 nM in PBS buffer pH 7.5

The characteristic maxima for a fluorescein fluorophore are an excitation of 500 nm and emission of 518 nm.

References

(1) Azzarito, V.; Prabhakaran, P.; Bartlett, A. I.; Murphy, N. S.; Hardie, M. J.; Kilner, C. A.; Edwards, T. A.; Warriner, S. L.; Wilson, A. J. *Org. Biomol. Chem.* **2012**, *10*, 6469-6472.
(2) Yeo, D. J.; Warriner, S. L.; Wilson, A. J. *Chem. Commun.* **2013**, *49*, 9131-9133.
(3) Long, K.; Edwards, T. A.; Wilson, A. J. *Bioorg. Med. Chem.* **2013**, *21*, 4034-4040.
(4) Campbell, F.; Plante, J. P.; Edwards, T. A.; Warriner, S. L.; Wilson, A. J. *Org. Biomol. Chem.* **2010**, *8*, 2344-2351.
(5) Jones, S. P.; Gabrielson, N. P.; Pack, D. W.; Smith, D. K. *Chem. Commun.* **2008**, 4700-4702.
